# Supplementary material for: Unmet need for hypercholesterolemia care in 35 low- and middle-income countries: A cross-sectional study of nationally representative surveys
Source: PLoS Med. 2021 Oct 25;18(10):e1003841. doi: 10.1371/journal.pmed.1003841 (PMC8575312; doi:10.1371/journal.pmed.1003841)
Supplement: S1 Text — (DOCX) [file pmed.1003841.s001.docx]

# S1 Text: Search Methods

We obtained datasets through a systematic online search and request approach. We searched for all countries on the World Bank list of economies from June 2020.

1. STEPS Surveys: STEPS repository and STEPS website

We first identified all countries in which a World Health Organization (WHO) Stepwise Approach to Surveillance (STEPS) survey had been conducted during a year in which the country fell into an eligible World Bank country income category of low-income or middle-income. Prior to the STEPS surveys being made available in the WHO STEPS survey Central Data Catalog in 2019, we systematically requested each eligible STEPS survey from a list of these surveys that the WHO maintains on their website. The research team contacted the responsible party for each survey, based on the information provided on this website. If the contact information was out dated or unavailable, the authors relied on publications utilizing STEPS data and electronic searches of the survey or contact name. For the Caribbean region, country involvement was facilitated by the Caribbean Public Health Agency (CARPHA).

In 2019, additional eligible surveys were downloaded from the Central Data Catalog. The search words used in the WHO Central Data Catalog were: (1) STEPS collection, (2) surveys conducted ≥2008, (3) low-and middle-income countries.

1. Survey Programs and Pooled Data Sources

Whenever the search above yielded no eligible survey, we went on to search the Demographic and Health Surveys (DHS), the WHO Study on Global Ageing and Adult Health (SAGE), the Gateway to Global Aging studies, the NCD Risk Factor Collaboration (NCD RisC), the Global Health Data Exchange (GHDx), and the International Diabetes Federation (IDF) Diabetes Atlas. Potentially eligible surveys were confirmed to be the most recent data available via a google search and subsequently requested.

1. Google Search

Whenever the search above yielded no eligible survey, we conducted a Google search based on the following:

Search engine: Google

Search terms: “[country name]” AND survey AND (“national” OR “population-based” OR “nationally representative”) AND hyperlipidemia OR hypercholesterolemia OR cholesterol OR LDL OR HDL OR lipoprotein OR triglycerides OR triglyceride OR lipid OR lipids)

Number of hits reviewed: Hits reviewed until eligible survey identified, or, in the case of no eligible survey identified, first 30 returned results

Inclusion criteria for a survey:

The survey was conducted during or after 2008; in cases where two surveys were available for a particular country, the most recent was used;

The survey data were made available at the individual level;

The survey contained a biomarker for hypercholesterolemia (total or LDL cholesterol);

The survey was conducted in an upper-middle, lower-middle or low-income country according to the World Bank at the time the survey was conducted;

The survey was nationally representative with a response rate of over 50%;

The survey included a suite of questions that assessed access to a core and comparable group of health services for diagnosis, preventive counselling, and treatment of hypercholesterolemia.

Overall the search process yielded 35 datasets included in our study.
