# Supplementary material for: Unmet need for hypercholesterolemia care in 35 low- and middle-income countries: A cross-sectional study of nationally representative surveys
Source: PLoS Med. 2021 Oct 25;18(10):e1003841. doi: 10.1371/journal.pmed.1003841 (PMC8575312; doi:10.1371/journal.pmed.1003841)
Supplement: S2 Text — (DOCX) [file pmed.1003841.s002.docx]

# S2 Text: Country Categories and Country-specific Sampling Methods

**Country-specific Sampling Weights**

The STEPS datasets include three survey weights for each part of the survey instrument, including the interview (Step 1), physical measurements (Step 2), and biochemical measurements (Step 3), as different samples of participants are included in the three survey parts. STEPS weights are adjusted for the probability of selection, non-response, and differences between the sample population and the target population. Because the analyses in this study rely on lipid measurements as part of the STEPS instrument Step 3, Step 3 weights, referred to as WStep3, were applied in the analyses of STEPS data.

Although most of the datasets included in the study are STEPS surveys (32 STEPS surveys were included), for Chile the 2009/10 National Health Survey, for Seychelles the 2013 National Survey of Noncommunicable Diseases, and for the Marshall Islands the 2017 HYBRID Survey were used. In these surveys also specific weights for biochemical measurements were used where available.

The table below lists the weight variables used for the respective dataset:

| **STEPS Survey:** | **Weight Variable** |
| --- | --- |
| Algeria | wstep3 |
| Azerbaijan | wstep3 |
| Bangladesh | wstep3 |
| Belarus | wstep3 |
| Benin | wstep3 |
| Bhutan | wstep3 |
| Botswana | wstep3 |
| Burkina Faso | wstep3 |
| Costa Rica | wstep3 |
| Ecuador | wstep3 |
| Eswatini | wstep3 |
| Guyana | wstep3 |
| Iran | wstep3 |
| Iraq | wstep3 |
| Kiribati | wstep3 |
| Kyrgystan | wstep3 |
| Lebanon | wstep3 |
| Moldova | wstep3 |
| Mongolia | wstep3 |
| Morocco | wstep3 |
| Myanmar | wstep3 |
| Solomon Islands | wstep3 |
| Sri Lanka | wstep3 |
| St. Vincent and the Grenadines | wstep3 |
| Sudan | wstep3 |
| Tajikistan | wstep3 |
| Timor-Leste | 1* |
| Tokelau | wstep3 |
| Tonga | wstep3 |
| Tuvalu | wstep3 |
| Vietnam | wstep3 |
| Zambia | wstep3 |
|  |  |
| **Non-STEPS surveys** |  |
| Chile - 2009/10 National Health Survey | fexp_fac and fexp_ex |
| Seychelles - 2013 National Survey of Noncommunicable Diseases | wpop |
| Marshall Islands - 2017 HYBRID Survey | 1^+^ |

*Notes*: *As a STEPS survey country, the Timor-Leste data included wstep3. However, the weighted sample characteristics were not consistent with what would be expected from the population of Timor-Leste (or any population) and it was decided that unweighted data were used as the more conservative option.

^+^For Marshall Islands no sample weights were provided as the sampling was said to be representative of the population.

When weights were missing, the average weight was assigned to observations with missing weight values. Further, when observations had to be dropped from the sample because of missing values, for example, in covariates, the survey weights were adjusted proportionally.

In the main analysis of cascades, each country contributes equally to the estimations. To achieve this, the relevant weights of all datasets were rescaled so that the sum of weights in one dataset referring to one country equals 1. In analyses in which surveys should contribute to estimations by their respective country’s population size, weights were rescaled so that the sum of weights in one dataset equals the country’s population size.

**Country-Specific Sampling Methods**

Note: In order to ensure accuracy in reporting, sampling methods are pasted verbatim from specified sources.

**Algeria STEPS 2016-2017**

“A multi-stage cluster sample of households. One individual within the age range of the survey was selected per household. Analysis weights were calculated by taking the inverse of the probability of selection of each participant. These weights were adjusted for differences in the age-sex composition of the sample population as compared to the target population.
Different weight variables are available per Step:
wStep1 - for interview data
wStep2 - for physical measures
wStep3 - for biochemical measures
This allows for differences in the weight calculation for each Step of the survey as the age-sex composition of the respondents to each Step can differ slightly due to refusal or drop out. Additionally, some countries perform subsampling for Step 2 and/or Step 3. When no subsampling is done and response rates do not differ across Steps of the survey, the 3 weight variables will be the same.”

Age range of participants included: 18-69 years

*Source: no report or fact sheet available. Sampling information obtained from: https://extranet.who.int/ncdsmicrodata/index.php/catalog/91/study-description*

**Azerbaijan STEPS 2017**

“A multi-stage cluster sample of households. One individual within the age range of the survey was selected per household. Analysis weights were calculated by taking the inverse of the probability of selection of each participant. These weights were adjusted for differences in the age-sex composition of the sample population as compared to the target population.
Different weight variables are available per Step:
wStep1 - for interview data
wStep2 - for physical measures
wStep3 - for biochemical measures
This allows for differences in the weight calculation for each Step of the survey as the age-sex composition of the respondents to each Step can differ slightly due to refusal or drop out. Additionally, some countries perform subsampling for Step 2 and/or Step 3. When no subsampling is done and response rates do not differ across Steps of the survey, the 3 weight variables will be the same.”

Age range of participants included: 18-69 years

*Source: no report or fact sheet available. Sampling information obtained from: https://extranet.who.int/ncdsmicrodata/index.php/catalog/127/studydescription#page=overview&tab=study-desc*

**Bangladesh: STEPS 2018**

“Sampling design: Samples were collected by multistage, geographically stratified probability based sampling on the basis of Primary Sampling Unit (PSU) developed by Bangladesh Bureau of Statistics (BBS) for census 2011. To ensure generalization and reliability of the survey results to the entire target population in Bangladesh, the sample size calculator as recommended by WHO (sample size calculator STEPS) was used to derive a sample size. The sample size was calculated that is sufficient to produce reliable estimates for all the indicators for men and women and for 4 age-groups (18-24, 25-39, 40-54, 55-69).

[…]

Sampling Frame and primary sampling unit: The sampling frame for the survey was the complete list of Primary Sampling Unit (PSU) i.e. Enumeration Areas (EAs) (about 293,533) covering the whole country prepared by the BBS for the 2011 Population and Housing Census of the People’s Republic of Bangladesh. A PSU is a geographic area covering 100 to 220 households with an average of 113 households. The sampling frame contained information about the PSU location, type of residence (urban or rural), and the estimated number of residential households. A sketch map that delineates the PSU geographic boundaries was available for each PSU. The population coverage rate of this Census 2011 was around 95.85% of the total population. **(Annexure A)**

A special zonal operation was carried out by BBS before 2011 census in 2010 whereby both the urban and rural areas were subdivided with updating of *mauzas (rural)* and *mahallas(urban)* maps with demarcation of PSU boundaries comprising of 100 to 120 (average) houses. Thus based on 2011 census, the sampling frame for the survey was about 293,533 PSUs for both rural and urban areas. The urban stratum included urban and city corporation areas. In Bangladesh, 23.3% of the households are in urban areas; 8.2% are in city corporations, and 15.1% are in other than city corporations.

A new division has been added in 2014 after conclusion of census 2011. So, all the PSUs in the 2011 census were mapped out as per the latest divisions. Thus the sampling frame for STEPS survey 2018 in Bangladesh comprised of 293,533 PSUs: 65,193 urban and 228,340 rural PSUs. Table 2 describes the complete sampling frame by division and by urban and rural areas.

Households in this survey was defined according to BBS as “A dwelling in which persons either related or unrelated living together and taking food from the same kitchen“.

Sampling strategy: This survey used the same 496 PSUs which were sampled and used during a recently concluded GATS-II survey. In GATS Bangladesh 2017 these PSUs were equally allocated to each division (62 each), and within each division, were equally allocated to urban and rural stratum (248 PSUs each to both urban and rural strata). The rural and urban PSUs were arranged by population size in terms of household numbers for both urban and rural stratum in each division. In each stratum (rural and urban), 31 PSUs were selected independently in each division by probability proportional to size (PPS) sampling

A household listing operation was carried out in all the selected PSUs by BBS during GATS- II survey in July 2017 was used and no new household listing was carried out for this survey. As the survey used the same PSUs as used during GATS-II survey, HHs lists prepared by BBS during GATS-II survey in July 2017 served as sampling frame for the selection of households in the second stage.

A fixed number of 20 households were systematically selected from each sampled PSU with an equal probability using a fractional interval technique. Selected households in all the selected PSUs were randomly assigned as “male” or “female” in a ratio that produced equal numbers of male and female households. The 20 selected HHs in a PSU were divided into two groups as 1) male HHs for interview of a male member and 2) female HHs for interview of a female member. All the sampled HHs from each PSU were listed sequentially, and alternate house was assigned as female or male household, with the first household in the list assigned as female household.

Finally, one individual was sampled randomly from all the eligible adults in a participating household using the survey app in android tablets. No replacements and no changes of the pre-selected households were allowed at the implementing stage to prevent bias.“

Age range of participants included: 18-69 years

*Source: National STEPS Survey for Non-communicable Diseases Risk Factors in Bangladesh 2018. Available at:* [*https://apps.who.int/iris/handle/10665/332886*](https://apps.who.int/iris/handle/10665/332886)

**Belarus: STEPS 2015**

“The sampling frame is a collection of data and materials from which are selected for the survey. The optimal sampling frame should be complete, accurate and current. Best of all, the above criteria are met by the results of the population census, which became the basis for constructing the sample for the STEPS study. Census population represents a representative territorial sampling frame in the form

a hierarchical set of parcels grouped in a certain way. Plots censuses are, on average, about the same size. For each site there is a schematic map that provides a clear, non-overlapping demarcation of geographic districts, as well as information on the population and the number of households.

The largest in size is the census area, which includes several instructor sites. The smallest unit in the hierarchical structure of parcels by censuses - enumeration areas. A positive aspect of using enumeration areas as primary sampling units (PSUs) is that they have a small and approximately the same size (each includes about 100 HHs on average). Consequently this, the PSU is a territory within which it is possible to effectively organize field work. To conduct a population census, the territory of the Republic of Belarus was divided into almost 32 thousand enumeration areas. Due to the fact that the last population census in the Republic of Belarus was carried out in 2009, to update the sample, the current data of polyclinics were used, medical outpatient clinics, FAPs and rural Soviet accounting in rural areas.”

Age range of participants included: 18-69 years

*Source: Translated directly from the Belarus STEPS 2016 report. Available at:* [*https://extranet.who.int/ncdsmicrodata/index.php/catalog/100/related_materials*](https://extranet.who.int/ncdsmicrodata/index.php/catalog/100/related_materials)

**Benin: STEPS 2008**

“The STEPS survey in Benin was a population-based survey of adults aged 25-64. A cluster sample design was used to produce representative data for that age range. A total of 6,904 adults participated in the Benin STEPS survey. Recruitment was based on a random five-stage sampling frame. Sixty of 546 districts were randomly selected according to the sizes of their populations. In each district retained, a list of neighborhoods or villages was drawn up and half were selected. In each neighborhood retained, dwellings, households, and then subjects were randomly selected. An investigator went to the center of each neighborhood or village and randomly chose a direction to go before entering one out of every two dwellings. In the dwellings retained, he listed the households and randomly selected one out of two. Within each household, the participant was identified using the Kish method. This procedure was followed until the predetermined sample was obtained for the neighborhood or village concerned. The response rate for the survey was 99%. With respect to the biological data collected in STEP 3, this module was] systematically proposed to six subjects out of ten."

Age range of participants included: 25-64 years

*Source: Houehanou YC, Lacroix P, Mizehoun GC, Preux PM, Marin B, Houinato DS. Magnitude of cardiovascular risk factors in rural and urban areas in Benin: findings from a nationwide steps survey. PLoS One 2015; 10(5): e0126441.*

**Bhutan: STEPS 2014**

“To achieve a nationally representative sample, a multistage sampling method was used to select enumeration areas, households and eligible participants at each of the selected households in three stages. The 2005 National Census was chosen as the basis for the sampling frame, with “Geogs” (blocks) in rural areas and towns in urban areas forming the primary sampling units (PSUs). Since the population distribution for urbanicity is 70:30 (rural:urban), 63 PSUs in rural and 14 PSUs in urban areas were chosen. PSUs were selected through the probability proportionate to size (PPS) sampling using the number of households in each PSU. Two secondary sampling units (SSUs) for every rural PSU and 4 SSUs for every urban PSU were selected. This led to the selection of 126 SSUs from rural and 56 SSUs from urban areas. This was also carried out by PPS sampling, using the number of households in each SSU. A total of 16 households from each SSU (both rural and urban) were selected using systematic random sampling. The sampling frame for this was the list of households with a unique identification number (ID) developed by the enumerators for the survey. At the household level, the Kish sampling method was used to randomly select one eligible member (aged 18–69 years) of the household for the survey. The Kish method ranks eligible household members in order of decreasing age, starting with males and then females, and randomly selects a respondent using the automated program for Kish selection in the handheld personal digital assistant (PDA).”

Age range of participants included: 18-69 years

*Source: National survey for noncommunicable disease risk factors and mental health using approach WHO Steps Approach in Bhutan – 2014. Available at: http://www.who.int/chp/steps/bhutan/en/.*

*Additional reference: World Health Organization Regional Office for South-East Asia. National survey for noncommunicable disease risk factors and mental health using WHO STEPS approach in Bhutan—2014. Geneva: World Health Organization; 2014.*

**Botswana: STEPS 2014**

“Botswana has a population of over 2 million with 27 districts and 4,845 enumeration areas and sample size of 300 enumeration areas with a target population of 6,400 people was systematically drawn from a pool of the whole enumeration areas. Against the identified enumeration areas numbers of households were listed and proportion of participants was calculated from the total sample size required for the country. Finally a computer generated random number was drawn to go into specific households in that specific enumeration area and at the end eligible participants residing in the household were listed into the electronic hand held data assistant (PDA) and at the end a name was picked automatically to participate in the survey.”

Age range of participants included: 15-69 years

*Source: Botswana STEPS report. Available at:*

*https://extranet.who.int/ncdsmicrodata/index.php/catalog/318*

**Burkina Faso: STEPS 2013**

“Sampling methodology: The study was conducted on a sample obtained from a three-stage cluster stratified as recommended by the WHO for STEPS screening surveys. risk factors for noncommunicable diseases. The sampling frame used was that derived from the general census of the population and habitat 2006 (RGPH 2006) and updated in 2010 during the survey Demographic and Health Survey of Burkina Faso (EDS-BF, 2010). This update concerned the enumeration areas (EAs) that correspond to the cluster as part of this study.

Selection of clusters: The choice of clusters was made according to a systematic random selection proportional to their size (in number of households) within strata (regions). To do this clusters were organized by stratum and place of residence (urban / rural). A total of 240 clusters of which 185 were in rural areas and 55 in urban areas were selected for the investigation.

Selection of households: Households were randomly drawn after an enumeration exhaustive list of all households in the cluster. A draw tool designed on Excel by the team. The technique was used in the field for selecting households to investigate. In total, 20 households in clusters were selected to participate in the study.

Selection of individuals: The choice of individuals was made randomly using Kish's method. In total, an individual aged 25 to 64 living in a selected household was fired for participate in the survey.”
Age range of participants included: 25-64 years

*Source, translated from: Rapport de l’enquete national sur la prevalence des principaux facteurs de risques communs aux maladies non transmissibles au Burkina Faso Enquete STEPS 2013. Available at: http://www.who.int/chp/steps/burkina_faso/en/.*

**Chile: NHS 2009-10**

“The sampling frame was constituted from the Population and Housing Census 2002. The design of the study was transversal, with a random sample of complex type households (stratified and multi-stage by clusters) with national, regional and area representation rural / urban. The target population was adults older than or equal to 15 years. The survey had a response rate in the eligible population of 85%. The refusal rate was of 12%. 5,434 people were interviewed. A nurse performed clinical and examinations to 5,043 participants and 4,956 accepted laboratory tests (blood and urine). The total sample loss of the oversized sample was 28% (this including rejection, non-contact and other causes of random loss). The raw sample was designed with overrepresentation of some population groups (older adults, regions other than the Metropolitan Region and rural areas) to increase sample efficiency and homogenize the accuracy of the estimators. The expansion of the sample data is because it grants each participant the weight that corresponds to it according to the design sample and at the same time corrects the distortion of the raw sample, making it coincide with the census population projection for January 2010 for Chilean adults over 15 years of age.“

Age range of participants included: 15 years or older

*Source, translated from: Resumen Ejecutivo: Encuesta Nacional de Salud ENS Chile 2009-10. Available at: http://epi.minsal.cl/encuesta-ens-anteriores/.*

**Costa Rica: STEPS 2010**

“The Costa Rican NCRFSS survey was a cross-sectional survey based on a probabilistic cluster sampling design. The NCRFSS survey was conducted during 2010 under the supervision of the Caja Costarricense de Seguro Social, a government public healthcare provider, and covers the overall adult population aged ≥20 years. Multistage cluster sampling was performed stratified by geographical areas, age groups (20–39, 40–64, and ≥65 years) and gender. The first sample stage was the randomized selection of the country’s geographical areas as primary sample units followed by the random selection of sectors in selected areas as secondary sample units. The random selection of areas and sectors was performed with probability proportional to size; the area or sector size was determined by the population >20 years during 2009, as estimated by the Costa Rican Census and Statistics National Institute (INEC). Households were chosen through a random number generator using dwelling lists obtained from the health technician assistant in every community until all age group and gender strata sample sizes were achieved. A family dwelling was defined as a group of people who share the same table to eat. Survey participants were selected by the Kish method, which samples participants within a household with equal probability of selection, as recommended by the WHO STEPwise methodology. To be eligible for inclusion in the study, subjects had to be ≥20 years of age, permanently residing in the selected homes, and to have provided written consent. Pregnant or lactating mothers and those who were within 6 months postpartum were excluded from the study. Each participant selected for the study was informed of the study objectives and details before agreeing to participate in the investigation. In all, 3653 noninstitutionalized adults were surveyed, with an 87.8% response rate of the eligible population.”
Age range of participants included: 20 years or older

*Source: Wong-McClure R, Gregg EW, Barcelo A, Sanabria-Lopez L, Lee K, Abarca-Gomez L, Cervantes-Loaiza M, Luman ET. Prevalence of diabetes and impaired fasting glucose in Costa Rica: Costa Rican National Cardiovascular Risk Factors Survey, 2010. J Diabetes. 2016 Sep;8(5):686-92.*

**Ecuador: STEPS 2018**

“The STEPS sample design used probabilistic sampling techniques in order to guarantee the geographic representativeness and the study domains of the survey, and to calculate the expansion factors and the errors associated with the sampling.

The target population or universe of study included the total of adults between 18 and 69 years old, disaggregated by men and women, residing in the territory of Ecuador, except Galapagos. According to the INEC population projection, it included 10,249,369 people. The observation unit and elementary unit of analysis were the people between 18 and 69 years of the Ecuadorian territory, except for Galapagos.

[…]

Type and design stages of the sample. The STEPS sample was selected following a probabilistic element sampling scheme with the following three selection stages: i) first stage: selection of Primary Sampling Units (PSU) by stratum; ii) second stage: selection of 12 occupied dwellings within each UPM selected in the first stage; and, iii) third stage: selection of 1 person between 18 and 69 years old per household.”

Age range of participants included: 18 to 69 years

*Source, translated from: Encuesta STEPS Ecuador 2018. Available at: https://extranet.who.int/ncdsmicrodata/index.php/catalog/774*

**Eswatini: STEPS 2014**

“A Multi-stage cluster sampling design was applied. The survey covered all the four regions of the country. The size of the country and the distances between the regions and communities made it possible for the survey to sample a population representing all the 4 regions. The Multi-stage sampling procedure was implemented in the following procedural steps:

Stage 1: All four regions were included as a sampling frame of our Primary Sampling Unit (PSU).The number of the PSUs at this stage ensured precision in the survey estimates and as a result 216 PSUs were selected using probability proportional to size sampling.

Stage 2: The second stage of cluster sampling procedure entailed listing, sorting and random systematic sampling of the Secondary Sampling Units (Households) within the PSUs selected in stage1 where 20 households were selected from each PSU. Based on census data, only households with eligible participants were systematically sampled through random systematic sampling.

Stage 3: At this level, all the eligible participants within a household were sequentially listed into the PDAs and only one participant per household was randomly sampled using KISH method built into the PDAs. The KISH method is a widely used technique that uses a pre-assigned table of random numbers to identify the person to be interviewed.”

Age range of participants included: 15 to 69 years

*Source: WHO STEPS: Noncommunicable Disease Risk Factor Surveillance Report Swaziland 2014. Available at: http://www.who.int/chp/steps/swaziland/en/.*

**Guyana: STEPS 2016**

“A response rate of 66.68% will be selected based on the experience and response rates of other surveys over the years such as the recent Demographic Health Survey 2009. [...] STEPS 3 involve taking blood samples from a proportion of the sample, in this case 50% of the sample, in order to measure raised blood glucose levels and abnormal blood lipids. [...] The STEPS sample will be prepared by the Bureau of Statistics Guyana following the recommended STEPS sample methodology. A multi-stage cluster sampling design will be used. Guyana is divided into 10 administrative regions and within the administrative regions there are seven towns and each region is further divided into enumeration districts. For the STEPS survey 288 enumeration districts will be selected using the population probability sampling method and from each enumeration district 12 households will be selected giving a total sample size of 3456. Further at the household level each participant will be randomly selected by the electronic tablet. For STEP 3 50% of the sample will be randomly selected to participate. A re-listing of some households may also be necessary, such as those interior region locations, in which case in addition to household listings, enumeration districts maps will also be provided so that a re-listing can be done where required.”
Age range of participants included: 18 to 69 years

*Source: STEPwise Approach to Chronic Disease risk factor surveillance (STEPS): Guyana’s Implementation Plan. June 20, 2016. Ministry of Public Health, Guyana.*

**Iran: STEPS 2016**

“The sampling part, which includes determining the sample size and the cluster head, belongs to the pre-study phase and was planned in the form of a specific protocol for sample size and statistical sampling. All experts in the quality control team supervised the finding of samples and cluster heads.

In order to estimate the prevalence rate of the risk factors for non-communicable diseases in the country in 1395, a sampling method proportionate to the population was used, which is a common approach in survey studies. Therefore, the selected sample size was proportionated to the population of that province. On the other hand, for estimating the prevalence of the risk factors in the province, in order to be on the safe side, the smallest sample size for achieving the predicted rates was calculated at 95%. This rate was equal to 384 samples, which was selected as the smallest sample size in the least populated province, Ilam. The required sample size for other provinces was therefore calculated according to the population of that province proportionate to the population of the reference province, Ilam. Besides, to control the non-response error, 10% was added to the calculated sample size in each province.

In order to decrease costs and increase efficiency, for provinces with 800 samples or more, weights were given to their samples. Weight-giving is an effective method used in surveys in order to decrease the sample size. This was achieved in the selected provinces by considering the calculated sample size as half and the sampling weight as double. The total sample size was calculated to be 30150 and to achieve this sample size, sampling from 3015 clusters was required.”

Age range of participants included: 18 and older

*Source: Iran STEPS 2016 report.*

*Available at: https://www.who.int/ncds/surveillance/steps/STEPS_2016_Atlas_EN.pdf?ua=1*

**Iraq: STEPS 2015**

“The sample frame consisted of the population of Iraq of (18+) years for both sexes residing in the urban and rural area. It was based on the results of listing and numbering operation for the year 2009 that covered all governorates. Due to the unstable conditions at the time of the survey three governorates (Naynawa, Salahaddin and Al-Anbar) were excluded. A major challenge confronted was the late demographic change due to population movement, displacement and migration. All permanent residents of (18+) years of age, who were resident in Iraq within one month at the time of implementation of the survey were considered eligible.

A cross‐sectional community based survey covering 15 governorates in Iraq. A Multi-stage cluster sampling technique was depended to select the minimum representative sample size to estimate the prevalence of the risk factors of noncommunicable disease through direct interview, physical examination and laboratory examination of blood samples of study participants. A total of 412 clusters were randomly selected each contain ten households. One subject from each household was randomly selected using KISH table to participate in the survey with a total sample size of 4120. The Sample was designed to provide estimates on a number of indicators on the situation of Noncommunicable diseases risk factors in Iraq at the national level. A national based rather than a governorate based sample is selected. A multi stage cluster sampling was used with stratification to urban and rural areas. Primary sampling units (PSUs) were the blocks, which consisted of 70 households or more before selection.”

Age range of participants included: 18 years and older

*Source: Iraq STEPS 2015 report.*

*Available at: https://www.who.int/ncds/surveillance/steps/Iraq_2015_STEPS_Report.pdf*

**Kiribati: STEPS 2015**

“The second Kiribati STEPS Survey was a population-based survey of 18-69 year olds. The decision was to use three age groups: 18-29, 30-44, 45-69 years for men and women using the following corrections:

• Design Effect of 1.0 (clustering at village and household level)

• 95% confidence interval; p value .05

• 0.7% response rate

• Baseline prevalence percentage indicator: 0.5

• FPC – not applicable

• 6 age-sex groups (18-29 years, 30-44 years, 45-69 years)

As STEPS is intended to be nationally representative, a multi-stage cluster sampling method was used. The STEPS sampling spreadsheet was completed using the most recent census information (2012). The sample was selected in two stages assuming no replacement. At the first stage, a sample of Enumeration Areas (Islands and villages) from each stratum using probability proportional to size (PPS) sampling was selected. In the second stage, a fixed number of households from each selected Enumeration Area using systematic sampling was se­lected. The third stage of sampling selection was done at the household level using the KISH method.

The sampling identified that data collection would be needed on the following islands: Makin, Butaritari, Mara­kei, Abaiang, North Tarawa, South Tarawa,Betio, Maiana, Abemama, Kuria, Aranuka, Nonouti, Tabiteuea North, Tabiteuea South, Arorae, Tabuaeran and Kiritimati. Further details in Annex 3.”

Age range of participants included: 18 to 69 years

*Source: Kiribati STEPS 2015 report. Available at: https://extranet.who.int/ncdsmicrodata/index.php/catalog/724*

**Kyrgyzstan: STEPS 2013**

“A multi-stage cluster sample of households. One individual within the age range of the survey was selected per household.

Analysis weights were calculated by taking the inverse of the probability of selection of each participant. These weights were adjusted for differences in the age-sex composition of the sample population as compared to the target population.

Different weight variables are available per Step:
wStep1 - for interview data
wStep2 - for physical measures
wStep3 - for biochemical measures
This allows for differences in the weight calculation for each Step of the survey as the age-sex composition of the respondents to each Step can differ slightly due to refusal or drop out.”

Age range of participants included: 25 to 64 years

*Source: no report or fact sheet available. Sampling information obtained from: https://extranet.who.int/ncdsmicrodata/index.php/catalog/271/study-description#page=overview&tab=study-desc*

**Lebanon: STEPS 2017**

“A national cross-sectional survey adopting a two-stage cluster sampling design was conducted for Steps 1, 2 and 3. The sampling frames references used were the population distribution in Lebanon 2014, retrieved from the Central Administration for Statistics (CAS) and the Syrian population distribution data 2015, retrieved from UNHCR. 144 clusters were selected for the Lebanese sample and 144 clusters for the Syrian sample. The Primary Sampling Units (PSUs) were cadastral areas (cadasters) and the Secondary Sampling Units (SSUs) were the households. Twenty participants were recruited from each cluster. The latest available population estimates (cadastral data) were used, to randomly recruit PSUs by Probability Proportionate to Size (PPS). To account for the issue of the variability in the cadasters’ sizes, very small cadasters (<200 individuals) were combined with neighboring PSUs before selecting the sample, to enhance the likelihood of finding 20 target participants. On the other hand, cadasters with a large population size that were guaranteed to be sampled at least twice were handled as strata and each stratum were assigned a fixed number of random starting points based on how often it was selected with certainty. This was done using satellite images divided into grids, previously obtained from the Centers for Disease Control and Prevention (CDC) for all Lebanese cadasters.

For the Lebanese sample, the research team relied on the standard Expanded Program for Immunization (EPI) method for a systematic random selection of the households. Accordingly, within each selected PSU, households were identified using a systematic random approach following the WHO-UNICEF-EPI cluster method. The fieldworkers started with the highest floor on the right side of a building. If the household hosted an eligible participant, they proceeded with data collection, if not, they visited a second household which is selected by skipping 5 households. If during sampling, non-Lebanese households were selected, the fieldworker skipped them in a straight line until a Lebanese household was identified. This method has been previously used for national surveys in Lebanon. One participant was randomly selected within each household, using the eSTEPS application. Households were chosen until the target of 20 participants was reached.

The PSUs for the Syrian refugees’ sample were identified, using the most recent available refugee estimates to randomly recruit PSUs by PPS. The same measures aforementioned were done to account for the variation in the cadasters’ sizes. The WHO-UNICEF- EPI cluster method was employed to select households. The fieldworkers targeted Syrian households; accordingly, when during sampling, non-Syrian households were selected, the fieldworker skipped them in a straight line until a Syrian household was identified. One participant was randomly selected within each household, using the eSTEPS application.

For both samples, following STEPS’ team recommendations, sampling of participants was done without replacement, i.e. once a person was selected that person was not replaced with another one. Efforts were made to include all selected households. If the house was unoccupied at the time of the visit or if an adult was not available for an interview at the time of the visit, that house was revisited up to 4 times, with different visiting times. The number of refusals and non-responses was recorded.”

Age range of participants included: 18 to 69 years

*Source: Lebanon STEPS 2016-2017 report. Available at: https://www.who.int/ncds/surveillance/steps/Lebanon_STEPS_report_2016-2017.pdf?ua=1*

**Marshall Islands: HYBRID 2017**

“Stage 1: Households were identified at random according to geographical stratification in Majuro and Ebeye. The country was stratified into two major groups, Urban (Majuro and Ebeye) and Rural (all outer islands). In Majuro and Ebeye, household cluster sampling was used to randomly select households in these areas.

Stage 2: In Majuro and Ebeye, one individual was selected at random from each household using the KISH table method. All adults in Kili, Arno, Wotje, and Jabwor, Jaluit atolls were included in the sample because the adult populations are about 200 each on these atolls.”

Age of participants included: ≥18

*Source: Republic of the Marshall Islands Hybrid Survey Final Report 2018. Available at: https://extranet.who.int/ncdsmicrodata/index.php/catalog/742*

**Moldova: STEPS 2013**

“A total of 4807 randomly selected respondents participated in the survey. They were all aged 18–69 years, and the group comprised both sexes, as well as residents of all districts and the territorial administrative unit “Gagauz-Yeri”, along with Chişinãu and Balti municipalities. The survey did not cover the districts from the left bank of the Nistru River and the municipality of Bender. A two-stage cluster sampling procedure was carried out to select randomly participants from among the target population. Cluster sectors from the 2004 Moldova Population Census were used as a basic unit. Given the differences in lifestyle and disease status between populations in urban and rural areas, the target population was stratified into urban and rural areas of residence for the STEPS survey. At the first stage, within each stratum, primary sampling units (PSUs) (enumeration areas (EAs)) were selected systematically with probability proportional to the 2004 Population Census EAs (measure

of size equal to the number of population in the EAs, provided by the census). Before selection,

the census sectors were sorted geographically from north to south within each stratum, in order to

ensure additional implicit stratification according to geographical criteria. A total of 400 clusters representing 400 EAs were selected from the 10 991 census EAs. These probabilistically selected clusters were used also in Moldova’s DHS conducted in 2005, and the Multiple Indicator Cluster Surveys (MICS) conducted in 2012. Cartographic materials from the Population Census conducted in Moldova in 2004 were not available, thus it was not possible to use them for the STEPS survey. Therefore, for the first stage the probabilistic samples from the abovementioned surveys were used.

Out of the 400 selected clusters, 167 were rural and 233 were urban. The distribution of the sample

of 400 PSUs (EAs) for the DHS/MICS surveys was inversely proportional to the number of population

within each stratum, taking into account that the response rate is lower in urban areas than rural

owing to the smaller average size of the households in urban areas compared with rural areas. Thus,

disproportional allocation with oversampling for urban areas was applied in the STEPS survey. A final

weighting adjustment procedure was carried out to enable estimates at national and urban/rural levels.

At the second stage, 15 households (secondary sampling units (SSUs)) were selected within each of

the 400 PSUs. From the updated list of households used for the MICS 2012 survey, 15 households

were selected randomly per cluster, using the Microsoft Excel**®** random sample tool. A total of 6000

individuals were selected from among the 400 clusters. The Kish method *(17)* was applied for the random selection of one individual aged 18–69 years from each household.”

Age of participants included: 18-69 years

*Source: Republic of Moldova STEPS 2013 report. Available at: https://www.who.int/ncds/surveillance/steps/Moldova_2013_STEPS_Report.pdf*

**Mongolia: STEPS 2013**

“A nationwide, cross-sectional survey was conducted covering 8 districts of Ulaanbaatar city and 21 aimags of Mongolia. A total of 6013 individuals aged 15-64 years old, representing the Mongolian adult population, were involved in the survey.

Sampling: The survey was designed to cover all geographical areas of Mongolia, and a multi stage stratified sampling process was carried out to randomly select participants from the target population. Given the urban vs. rural differences in lifestyle and disease status, the target population was stratified into urban and rural areas and the sample was drawn proportionally based on the target population in each area. Ulaanbaatar, Darkhan and Erdenet cities represented urban areas, while the remaining aimags and soums represented rural areas.

Primary units for Ulaanbaatar, Darkhan and Erdenet cities were khoroos, whereas soums served as primary units for rural areas. The same principle used in the previous STEPS surveys in 2005 and 2009 was applied for sampling unit selections for each stage. From each selected household at the tertiary units of multi-stage cluster sampling in both urban and rural areas, only one individual aged 15-64 years old was randomly selected.

The survey covered a total of 65 cluster sampling units. These units included randomly selected individuals from 32 soums in 21 rural aimags and 33 khoroos in Ulaanbaatar, Darkhan and Erdenet cities. The below Table-1 presents selected clusters, cluster sampling units and the numbers and proportion of participants out of the total population. In order to be able to compare the survey results and findings by urban and rural areas, we conducted sampling based on the principles to select approximately similar numbers of participants from both urban and rural areas.”

Age of participants included: 15-64 years

*Source: Mongolia STEPS 2013 reports. Available at: https://extranet.who.int/ncdsmicrodata/index.php/catalog/615/related_materials*

**Morocco: STEPS 2017**

“One of the essential elements for establishing a probability sampling plan is the constitution an adequate sampling frame. For the purpose of the STEPS survey, the sampling frame used to meet the sampling need was the 2014 master sample, developed by the HCP based on data from the 2014 population and housing census. It has the advantage extrapolate the sample results to the target population and estimate the accuracy desired. The stratification of observation units belonging to any sampling frame makes it possible to design sampling plans ensuring optimal sample size; a significant reduction in costs and a substantial improvement in the accuracy of expected estimators. However, the choice of criteria allowing the population to be divided into homogeneous groups (strata) and having recent and reliable data on these criteria is a task that requires generally considerable efforts (updating the sampling frame) both in terms of methodological than that of data collection.

In Morocco, the particularity of cities containing several social categories for which, synthesizing the vector of heterogeneous demographic and socioeconomic behavior into a representative characteristic makes stratification a difficult task. The stratification adopted was geographical for the two environments according to the weight in terms of households, each of which has a specific stratification: For urban units, the criteria used were the administrative division into regions, provinces / prefectures and the dominant habitat type. As for the rural environment, the primary units were stratified according to the geographical criterion, and the type of relief dominant at the municipal level. “

Age range of participants included: 18 years and older

*Source: Morocco STEPS report [translated online]: https://extranet.who.int/ncdsmicrodata/index.php/catalog/544/study-description*

**Myanmar: STEPS 2014**

“To achieve a nationally representative sample, a multi-stage sampling method was used to select townships, wards and villages, households and eligible participants at each of the selected households.

*Stage 1: Selection of primary sampling units (PSUs)*

Administratively, Myanmar is divided into 330 townships. A township is subdivided into

wards for urban settings and village tracts and then villages for rural settings. The list of townships has been used as the sampling frame at the first stage of sampling. Townships form the Primary Sampling Units (PSUs). Out of the total 330 PSUs, 52 PSUs were selected using Probability Proportionate to Size of population in each PSU (PPS).

*Stage 2: Selection of Secondary Sampling Units (SSUs)*

From each selected PSU (township), 6 SSUs (wards and villages) were chosen using probability proportionate to population size, totaling 312 SSUs for the whole country.

*Stage 3: Selection of eligible participants at household level*

From each selected SSU (ward/village), 30 households were selected using systematic random sampling. The sampling frame for this sampling is the list of households with unique identification number (ID) developed from a recent listing of households available from the Basic Health Staff.

*Stage 4: Selection of eligible participants at household level*

One eligible participant (aged between 25 and 64 years) in the selected

households was recruited for the survey. The Kish sampling method was used to randomly select one eligible member of the household. Using the Kish Method, eligible participants (adults aged 25 to 64 years) in each household were ranked in order of 8 decreasing age, starting with males then females, then randomly selected using the automated program for Kish selection in the handheld PDA. Each PSU (township) was estimated to contribute 180 participants, totaling 9,360 participants for 52 selected townships for the whole country. In actual study, the total sample size was 8757 participants.”

Age range of participants included: 18 years and older

*Source: STEPwise approach to chronic disease risk factor surveillance report 2014. Available at: https://www.who.int/ncds/surveillance/steps/myanmar/en/*

**Seychelles: National Survey of Noncommunicable Diseases 2013**

“The survey was performed in a sex and age stratified random sample of all adults aged 25‐64 years of Seychelles between October and December 2013 on Mahé and during 2 weeks in February 2014 in the islands of Praslin and La Digue. These three islands account for >98% of the total population of Seychelles. The eligible sample was extracted from the population registry. The survey was attended by 1240 adults, with a participation rate of 73%. Participants were invited to attend the survey on selected days in study centers located in Mahé, Praslin, and La Digue. All the eligible participants who did not attend were actively traced using (telephone, local administration, announcements on radio, etc) and invited to attend the survey. Since participants were randomly selected from the general adult population, findings of the survey can be inferred to the general adult population of Seychelles.”

Source: *National Survey of Noncommunicable Diseases in Seychelles 2013‐2014 (Seychelles Heart Study IV): methods and main findings. Available at:* [*http://www.who.int/chp/steps/seychelles/en/*](http://www.who.int/chp/steps/seychelles/en/)*.*

**Solomon Islands: STEPS 2015**

“A multi-stage cluster sample design was used to produce representative data. Analysis weights were calculated by taking the inverse of the probability of selection of each participant. These weights were adjusted for differences in the age-sex composition of the sample population as compared to the target population.

Different weight variables are available per Step:
wStep1 - for interview data
wStep2 - for physical measures
wStep3 - for biochemical measures
This allows for differences in the weight calculation for each Step of the survey as the age-sex composition of the respondents to each Step can differ slightly due to refusal or drop out.”

Age range of participants included: 18 to 69 years

*Source: no report or fact sheet available. Sampling information obtained from: https://extranet.who.int/ncdsmicrodata/index.php/catalog/710/study-description#page=overview&tab=study-desc*

**Sri Lanka: STEPS 2014/15**

“A national cross-sectional survey was conducted using the WHO STEPwise survey protocol to obtain nationally representative estimates from the adult population, aged 18 to 69 years, in Sri Lanka.

2.2 Study population

The target population of the study was adults aged 18 to 69 years old residing in Sri Lanka.

2.3 Inclusion criteria

All individuals aged 18 to 69 years of age, and residing in the particular address for more than 6 months were included.

2.4 Exclusion criteria

Individuals who fall into following categories were excluded from the survey. • Who were living in the particular address for less than 6 months
• Who were foreigners and living in the country on a temporary basis • Who were mentally unfit

• Who were physically too frail to be included in the study

2.6 Sampling method

A multi stage cluster sampling method was used to select a nationally representative sample from the total population. Department of Census and Statistics of Sri Lanka performed the selection of the study sample. Population of each divisional secretariat (DS) divisions as per the preliminary results of the Census done in 2012 was used for sampling.

Sri Lanka is administratively divided in to 9 provinces and 25 districts. Each district is divided to Divisional Secretariat (DS) areas. Each DS area is divided to many Census Blocks, and each Census Block consists of many households.

Primary sampling unit (PSU)

The primary sampling unit (PSU) was a Divisional Secretariat (DS) area. Out of 331 DS areas available, 80 DS divisions were selected using proportionate to the size (PPS) sampling.

Secondary sampling unit (SSU)

A census block was considered as a SSU. From each DS division (PSU), six secondary sampling units (SSU) were selected using the proportionate to the size (PPS) sampling technique. Therefore, a total of 480 SSUs or census blocks were selected from 80 PSUs.

Tertiary sampling unit (TSU)

Number of houses in each census block depends on the area density and the population density in each DS division. Tertiary sampling unit (TSU) was the household and 15 households from each CB by random systematic sampling by the Department Census and Statistics. Therefore, a sample of 7200 (80x6x15) households were selected. In some instances, there were more than one household living in one house. People who are cooking and eating together were considered as one household. Whenever there were more than one household in a house, one household was selected randomly to be included in the study.

Selection of participants

Only one participant from each household was included in the survey. All the eligible members in the selected family were listed in descending order according to the age. Once this was done, these data was fed to the personal digital assistants (PDAs). The PDAs then automatically selected the eligible participant using the Kish method. “

*Source: Non Communicable Disease Risk Factor Survey Sri Lanka 2015 Report. Available at: https://www.who.int/ncds/surveillance/steps/sri_lanka/en/*

**St. Vincent & the Grenadines: STEPS 2013**

“The survey covered the entire island St. Vincent and the Grenadines, and was conducted using the following zoning categories:

1) Mainland (St. Vincent)

2) Northern Grenadines (Bequia and Mustique)

3) Southern Grenadines (Canouan and Union Island)

The sample size was proportionately divided between the three main reporting strata (St.Vincent/Northern Grenadines/Southern Grenadines). The country’s most recent age breakdown based on the 2001 national census by St. Vincent was used to approximate the adult population 18-69 years by Island grouping. The survey was stratified by sex, age groups 18-29, 30-44 and 45-69 years and by geographical location – St. Vincent, Northern Grenadines and Southern Grenadines.

A three-stage cluster sampling approach was used. Enumeration districts were randomly selected using Probability Proportional to Size (PPS) from the sampling frame. A total of 199 enumeration districts were selected. The sampling frame was developed using the number of households per enumeration district taken from the 2012 preliminary census report; enumeration districts had been subsequently revised (2010-2011) so that no enumeration district containing more than 150 Households would be randomly selected from the selected enumeration districts. The number of households per enumeration district to be selected was 26. Where an enumeration district had been split into 2 or more new enumeration districts the number of households in the previously defined enumeration district was divided equally between the newly revised enumeration districts. The household list for each selected enumeration district was updated prior to selection of households during a re-listing exercise. This was necessary as the existing household listing for each enumeration district was outdated.

Eligible persons at the household level were randomly selected using the Kish method. If no one was present in the selected household, a notification of visit card was left and the interviewer revisited. There was a total of three visits to the household before it was listed as non-response (one initial recruitment visit and two call backs). The interviewer then moved on to the next house on the list in the original order. Although the person selected for interview were to be at least 18 years and not older than 69 years on the last birthday, there were a few instances where some participants were turning 18 or 70 years; those cases were addressed during data cleaning.

Biological samples, testing and Nutrition intake (24 hour recall):

Fifty percent (50%) of the survey participants were asked to provide a biological specimen (finger prick) for Glucose and cholesterol testing using Glucose and Lipid Sampling Kits and respond to the nutrition intake (24 hour recall). The biological sample was only collected with participants’ explicit consent; the samples were not stored or used for additional undetermined or undisclosed future testing to which respondents did not agree at the time of participation.”

Age range of participants included: 18 to 69 years

*Source: WHO STEPS: Noncommunicable Disease Risk Factor Surveillance. Report for St. Vincent & the Grenadines 2015. Available at: http://www.who.int/ncds/surveillance/steps/stvincent/en/*

**Sudan: STEPS 2016**

„A four-stage cluster sampling design was implemented. The four sampling stages were; 1) selection of states from the six regions 2) selection of clusters (a cluster was a Popular Administrative unit), 3) selection of households and 4) selection of eligible individuals. First Stage (State): Administratively Sudan is divided into 18 states which are grouped in six regions, (North, East, Khartoum, Central, Kordofan and Darfur region (Table 1). States were randomly selected from each region. No geographical areas or populations were excluded from the sampling frame. Thus 11 states were selected, probability proportional to the size, to represent the six regions. A list of the selected states is shown in Table 2.1. Second Stage (Cluster PAU): The Popular Administrative Units (PAU) is the smallest geographically border unit. These were defined as the ‘cluster’ in the region. Clusters were randomly sampled from all PAUs, from both urban and rural strata, according to probability proportional to size in each state, and urban/rural distribution. The PAUs inaccessible due to security conditions were not excluded from the sampling frame, because within certain areas the security status was continuously changing. However, it was planned that if a PAU was found to be inaccessible at survey time, it should be replaced. However, no replacement was required during this survey. Third Stage (Household): Within the selected PAUs, all households (HH) were included in the sampling frame. Accordingly (HH) were selected using systematic random methods.

Fourth Stage (Individual): The members of the household were first listed in the mobile application (customized software). The inclusion criteria for the listed members were: all individuals aged between 18 to 69 years, from both sexes, irrespective of his health status and living in the selected household for a minimum of 6 weeks. The application was then run and it randomly selected the individual who will be selected to participate in the study.“

Age of participants included: 18-69 years.

*Source: Sudan STEPS 2016 report. Available at: https://www.who.int/ncds/surveillance/steps/Sudan_STEPwise_SURVEY_final_2016.pdf?ua=1*

**Tajikistan STEPS 2016**

“A multi-stage cluster sample of households. One individual within the age range of the survey was selected per household.

Analysis weights were calculated by taking the inverse of the probability of selection of each participant. These weights were adjusted for differences in the age-sex composition of the sample population as compared to the target population.

Different weight variables are available per Step:
wStep1 - for interview data
wStep2 - for physical measures
wStep3 - for biochemical measures
This allows for differences in the weight calculation for each Step of the survey as the age-sex composition of the respondents to each Step can differ slightly due to refusal or drop out.”

Age range of participants included: 18-69 years

*Source: report not available. Sampling information obtained from: https://extranet.who.int/ncdsmicrodata/index.php/catalog/270/study-description#page=sampling&tab=study-desc*

**Timor-Leste: STEPS 2014**

“Note: Data from Census 2010 were used for all sampling considerations. Even though pl^4,22^anning and mapping for 2015 Census is ongoing, data from the Census will only be available after July 2015.

STEP 1: Selection of Enumeration Area

(1) List of EA with number of HH by district for Census 2010 was obtained from the Directorate of Statistics. There are 1826 EAs in Timor-Leste. Out of these, 150 EAs were selected.

(2) The number of EAs to be selected from each district was based on their proportion in the country’s population as per Census 2010.

(3) The numbers of Households (HH) per EAs varied from 0 to more than 300. Therefore, probability proportion to size (PPS) was used.

(4) For each district, the EAs were arranged in ascending order of HH size.

(5) Sampling interval was obtained by dividing the total number of HH in the district by the number of EA to be selected from that district.

(6) A random number was generated between one and the sampling interval for that district, using tools available at random.org.

(7) The EA where that random number fell was the first EA to be selected.

(8) Subsequently, the sampling interval was added to the random number and the EA where this new number fell was selected. For the next number, the sampling interval was added to the number and so on, till the population of HH was exhausted or target number of EA achieved.

(9) This was done separately for each district.

(10) The final list was compiled and had 150 EAs. These are spread over about 125 sucos.

STEP 2. Selection of Households in an Enumeration Area

Listing the house numbers to be visited

(1) It was decided to use the 2010 HH size of each EA. Based on past experience, it was expected that the increase would be on an average about 4–5%.

(2) The list of households to be selected by enumerators was decided centrally.

(3) Sampling interval was calculated by dividing the total number of households in the EA by 18.

(4) The first HH number was selected randomly by reading the last two digits of a currency note. If the number represented by the two digits was more than 18, the last digit was taken into consideration. For each EA, a different currency note was used. This could also be done it by using the tool at random.org. or by draw of lots.

(5) The subsequent HH are identified by adding the sampling interval as was done for selection of EA.”

Age range of participants included: 18 to 69 years

*Source: Timor-Leste STEPS Survey Report, [online] at http://www.who.int/entity/chp/steps/Timor-Leste_2014_STEPS_Report.pdf?ua=1*

**Tonga: STEPS 2017**

“An initial sample of 4,500 individuals (respondents) between the ages of 18 to 69 years old was targeted to undertake the STEPS survey for 2017 in Tonga.

Because it is important to compare the results by island divisions (national level), it is required with importance to produce the estimates in the divisional level (National Level). Therefore the sampling fractions will be adjusted from its proportional to the size (number of households) to have higher sampling fraction (coverage) for the smaller size island division as shown in the following table:

Pop Census STEPS sample
Island Total HH ideal sample size coverage Number of Selected Blocks

1 Tongatapu 12,953 3240 25.0% 270 2 Vava'u 2,715 684 25.2% 57
3 Ha'apai 1,179 288 24.4% 24
4 Eua 885 228 25.8% 19

5 Niua 273 60 22.0% 5
Total 18,005 4,500 25.0% 375

The final sample numbers presented in the table above were rounded such that they were divisible by 12 (an enumerators workload) to accommodate field logistics. As such the sample size is recorded to 4,500. The sample was selected independently within each of the 5 target areas.

The sampling in each area was then undertaken using a three-stage process. The first stage involved the selection of census blocks using Probability Proportional to Size (PPS) sampling, where the size measure was the expected number of households in that block. For the second stage, a fixed number (twelve) of households were selected from each selected census block using systematic sampling. The household lists for all selected blocks were updated just prior to the second stage of selection. Once the selected 12 households are found, then the list of household members age 15 to 64 by gender will be recorded. The final stage will be to use the Random Sample Generator (Android Application) to randomly select one person from the household to be enumerated so that it captures the required composition of the sample with specific age-group distribution and gender.“

Age range of participants included: 18 to 69 years

*Source: Tonga STEPS Survey 2017 Sampling Design. Available at:*

*https://pacificdata.org/data/dataset/spc_ton_2017_steps_v02_m/resource/261f0a3c-4979-4a42-a560-1b103c617a42?inner_span=True*

**Tokelau: STEPS 2014**

“The 2005 Tokelau STEPS survey was design as a whole population-based cross-sectional study of 15-64 year olds in the three atolls. There was no sampling involved in this survey as all eligible individuals were targeted for participation.”

Age range of participants included: 15 to 64 years

*Source: Tokelau NCD Risk Factors STEPS Report. Available at: https://www.who.int/ncds/surveillance/steps/STEPS_Report_Tokelau.pdf*

**Tuvalu: STEPS 2015**

“The Tuvalu STEPS Survey was a population based cross-sectional survey of 18-69 year olds. Analysis weights were calculated by taking the inverse of the probability of selection of each participant. These weights were adjusted for differences in the age-sex composition of the sample population as compared to the target population.

Different weight variables are available per Step:
wStep1 - for interview data
wStep2 - for physical measures
wStep3 - for biochemical measures
This allows for differences in the weight calculation for each Step of the survey as the age-sex composition of the respondents to each Step can differ slightly due to refusal or drop out. Additionally, some countries perform subsampling for Step 2 and/or Step 3. When no subsampling is done and response rates do not differ across Steps of the survey, the 3 weight variables will be the same.”

Age range of participants included: 18 to 69 years

*Source: no report or fact sheet available. Sampling information obtained from: https://extranet.who.int/ncdsmicrodata/index.php/catalog/639/study-description#page=overview&tab=study-desc*

**Vietnam: STEPS 2015**

“At the same time of STEP survey, MOH also conduct the Global Adult Tobacco Survey (GATS) at the same scale, location, and study subjects (>15 years for GATS and 18-69 for STEPS). The sampling of STEPS was done in as part of the sampling for the (GATS) conducted in combination manner to save time and resources for these two surveys. Applied the multi-stages complex sampling process, the sampling process done by GSO was as follow: • Sampling of clusters (EA) In the first stage of sampling, the primary sampling unit (PSU) was an enumeration area (EA). There are about 170,000 EAs in the whole Viet Nam and the average number of households in each EA is different between urban and rural areas. An average number of households in an urban EA and a rural EA is 133 households and 120 households, respectively. Sample of EAs were selected from the master sample frame. The master sample frame was a cluster frame made by the GSO based on the frame of Population and Housing Census 2009 and updated with data of 2014. Based on the Population and Housing Census data 2009, GSO prepared a 15% of master sample to serve as a national survey sampling frame. The master sample frame contains 25,500 enumeration areas (EAs) from 706/708 districts of Viet Nam (2 island districts were excluded from the GSO master sample frame). The master sample frame of GSO was divided by two stratification variables: urbanization (1 = urban; 2 = rural) and district group (1 = district/town/city of province; 2 = plain and coastal district; 3 = mountainous, island district). It means that the master sample frame was divided into 6 sample frames or 6 strata. The probability proportional to size (PPS) sampling method was used to select sample of EAs from 6 strata of master sample frame. The final sample of GATS included 315 EAs in the urban and 342 EAs for the rural. From these 657 EAs, 315 EAs were systematically selected for STEPS.

Sampling of households At the second stage of sampling, 10% households in each EA were selected. Thus, 15 households from the selected urban EA and 14 households from the selected rural EA were chosen using simple systematic random sampling. The total households for STEPS 2015 were 4,651 households.

Sampling of individuals: One eligible person is then randomly selected from each selected household for the STEPS 1 interview. The selection of individual is automatically done by the PDA program after eligible household members are entered into the PDA. The selection probability of an eligible individual was calculated as a product of selection probability for each stage. The sampling base weight for an eligible individual was the inverse of the selection probability shown above.”

Age range of participants included: 18 to 69 years

*Source: National Survey on the Risk Factors of Non-communicable diseases (STEPS) Viet Nam Report 2015. Available at: https://www.who.int/ncds/surveillance/steps/viet_nam/en/*

**Zambia: STEPS 2017**

“To ensure that the sample reflected the entire country of Zambia, a multi-stage cluster sampling technique was used to select a nationally representative sample of adults in Zambia aged 18 to 69 years. It was decided to utilize the household listing from the Zambia PopulationBased HIV Impact Assessment (ZAMPHIA) - a household-based national survey that was conducted between March and August 2016 in order to measure the status of Zambia’s national HIV response. ZAMPHIA offered the most pragmatic up to date and accessible national household listing to be used as the sampling frame for this survey. The ZAMPHIA survey included 60,581 households drawn from 1,103 clusters referred to in this report as standard enumeration area (SEA) (Table 2.4.1). Thus the sample drawn for the STEPS survey was a subsample of the households selected for the ZAMPHIA survey. In the first stage of sampling, SEAs were selected from each province using probability proportional to size (PPS). In the second stage, 15 households in rural SEAs and 20 households in urban SEAs were selected systematically using appropriate sampling interval based on the number of households in that SEA. These households constituted the final list of households for the STEPS survey prepared for the field investigators (FI). In the third stage, while the FI approached the household and sought consent, all eligible members in the household were entered into the Android-based devise used for the survey. The device then selected one member from the eligible members using a simple random sampling technique. The selected member was then interviewed having gone through the ethical process of consent after being provided with information on the survey. If the selected member was not available, a scheduled visit was made. If the selected member could not be reached after two scheduled visits he or she was considered as non-response. There was no replacement strategy so as to maintain the integrity and representativeness of the sample.”

Age range of participants included: 18 to 69 years

*Source: STEPS 2017 Report. Available at:* [*https://extranet.who.int/ncdsmicrodata/index.php/catalog/620*](https://extranet.who.int/ncdsmicrodata/index.php/catalog/620)
