# Supplementary material for: Unmet need for hypercholesterolemia care in 35 low- and middle-income countries: A cross-sectional study of nationally representative surveys
Source: PLoS Med. 2021 Oct 25;18(10):e1003841. doi: 10.1371/journal.pmed.1003841 (PMC8575312; doi:10.1371/journal.pmed.1003841)
Supplement: S3 Text — (DOCX) [file pmed.1003841.s003.docx]

# S3 Text: Country-specific Lipid Measurement Methods

In seven countries biomarkers were measured via blood samples sent to a laboratory; in 22 countries biomarkers were measured with a point-of-care device; in six countries, the biomarker measurement method could not be identified.

| **Measurement** | **Country** |
| --- | --- |
| Accutrend GC | Tokelau |
| Accutrend Plus | Tuvalu |
| CardioCheck PA | Belarus, Benin, Bhutan, Burkina Faso, Ecuador, Eswatini, Kiribati, Moldova, Morocco, Solomon Islands, Sri Lanka, St. Vincent & the Grenadines, Sudan, Timor-Leste, Tonga, Vietnam, Zambia |
| Konelab 30i | Seychelles |
| Laboratory | Bangladesh, Chile, Costa Rica, Guyana, Iran, Iraq, Lebanon |
| Prima Home Test | Mongolia |
| SD LipidoCare Analyzer | Myanmar |
| Unknown | Algeria, Azerbaijan, Botswana, Kyrgyzstan, Marshall Islands, Tajikistan |

In the following, please note: In order to ensure accuracy in reporting, lipid measurement methods are pasted verbatim from specified sources.

**Algeria: STEPS 2016**

No further information available

**Azerbaijan: STEPS 2017**

No further information available

**Bangladesh: STEPS 2018**

“An appointment/clinic card was also given to every participant for biochemical measurement containing fasting instructions. This card contained the appointment date, time and place for blood glucose and lipid measurement. On the given date and time, the enumerators made biochemical assessment (Fasting blood glucose and lipid) using cardio-check.

Participants were instructed to fast overnight for 12 hours and diabetic patients on medication were requested to bring their medicine/insulin with them and take their medicine after providing the blood sample. To ensure high response rate for STEP3, the enumerators called the respondents on the day of testing if he/she failed to come as per the appointment.

[…]

Blood glucose and lipids: After STEP 1 and STEP 2 of data collection at sampled households, biochemical assessments were performed the next day at a designated place for each PSU for blood glucose and total cholesterol, measured in venous blood samples. Concentrations of glucose, total cholesterol and HDL cholesterol were measured in plasma samples. Fasting samples were taken to measure raised blood glucose.

Participants were instructed to fast overnight for 12 hours at the time of household visit for Step I and II. During the appointment, participant was asked to sit in comfortable position with exposing forearm in a table or if patient could not sit in that case at supine position. If the technician was not able to collect blood despite two attempts, he/she didn’t try to attempt the 3rd prick, and just recorded the reason for non-collection of sample in laboratory and interview tracking sheet. Each participant was given 50 taka and made him / her rest and then allowed to go).

The ante-cubital fossa was cleaned with disinfectant (70% alcohol) and identified the ante- cubital vein. Then 5ml of blood will be collected by disposable syringe. 2 ml of this blood was transferred to Fluoride-oxalate vacutainer (brown top) for serum glucose testing and 3 ml of the blood was kept in a normal tube and allowed to stand for separation of plasma (for lipid profile) with proper labeling. The sample for blood glucose was left in upright position in vacutainer rack and then centrifuged and separated serum was keep in the cold box (2–80C) surrounded by ice packs and sent to the NIPSOM Lab within 24 hours.

Each sample tube was labeled with the participant identification number using autogenerated ID tablet, as automatically generated during the questionnaire administration. The medical technologist labeled the laboratory ID (Based on PSU and HH number) against the corresponding participant ID on the appointment card following their lists.

Disposable sterile gloves in multiple sizes: The medical technologist and lab staffs used sterile gloves during blood collection from the participant. Each time the medical technologist washed his/her hands including gloves with Chlorhexidine Gluconate (0.5%) and Isopropyl alcohol (70%) (Hexisol®) and collect blood sample with sterile syringes and needles. A single-use disposable needles, and syringes or lancing devices were in sufficient numbers to ensure that each patient has a sterile needle and collection device or equivalent for each blood sampling. All the used syringes and all other used materials were collected in a supplied biohazard bag. The needles were stored in hard plastic container/box. All the medical wastes created for sample collections were sent within biohazard bag to NIPSOM. Finally all the medical wastes were disposed centrally scientifically by PRISM14, the specific agency concerned with management of biomedical wastes. NIPSOM had an agreement with PRISM for management of all laboratory and biomedical wastes. Sufficient laboratory sample tubes were supplied to prevent reuse and manual washing.

Immediately after reaching to the NIPSOM laboratory, the samples were properly registered with lab ID and sent for measuring blood glucose, lipid profile with biochemistry auto analyzer (Selectrao Pro M) for blood glucose, Human®, Germany; for HDL with control, Elitech®; TG, Elitech®; with control, Humatrol/ serodos®; cholesterol, Elitech® with control Humatrol®, Germany.“

*Source: National STEPS Survey for Non-communicable Diseases Risk Factors in Bangladesh 2018. Available at:* [*https://apps.who.int/iris/handle/10665/332886*](https://apps.who.int/iris/handle/10665/332886)

**Belarus: STEPS 2016**

“Biochemical studies were performed to determine the level of blood glucose, total cholesterol and high density lipoprotein (HDL). The concentration of glucose, total cholesterol and HDL was measured in fasting capillary blood of all respondents who signed an informed consent using the CardioCheck PA analyzer. Based on the results of laboratory studies, the respondents were divided into groups, taking into account the assumptions indicated in Table 3.7.”

*Source (translated via google translate from):
Prevalence of Risk Factors of Non-Communicable Diseases in the Republic of Belarus, STEPS 2016. Available at:* [*https://extranet.who.int/ncdsmicrodata/index.php/catalog/100/related_materials*](https://extranet.who.int/ncdsmicrodata/index.php/catalog/100/related_materials)

**Benin: STEPS 2015**

“Equipment:

Electronic device for the determination of capillary glycemia and total cholesterol + Triglycerides brand CardioChek ® P.A with MEMO chips and suitable strips

[…] The actual survey took place from October 19 to December 30, 2015.

During data collection, the first day was devoted to administering the questionnaire and taking physical measurements. An appointment was then made for the next morning at the respondent's home or sometimes in the nearest care unit or another appropriate place for fasting blood sugar and cholesterol tests.

[…] The morning of the second day was first devoted to fasting blood sugar and cholesterol tests by the team's qualified health worker (Laboratory Technician or Nurse) who pricked the participants’ fingers with a single use, sterile needle, and then took a small drop of blood to do the tests in front of the participant and give the results on the spot. The rest of this day and the third are devoted to revisits, then the cycle begins again.”

*Source: Benin Ministry of Health, Programme National de Lutte contre
les Maladies Non Transmissibles (2016). Rapport final de l’enquête pour la surveillance des facteurs de risque des maladies non transmissibles par l'approche ‘’STEPSwise’’ de l'OMS ENQUETE ‘’STEPS 2015’’ au Bénin. Available at:* [*https://www.who.int/ncds/surveillance/steps/benin/en/*](https://www.who.int/ncds/surveillance/steps/benin/en/)

**Bhutan: STEPS 2014**

“Immediately after the training, survey teams were allocated to the chiwog/enumeration areas where they would go to conduct the survey. Each team administered the STEP 1 (Questionnaire) and STEP 2 (Physical measurements) on the first visit to a household. The participants were then asked to fast overnight i.e. not consume any food or drinks (except water) after 10 p.m. the previous night until the blood sample was collected in the morning. A container was provided to collect urine samples prior to the beginning of the fast. Participants were asked to go to the testing centre set up by the survey team (located in the vicinity) the next morning. Here the blood samples were taken and the urine samples delivered to the survey team. Urine samples were sent by the survey team to the Jigme Dorji Wangchuk National Referral Hospital Laboratory (JDWNRH) in Thimphu for analysis of sodium and creatinine to determine mean population salt intake.“

*Source: National survey for noncommunicable disease risk factors and mental health using approach WHO Steps Approach in Bhutan – 2014 Available at:*

*https://www.who.int/ncds/surveillance/steps/Bhutan_2014_STEPS_Report.pdf*

**Botswana: STEPS 2014**

No further information available

**Burkina Faso: STEPS 2013**

“Step 3: the third step consisted of measuring blood glucose and blood cholesterol in capillary blood.

[...] Collection Equipment:

- Cardiocheck

[...] Operating difficulties were also encountered with blood sampling equipment, in this case cardiochecks, due to the ambient temperature which is often very high in places. The collection period coincided with a relatively warm climate and this often delayed data collection while waiting to find a spare cardiocheck.“

*Source, translated from: Report of the national survey for the prevalence of the main risk factors of noncommunicable disease in Burkina Faso 2013. Available at:*

*https://www.who.int/ncds/surveillance/steps/burkina_faso/en/*

**Chile: NHS 2009-10**

“The centralization of processing the samples reduced the variability of the laboratory analysis and allowed for a better monitoring of the quality of the processes. For this reason, it has been decided that all samples should be analysed by the Central Laboratory of the PUC, despite complex logistics and an increase in costs.

The Central Laboratory of the PUC has its own internal and external quality controls and it is sufficiently accredited. (Chilean norm 2.547: ISO 15.189). The preparation of the samples was undertaken in regional laboratories of the national health system SNSS which was monitored via interviews on the phone, field visits and a defined set of control indicators (e.g. number and type of alicuotes stored, average duration of centrifugation and monitoring of the temperature in freezers).

[...] The samples were divided into three aliquotes of 300ul in the regional laboratories. The aliquotes were stored at -20°C until being sent to the ISP (Chilean Public Health Institute) in where they were frozen at -80°C.

[...] This questionnaire was administered by a nurse in the household of the participant during a second visit. Here, the nurse collected the blood and urine sample, measured blood pressure, weight, hip, waist, and throat circumference and conducted a haemoglobin test.

[...] Total cholesterol: Enzymatic CHOD-PAP method

HDL colorimetric cholesterol (homogenous HDL)

LDL calculated with the Friedewald formula

Encymatic triglycerides with white glycerol

Encymatic glicemia (Hexokinase glucose)

[...] Number of samples:

Total cholesterol 2804

HDL cholesterol 2802

LDL cholesterol 2794

Triglyderides 2804

VLDL cholesterol 2794

[...] Lipds were measured for 55% of the study population.“

*Source, translated from: Resumen Ejecutivo: Encuesta Nacional de Salud ENS Chile 2009-10. Available at: http://epi.minsal.cl/encuesta-ens-anteriores/.*

**Costa Rica: STEPS 2010**

No further information available

**Ecuador: STEPS 2018**

“Each STEP 3 team included a health professional, liaison, and driver with their respective vehicle.

[…]

The activities of the health personnel were to implement STEP 3, that is, to determine the level of glucose and total cholesterol in capillary venous blood, using the portable glucose and cholesterol measurement equipment.

The STEPS 1 and 2 team applied the questionnaire on day 1, and the STEP 3 team took the biological samples on day 2, based on appointments made by the field team.”

*Source, translated from: Encuesta STEPS Ecuador 2018. Available at: https://extranet.who.int/ncdsmicrodata/index.php/catalog/774*

“Cardiochek devices were used for the blood measures in Step 3.“

*Source: NCD Microdata repository: Ecuador – STEPS 2018. Available at: https://extranet.who.int/ncdsmicrodata/index.php/catalog/774/study-description#page=data_collection&tab=study-desc*

**Eswatini: STEPS 2014**

“Fasting blood glucose and total cholesterol comprised the targeted biochemical measures of health risks for NCDs. On the first day of the survey after completion of STEP 1 and STEP 2, participants were asked to fast overnight of that day. i.e. people were asked not to consume any food except for clear water after taking dinner on that night until the survey team came in the morning of the following day (day 2). People in the selected EA were seen in their various homesteads where a finger prick was done using a CardioChek PA test system and a drop of blood was tested for glucose and total cholesterol. Those that complied with advice (fasting overnight) were eligible for testing.”

*Source: WHO STEPS: Noncommunicable Disease Risk Factor Surveillance Report Swaziland 2014. Available at:*

[*https://www.who.int/ncds/surveillance/steps/Swaziland_2014_STEPS_Report.pdf*](https://www.who.int/ncds/surveillance/steps/Swaziland_2014_STEPS_Report.pdf)

**Guyana: STEPS 2016**

“[...] Guyana [...] drew venous blood and did the blood analysis at the laboratory.

Guyana's Laboratory at which the blood testing was done is the Central Medical Laboratory, Georgetown, Guyana.”

*Source: Email contact with country team from the Caribbean Public Health Agency (CARPHA). Country Report forthcoming*

**Iran: STEPS 2016**

“Laboratory measurement and Bio–banking

We aimed to store the biological samples that will be randomly collected from all provinces (city/village) of Iran. Using the auto analyzer (Cobas C311 Hitachi High–Technologies Corporation. Tokyo Made in Japan) approved by Health Reference Laboratory, the levels of Total Cholesterol, Glucose, HDL–C, ALT, and Triglyceride were assessed from the plasma. […]

Temperature and transport requirements

Maintaining the optimal conditions for the transfer of biological samples using the updated standards for promotion of quality of biological samples and biomolecules maintenance, we developed the comprehensive participatory protocol and related instructions.

Blood and urine sample collection and transport were performed obtained from tube containing lithium heparin (3 cc) and urine (6 cc), the gathered biological samples were transferred to the central processing/archiving laboratory of study in NCDRC. Through a detailed time-binding action plan, the processes from collection to the central processing/archiving lab were managed in the shortest time (less than 18 hours).

All samples were transported in vaccine transport boxes. During transportation, in each cold box, a digital thermometer recorded the temperature of environment of the biological sample. These enable us to keep biological samples from freezing/ thawing.

[…] The first and second steps of study have been run for all selected samples and the third step was considered for those who were 25≤years of age.”

*Source:* *Djalalinia S, Modirian M, Sheidaei A, Yoosefi M, Zokaiee H, Damirchilu B, Mahmoudi Z, Mahmoudi N, Hajipour MJ, Peykari N, Rezaei N, Haghshenas R, Mohammadi MH, Delavari A, Gouya MM, Naderimagham S, Kousha A, Moghisi A, Mahdavihezaveh AR, Abachizadeh K, Majdzadeh R, Sayyari AA, Malekzadeh R, Larijani B, Farzadfar F. Protocol design for large–scale cross–sectional studies of surveillance of risk factors of non–communicable diseases in Iran: STEPs 2016. Arch Iran Med. 2017; 608 – 616.*

**Iraq: STEPS 2015**

“Laboratory requirements were procured by the Public Health central lab and distributed to the labs under quality control where biochemical analysis was carried out.“

*Source: Noncommunicable Diseases Risk Factor STEPS Survey Iraq 2015. Available at:*

*https://www.who.int/ncds/surveillance/steps/iraq/en/*

**Kiribati: STEPS 2015/16**

“In general, the survey personnel obtained informed consent from survey participants, gave fasting instructions to those participating in STEP 3, and made appointment times for those who consented to participate in the survey. Survey personnel conducted STEP 1 (questionnaire) at home if the participant was willing; if not, it will follow STEPS 2 and 3, which was done at a central location in each village on the second (or third) morning.

[…] The survey included taking blood and urine samples. To measure fasting blood glucose and total cholesterol, participants fasted from 10:00pm the previous night until 7:00am the following morning. Capillary blood samples were drawn using the finger prick method; and the Cardiochek used to measure total cholesterol, HDL and glucose in samples.”

*Source: Kiribati NCD Risk Factors STEPS Report 2015-2016.*

*Available at:* [*https://www.who.int/ncds/surveillance/steps/kiribati/en/*](https://www.who.int/ncds/surveillance/steps/kiribati/en/)

**Kyrgyzstan: STEPS 2013**

No further information available

**Lebanon: STEPS 2017**

“After completing Steps 1 and 2, the participants were referred, on specific dates, to the pre- assigned PHC for blood withdrawal. The centrifuged blood and urine samples were collected from the different centers and sent to the central laboratory for biochemical measurements. The laboratory procedures are found in the implementation plan (Annex 1).“

*Source: Who Stepwise Approach For Non-Communicable Diseases Risk Factor Surveillance. Lebanon 2016-2017. Available at:*

[*https://extranet.who.int/ncdsmicrodata/index.php/catalog/410/related_materials*](https://extranet.who.int/ncdsmicrodata/index.php/catalog/410/related_materials)

**Marshall Islands: HYBRID 2017**

No further information available

**Moldova: STEPS 2013**

“Laboratory tests were performed for blood glucose, total cholesterol and HDL cholesterol. Concentrations of glucose, total cholesterol and HDL cholesterol were measured in capillary blood the next day after STEPS 1 and 2 of the data collection. Capillary blood tests were performed for all survey respondents using a CardioCheck PA Analyzer, after fasting.“

*Source: Prevalence of Noncommunicable Disease Risk Factors in the Republic Of Moldova STEPS 2013. Available at: https://www.who.int/ncds/surveillance/steps/moldova/en/*

**Mongolia: STEPS 2013**

“Laboratory Analysis – blood glucose, total cholesterol and triglycerides were measured in peripheral (capillary) blood at the data collection site using dry chemical methods, biochemical analysis and automated analyzer. Serum samples were collected to analyze LDL and HDL cholesterol and spot urine was collected to determine sodium and creatinine levels in urine.

[…] Randomly selected individuals aged 15-64 years old who were eligible to participate and agreed upon, and signed a consent form, were involved in the step-3, laboratory testing. A researcher who performed anthropometric measurements, and signed the survey card, checked if the participant was eligible, and selected for the step-3 laboratory analysis. For the STEP-3 laboratory analysis, one-third of the selected participants aged 25-64 years were recruited. Laboratory analysis included testing for blood glucose, cholesterol, triglycerides, high density lipoprotein (HDL), and low density lipoprotein (LDL). Laboratory tests for LDL and HDL in blood, as well as sodium and creatinine content in urine were performed and analyzed in “Gyals” LLC’s laboratory using biochemical automated analyzer.

**Dry chemical method:** Concentrations of glucose, cholesterol and triglycerides as the intermediate, secondary risk factors of NCDs, were measured in peripheral (capillary) blood at the data collection sites with dry chemical methods using multi-functional “Prima home test” diagnostic device. Prima Home Test Multicare-In Meter for Glucose/ Cholestrol/ Triglycerides Diagnostic device is equipped with 2 technologies: Amperometric with glucose electrodes strips and Reflectometric with cholesterol and triglycerides strips. This diagnostic kit is easy-to-use, very clean and hygienic because it has the strip ejector switch to avoid contact with the used strips. It has a memory capacity of 500 measurements with date and time and analyzes results within 30 seconds. Thus, the participants were informed about the test results directly, at the study sites.

The research team members of the STEP III or laboratory step were involved in researchers’ training on how to use the “Prima home test” diagnostic kit, methodology to collect peripheral (capillary) blood at the data collection sites and safety measures.

**Measuring procedures:** After the “Prima home test” portable diagnostic device is regulated properly, a small size of blood sample is collected from a finger tip of a survey participant, and applied to the “yellow area” of a test strip. Blood glucose, cholesterol and triglycerides levels can be determined directly from this test. After each test, a laboratory staff member accurately entered the test results into a hand held computer, prior to starting the next participant’s test.

The “Prima home test” portable diagnostic device has the capacity to measure within the following range:

- Glucose: 0.6-33.3 mmol/L
- Cholesterol: 3.3-10.2 mmol/L
- Triglycerides: 0.56 - 5.6 mmol/L

When the measurement result was lower than the measuring range of the device, the result was evaluated as “very low”, and if the result was higher than the measuring range of the device, then the result was evaluated as “very high”. For instance, the measuring capacity for the lowest level of glucose is 0.6 mmol/L, therefore, the measurement results lower than this level was evaluated as “very low”. Similarly, if the glucose level was higher than 33.3 mmol/L, the highest level of the device’s measuring range, the result was evaluated as “very high”. The “very low” and “very high” measurement results were entered into computer programmes.

[…] High density lipoprotein (HDL) and Low density lipoprotein (LDL) content was measured in serum with an automatic analyzer using a direct or two-point linear method in 2,070 blood samples. Urine creatinine was determined using the Mindrayfafle method. A one-time (spot) test for sodium in the urine was determined using the electrolyte method in 2,058 urine samples, by “Gyals” LLC’s Laboratory.

The following requirements were complied with in blood and urine sample collection and transportation:

- Blood sample size to be not less than 2-3 ml
- Urine sample size to be not less than 8-10 ml
- Store samples in a special container in order to prevent hemolyzed specimens and clotted samples
- Samples to be stored at the temperature range of 2-8 0C
- Deliver blood and urine samples to the laboratory within one day in Ulaanbaatar, and

within three days in rural areas, complying with the required conditions for storage and

transportation

- Referral sheet for laboratory test samples must contain the survey participant’s age, sex,

the date when a sample was collected, and the date when a sample was delivered to the laboratory.

The following reagents and diagnostic kits were used for the laboratory tests:

1. For determining High density lipoprotein (HDL) content: HDL – Cholesterol - Kit manufactured by “Mindray” firm (Lot #142112023, Expiry date: May 2014)
2. For determining Low density lipoprotein (LDL) content: LDL – Cholesterol - Kit (Lot #142012017, Expiry date: May 2014)

[…] External and internal monitoring and evaluation were conducted on a regular basis in order to ensure the accuracy of, and compliance with, the standard requirements of the laboratory test results of the biochemical analysis. Regular internal quality control was conducted on a daily basis utilizing control serums “Multi control sera N” and “Multi control sera P” manufactured by the “Mindray” factory. In addition, the external independent quality control was conducted by the “Sysmex” corporation, where accuracy of the laboratory tests was monitored using “MEQAS for biochemistry” control samples prior to and during the biochemical analysis.

Gyals laboratory conducted the biochemical analysis during the period between May 14, 2013 and June 17, 2013 and handed over the test results coded by each survey participant to the PHI’s research team.“

*Source: Third national STEPS Survey on the Prevalence of Noncommunicable Disease and Injury Risk Factors-2013. Available at:*

*https://www.who.int/ncds/surveillance/steps/mongolia/en/*

**Morocco: STEPS 2017**

“During the second visit of the teams, Step3 was carried out by capillary blood test. The material used was Cardiochek® PA with a Chip MeMo, blood glucose Strips and blood lipids. The results were immediately given to the participants in results sheets. Each participant's barcodes were used to enter data on the tablets using the same code, allowing us to aggregate the data from the two runs. The recovered spots were packaged in the necessary conditions and sent daily to the reference laboratory by a specific transport company.”

*Source, translated from: National Noncommunicable Disease Risk Factor Survey Report 2017-20178. Available at: https://www.who.int/ncds/surveillance/steps/morocco/en/*

**Myanmar: STEPS 2014**

“Fasting blood glucose, 2 hour blood glucose, total blood cholesterol, triglycerides, HDL and LDL cholesterol were determined using SD LipidoCare Analyzer.

There were 18 data collection teams. In each data collection team, there were 6 members i.e. 1 team leader (medical doctor) for overall management and glucose loading and testing samples for blood glucose and lipids, 2 team members for face-face interview, 2 team members for measuring height and weight and 1 helper for registering and arranging participants.

A 5-day training workshop for the survey data collection teams was conducted at University of Medicine (2),Yangon on 11-15 August 2014

[…] The training workshop included sessions on the overview of STEPwise approach to NCD risk factor surveillance, the plan of the National Survey on Diabetes Mellitus and Risk Factors for Non-communicable Diseases in Myanmar, how to approach selected households and individuals including use of Kish method, PDA-based data collection, interview skills, informed consent, detailed discussion on the survey instrument and how to use show cards, mock interviews, demonstration and practice on physical measurements, use of SD LipiddoCare Analyzer for blood glucose and lipids, emergency management and referral of critically high blood glucose level for medical doctors in the data collection teams and quality control of all field processes.

Each team were provided with a field kit containing: […] devices and test strips for STEP 3 (plus lancets, swabs and sharp containers, gloves, pipettes) and glucose packs for oral glucose tolerance tests.

[…] On the day of the survey when STEP 1 and STEP 2 have been finished, participants were asked to fast overnight i.e. people were be asked not to consume any food or drinks after 10 p.m. at night, except water, until the morning of the following day.

Participants were asked to go to the designated testing centre the next morning where capillary blood was be taken by finger prick for rapid test. Those participants that complied with the fasting advice were eligible for blood sample collection. Blood glucose, cholesterol, triglycerides, HDL and LDL were measured using SD LipidoCare Analyzer onsite, which requires a finger-prick blood draw to measure glucose and blood lipids.“

*Source: Report on National Survey of Diabetes Mellitus and Risk Factors of Non-communicable Diseases in Myanmar (2014). Available at:*

*https://www.who.int/ncds/surveillance/steps/myanmar/en/*

**Seychelles: National Survey of Noncommunicable Diseases 2013**

“The following blood tests were performed within 2‐3 hours of blood collection at the clinical laboratory of the Seychelles Hospital: total cholesterol, HDL‐cholesterol, triglyceride, glucose, creatinine, uric acid, calcium, CRP (all these tests from 1 tube 84   with 1.5 ml serum), glucose (from yellow tube with sodium fluoride and potassium oxalate), A1c (violet EDTA tube). Insulin was analyzed 3 months later from one 1.5 ml microtube of serum.

• All analyses, except insulin and A1c, were done using a fully automatic analyzer Konelab 30i (Finland) with reagents from Thermo Fischer Scientific (USA).

• All tests performed using Konelab 30i were checked with controls on a daily basis.

[…] Cholesterol was measured after enzymatic hydrolysis by cholesterol esterase to cholesterol and free fatty acids, and free cholesterol oxidized by PEG cholesterol oxydase in peroxide, and then submitted to peroxidase to form a chromophore. Imprecision is <3.5% total CV, e.g. repeatability (within run) of 0.9% CV + within device (total) 1.4% CV at concentration of cholesterol of 5.2 mmol/l.

HDL‐cholesterol was measured with an enzymatic colorimetric test after precipitation of non HDL lipoproteins. The cholesterol concentration of HDL‐C is determined enzymatically similary as cholesterol (above). Imprecision is <4% of total CV, e.g. 0.5% CV within run + 1.1% CV between run + 1.6% CV between day; total % CV 2.0 at HDL concentration of 1.26 mmol/l.

Triglycerides were measured by hydrolysis by lipase (LPL) to glycerol and fatty acids, glycerol is phosphorylated to glycerol‐3‐ phosphate (by GK), then oxidized to hydrogen peroxide (GPO and POD) to form a quinoneime dye. Imprecision is <4% of total CV, e.g. 0.7% CV repeatability (within run) and CV 2.0% within device (total) at a concentration of triglyceride of 2.07 mmol/l.”

*Source: National Survey of Noncommunicable Diseases in Seychelles 2013‐2014 (Seychelles Heart Study IV): methods and main findings. Available at:*

*https://www.who.int/ncds/surveillance/steps/Seychelles_2013_STEPS_Report.pdf*

**Solomon Islands: STEPS 2015**

**“**Cardiochek was used for blood glucose and cholesterol measurements.“

*Source: NCD Microdata Repository: Solomon Islands – STEPS 2015. Available at:*

*https://extranet.who.int/ncdsmicrodata/index.php/catalog/710/study-description#page=data_collection&tab=study-desc*

**Sri Lanka: STEPS 2014/15**

**“**Cardiochek devices used for glucose and cholesterol measurements.“

*Source: NCD Microdata Repository: Sri Lanka – STEPS 2014. Available at:*

*https://extranet.who.int/ncdsmicrodata/index.php/catalog/614/study-description#page=data_collection&tab=study-desc*

**St. Vincent & the Grenadines: STEPS 2013**

“Fifty percent (50%) of the survey participants were asked to provide a biological specimen (finger prick) for Glucose and cholesterol testing using Glucose and Lipid Sampling Kits and respond to the nutrition intake (24 hour recall). The biological sample was only collected with participants’ explicit consent; the samples were not stored or used for additional undetermined or undisclosed future testing to which respondents did not agree at the time of participation.“

*Source: Ministry of Health, Wellness & the Environment (2015). National Health & Nutrition Survey – Non-Communicable Disease Risk Factor Surveillance Report. Kingstown, Saint Vincent and the Grenadines. Available at:*

*https://www.who.int/ncds/surveillance/steps/StVincent_STEPS_Report_2013-14.pdf?ua=1*

**Sudan: STEPS 2015**

“Blood samples were collected from those who complied with fasting advice and had given their informed consent. Blood glucose and cholesterol were measured using cardio-check examination equipment (Cardio check P.A. In vitro diagnostic medical devices for use with PTS panels test strips. Manufacturer: Polymer Technology Systems, INC, Indianapolis, IN USA CE 0197).”

*Source: Sudan STEPwise Survey for Non-communicable Diseases Risk Factors 2016 Report. Available at: https://www.who.int/ncds/surveillance/steps/sudan/en/*

**Tajikistan: STEPS 2016**

No further information available

**Timor-Leste: STEPS 2014**

“STEP 3 included biochemical measurements including fasting blood glucose and cholesterol were done by dry chemistry method using CardioCheck devices (Figure 2.5). All the measurements were taken at the house of the participant. […]

Day 1 – Survey of the suco for verification of the number of households, calculation of sampling interval, approaching households, taking consent from the selected individuals, interviewing for the STEP 1 and STEP 2, informing the respondents to fast for next day. Day 2 and 3 – Morning fasting samples to be taken from the respondents (by trained enumerators only) whose interviews were completed the previous day. The remaining respondents were interviewed and asked to be fasting for the next day to collect blood samples. The data collection effectively took 45 days “

*Source: Timor-Leste STEPS Survey Report, [online]*

*Available at http://www.who.int/entity/chp/steps/Timor-Leste_2014_STEPS_Report.pdf?ua=1*

**Tonga: STEPS 2017**

“Cardiochek PA devices were used for the blood measures in Step 3.”

*Source: NCD Microdata repository: Tonga – STEPS 2017. Available at:*

[*https://extranet.who.int/ncdsmicrodata/index.php/catalog/713/study-description#page=data_collection&tab=study-desc*](https://extranet.who.int/ncdsmicrodata/index.php/catalog/713/study-description#page=data_collection&tab=study-desc)

**Tokelau: STEPS 2014**

“Targeted biochemical measures of health risks for NCDs were measured including fasting blood glucose and total cholesterol. Selected core survey personnel were trained in conducting these measurements through the use of specific protocols with monitored quality control.

Each participant was provided with an appointment sheet and fasting instructions and also informed that there was refreshment prepared for them at the survey site so that they can have something to eat after measurement of their fasting blood glucose and total cholesterol.

As indicated in the diagram for the set-up of the venue, biochemical measurements were conducted in Station 2 after they had registered. Participants were instructed to fast from 10:00pm the previous night and scheduled for 07:00am the following morning for biochemical measurements with refreshment ready by the time the completed this station so that participants had something to eat before continuing on to the next STEPS of the survey. The survey team as well had an early morning each day of the survey, at about 6:00am to ensure that all stations were ready before the first participant registered.

Fasting blood glucose was measured using the Advantage glucose meter with test strips and capillary blood samples using finger pricks. The blood sampling and measure of fasting blood glucose followed specific control testing protocols, using the Accutrend Glucose control solution at the beginning of the day and after testing of about 20-25 patients or when there was an unusual participant result.

With each finger prick, two blood samples were obtained, one for the fasting blood glucose, and one for the blood cholesterol measurement. Total cholesterol was measured using the Accutrend GC meter and approporiate cholesterol test strips. The total cholesterol was measured once in mmol/Litre. Also, the meter was calibrated for accuracy at the beginning of the day, after about 20-25 participants, or when there was an unusual participant result. Accutrend Cholesterol control solution was used in this calibration.

After completion of these biochemical measurements, participants were directed towards the refreshment station, however, a few preferred to continue on to the blood pressure station before refreshment.”

*Source: Tokelau NCD Risk Factors STEPS Report. Available at: https://www.who.int/ncds/surveillance/steps/STEPS_Report_Tokelau.pdf*

**Tuvalu: STEPS 2015**

“Accutrend Plus meter and Accu-chek Performa were used for cholesterol and glucose measurements.“

Source: *NCD Microdata repository:Tuvalu – STEPS 2015. Available at:* [*https://extranet.who.int/ncdsmicrodata/index.php/catalog/639/study-description#page=data_collection&tab=study-desc*](https://extranet.who.int/ncdsmicrodata/index.php/catalog/639/study-description#page=data_collection&tab=study-desc)

**Vietnam: STEPS 2015**

“Devices for testing blood glucose and cholesterol (Cardio Check).

[…] STEPS 2 and 3: conducted by provincial preventive medicine centers under the supervision from National and Regional Epidemiology/Pasteur Institutes at selected Commune Health Station (CHS).

[…] - Finger blood test was used to measure blood glucose, total cholesterol and HDL

[…] Each province had one data collection team including 5 GATS interviewers who were in charge of interviewing at households and 3 local staff who were in charge of conducting STEPS 2 and 3 at the CHS. In each EA, the data collection was carried out in 2 days.

- The first day: Interview at household

5 GATS interviewers visited households in the provided list. A the households interviewer do the following:

[…]

- Instruct subject for overnight fasting and visiting the CHS in the next morning for physical measurement and blood test.
- In case the STEPS 2-3 cannot be done the next day, then the team in charge will inform

respondents of a suitable nearest date and then visited the households the day before to pass on the tube for urine sample collection and provided instruction for the respondents to fast and come to the CHS the day after for STEPS 2-3 data collection.

- The STEPS1 Coordinator then provided the interviewee list to STEPS 2-3 team coordinator for follow up and for STEPS 2-3 data collection.

• The second day: Physical measurement and blood tests at commune health station

In the morning when STEPS 2-3 data collection took place, village health collaborators went to households to invite subjects to bring urine tube to the CHS and participate in physical measurements and blood tests. The data collection was conducted in the early morning to ensure the fasting of subjects.

At the CHS, there was 3 staff to collect data:

- 01 technician to perform blood test using handheld devices and collects urine tube to store in the cold vacuum.
- 01 staff to perform blood pressure measurement following standard procedures.
- 01 staff to measure height, weight, waist circumference, and make conclusion.”

*Source: Vietnam Ministry of Health, General Department of Preventive Medicine (2016): National Survey on the Risk Factors of Non-communicable Diseases (STEPS) Vietnam, 2015. Hanoi. Available at:*

[*https://www.who.int/ncds/surveillance/steps/viet_nam/en/*](https://www.who.int/ncds/surveillance/steps/viet_nam/en/)

**Zambia: STEPS 2017**

“STEP 3 included blood chemistry rapid diagnostic tests to assess fasting blood glucose and total cholesterol. This was done by the use of Cardio-Check spot testing equipment.

[…] STEP 3 was done in the morning of the following day in most cases. However in some places modifications were made so that participants were prepared beforehand through local leaders and community health workers, who were contact before the research teams arrived. Eligible members from sampled families were asked to come to a central location on a named day and time. Participants were told not to eat until they were seen by the research team. Once on site, the research team explained the purpose of the study to sampled families. Prior to entering the names of those eligible, selection was done and urine was collected immediately after validating that it wasn’t the first time to pass urine that morning. For those who had fasted, glucose measurements were taken the same day while those who had not fasted, glucose measurements were done the following morning.“

*Source: Republic of Zambia Ministry of Health: Zambia Steps for Noncommunicable Diseases Risk Factors. Available at: https://www.who.int/ncds/surveillance/steps/zambia/en*
