# Supplementary material for: Unmet need for hypercholesterolemia care in 35 low- and middle-income countries: A cross-sectional study of nationally representative surveys
Source: PLoS Med. 2021 Oct 25;18(10):e1003841. doi: 10.1371/journal.pmed.1003841 (PMC8575312; doi:10.1371/journal.pmed.1003841)
Supplement: S4 Text — (DOCX) [file pmed.1003841.s004.docx]

# S4 Text: Data Cleaning

We used total cholesterol directly as reported. We calculated LDL cholesterol (LDL-C) from total cholesterol (TC), triglycerides, and HDL cholesterol. Whenever LDL-C values were already calculated, we recalculated them to ensure consistency across countries. Hence, in the following we specify cleaning not only for TC and LDL-C, but also for HDL cholesterol and triglycerides.

**Plausible ranges**

Lower cut-off

Values below 3 mg/dL for total cholesterol (N=0), triglycerides (N=0), and HDL cholesterol (N=44) to missing. This is the most conservative exclusion of data, given the possible measurement ranges of the various point-of-care measurement devices used in the countries (see S3 Text) [1].

Upper cut-off

While it is difficult to define upper ranges for total cholesterol, as physiologically very large values can occur, we decided to apply an upper cut-off, primarily because point-of-care devices are not always well-equipped to reliably measure these [1,2]. We oriented ourselves on the upper cut-offs that can be reliably measured by point-of-care-devices as well as ranges indicated to us by in-country lab partners and decided on the following: values above 300 mg/dL for TC (N=170), 600 mg/dL for triglycerides (N=72), and 100 mg/dL for HDL cholesterol (N=48) were set to missing. For LDL-C, values were set to missing if triglycerides were higher than 400 mg/dL (N=131). We include a sensitivity cascade analysis (Figure E in S1 Fig), in which we do not impose an upper cut-off for TC. As can be seen this does not substantially alter the results.

LDL-C negative values:

After dropping values below 3 mg/dL for TC, triglycerides, and HDL cholesterol, 12 observations had negative calculated LDL-C values. Of these, all corresponding TC, triglycerides, and most HDL cholesterol values were individually biological plausible. Each of these observations was flagged and then their LDL-C value was set to missing.

**Definitions**

LDL cholesterol

All LDL-C values were derived by our team according to the Friedewald equation [3]:

LDL-C (mg/dL) = TC (mg/dL) – HDL (mg/dL) - TG (mg/dL) / 5

Triglycerides and HDL cholesterol were only utilized to derive LDL-C. While both triglycerides and HDL cholesterol have been established as independent risk factors for coronary heart disease, their relevance and efficacy as targets of therapy is still unclear [4].

High total cholesterol

An individual was classified as having high TC if their level was above 6.21 mmol/L (according to ATP III guidelines) or if they reported taking medication for high cholesterol [4]. If individuals had only a self-reported medication status and no TC measurement, they were excluded from the analysis. A sensitivity analysis that includes 1209 additional individuals with no biomarker measurement, for whom high TC is defined purely based on the respondent’s self-reported medication status can be found in Figure D S1 Fig.

High LDL cholesterol

An individual was classified as having high levels of LDL-C if their level was above 4.14 mmol/L (according to ATP III guidelines) or if they reported taking medication for high cholesterol. If individuals had only a self-reported medication status and no LDL-C measurement, they were excluded from the analysis.

**Skip Patterns**

Biomarkers were taken of all respondents, except for pregnant women.

The self-reported variables follow the specified skip pattern:

| Country | Ever tested | Ever told | Taking meds |
| --- | --- | --- | --- |
| Algeria, Azerbaijan, Bangladesh, Belarus, Benin, Bhutan, Botswana, Burkina Faso, Ecuador, Eswatini, Guyana, Iran, Iraq, Kiribati, Kyrgyzstan, Lebanon, Marshall Islands, Moldova, Mongolia, Morocco, Myanmar, Solomon Islands, Sri Lanka, Saint Vincent & the Grenadines, Tajikistan, Timor-Leste, Tokelau, Tonga, Tuvalu, Vietnam, Zambia | asked | skip | skip |
| Chile, Costa Rica | asked | asked | skip |
| Sudan | ? | ? | ? |
| Seychelles | asked | asked | asked |

The cascades analysis was based on the natural STEPS skip pattern of “ever tested – asked”, “ever told – skip”, “taking meds – skip”. This skip pattern is followed by the majority of countries (see Table 1a). This means that if respondents replied to the question about whether they have ever had their lipids measured with “0: No”, then the subsequent questions on whether they have been ever told their hypercholesterolemia diagnosis or are taking medication were not asked, leaving their values for these respondents missing. Instead they were simply assumed to all be equal to “0: No”, since somebody who has never been tested could not be diagnosed.

For the countries following the skip pattern, we made this assumption tangible by setting the missings due to skip pattern to “0: No”. Answers that were coded as “don’t know” were also recoded as “0: No”.

For the countries that did not follow the skip pattern, we still imposed it artificially for matter of consistency with the STEPS countries. That is, whenever respondents answered the lead question with “0: No”, the following (skip) questions were set to “0: No”. There were very few instances in which this actually changed the coding. In case of the diagnosis stage, 2 observations switched from yes to no in Seychelles; 16 observations switched from yes to no in Chile; 11 observations switched from yes to no in Belize; and 81 observations switched from yes to no in Costa Rica. For the medication question, 1 observation switched from yes to no in Seychelles; 11 observations switched from yes to no in Chile; 2 observations switched from yes to no in Belize; and 72 observations switched from yes to no in Costa Rica.

| Country | Lifestyle Advice |
| --- | --- |
| Algeria, Belarus, Benin, Bhutan, Botswana, Burkina Faso, Costa Rica, Eswatini, Guyana, Iran, Iraq, Kiribati, Kyrgyzstan, Lebanon, Moldova, Mongolia, Morocco, Myanmar, Seychelles, Solomon Islands, Sri Lanka, Saint Vincent & the Grenadines, Sudan, Tajikistan, Timor-Leste, Tokelau, Tonga, Tuvalu, Vietnam | asked |
| Azerbaijan, Bangladesh, Ecuador, Zambia | skipped |

In four countries, also the lifestyle advice questions followed a skip pattern. This skip pattern was introduced by the question of whether the respondent has visited a doctor or health professional in the time frame as specified in the lifestyle advice questions (see below for exact phrasing). If respondents said no, they would not be asked the lifestyle advice questions either. In order to be consistent with the procedure above, we recoded lifestyle advice in these cases to be “0: No”.

**Consistency of Phrasing Across Surveys**

Measurement

| Algeria, Azerbaijan, Bangladesh, Belarus, Benin, Bhutan, Botswana, Burkina Faso^*^, Ecuador, Eswatini, Guyana, Iran, Kiribati, Kyrgyzstan, Lebanon, Moldova, Morocco, Solomon Islands, Sri Lanka, Saint Vincent & the Grenadines, Tajikistan, Timor-Leste, Tokelau, Tonga, Tuvalu, Vietnam, Zambia | Have you ever had your cholesterol (fat levels in your blood) measured by a doctor or other health worker? |
| --- | --- |
| Chile | When was the last time you had your cholesterol measured |
| Costa Rica | In the last 12 months, has there been any lipid analysis (cholesterol, triglycerides, or fats) in the blood) |
| Marshall Islands | Blood cholesterol is a fatty substance found in the blood. Have you ever had your blood cholesterol checked by a doctor, nurse, or other health worker? |
| Mongolia, Myanmar | Have you ever had your cholesterol measured by a doctor or other health worker? |
| Seychelles | Have you ever had your blood cholesterol checked? |

Diagnosis

| Algeria, Azerbaijan, Belarus, Benin, Bhutan, Botswana, Burkina Faso^*^, Ecuador, Eswatini, Guyana, Iran, Kiribati, Kyrgyzstan, Lebanon, Moldova, Mongolia, Morocco, Myanmar, Solomon Islands, Sri Lanka, Saint Vincent & the Grenadines, Tajikistan, Timor-Leste, Tokelau, Tonga, Tuvalu, Vietnam, Zambia | Have you ever been told by a doctor or other health worker that you have raised cholesterol? |
| --- | --- |
| Chile | Has a doctor, nurse or other health professional ever told you that you have had or have high cholesterol? |
| Costa Rica | Have you been diagnosed with an alteration of lipids (cholesterol, triglycerides, or fats) by a doctor or other health professional |
| Marshall Islands | Have you ever been told by a doctor, nurse, or other health professional that your blood cholesterol is high? |
| Seychelles | Has a doctor, nurse or any other healthcare worker ever told you that you have high blood cholesterol |

Medication

| Belarus | In the past two weeks, have you taken any high cholesterol drugs (medication) prescribed by a doctor or other healthcare professional? |
| --- | --- |
| Algeria, Azerbaijan, Bangladesh, Benin, Bhutan, Botswana, Burkina Faso^*^, Ecuador, Eswatini, Guyana, Iran, Kiribati, Kyrgyzstan, Lebanon, Moldova, Mongolia, Morocco, Myanmar, Solomon Islands, Sri Lanka, Saint Vincent & the Grenadines, Tajikistan, Timor-Leste, Tokelau, Tonga, Tuvalu, Vietnam, Zambia | In the past two weeks, have you taken any oral treatment (medication) for raised total cholesterol prescribed by a doctor or other health worker? |
| Chile | Are you currently taking or doing some program, treatment or change in lifestyle to keep your cholesterol controlled? What kind of treatment are you taking? |
| Costa Rica | Do you currently receive any of the treatments or the advice indicated below, prescribed by a doctor or other health professional, for having alterations of the lipids (cholesterol, triglycerides, or fats)? Medication taken during the last two weeks |
| Marshall Islands | Are you currently receiving drugs medicine prescribed by a doctor or other health worker for your high cholesterol that you have taken in the past two weeks? |
| Seychelles | Do you currently take any medication to reduce your blood cholesterol (statin)? |

Advice

| Azerbaijan, Bangladesh, Ecuador | During any of your visits to a doctor or other health worker in the past 12 months, were you advised to do any of the following? Start or do more physical activity; maintain a healthy body weight or lose weight; reduce fat in your diet; quit using tobacco or don’t start; eat at least five servings of fruit and/or vegetables each day |
| --- | --- |
| Algeria, Belarus, Benin, Bhutan, Botswana, Burkina Faso^*^, Eswatini, Guyana, Iran, Kiribati, Kyrgyzstan, Lebanon, Moldova, Morocco, Myanmar, Solomon Islands, Sri Lanka, Saint Vincent & the Grenadines, Tajikistan, Timor-Leste, Tokelau, Tuvalu, Vietnam, Zambia | During the past three years, has a doctor or other health worker advised you to do any of the following? Start or do more physical activity; maintain a healthy body weight or lose weight; reduce fat in your diet; quit using tobacco or don’t start; eat at least five servings of fruit and/or vegetables each day |
| Chile, Marshall Islands | NA |
| Costa Rica | Do you currently receive any of the treatments or the advice indicated below, prescribed by a doctor or other health professional, for having alterations of the lipids (cholesterol, triglycerides, or fats)? Special diet by medical prescription; Advice or treatment to lose weight |
| Mongolia | During the past three years, has a doctor or other health worker advised you to do any of the following? Do at least 30 minutes of physical activity on at least 5 days per week; maintain a healthy body weight or lose weight; reduce fat in your diet; quit using tobacco or don’t start; eat at least five servings of fruit and/or vegetables each day |
| Seychelles | During the past 12 months, did a health officer advise you about smoking; your diet; in relation to weight control; about the need to have more regular physical activity, perhaps in relation to a medical condition that you may have? |
| Tonga | During the past three years, has a doctor or other health worker advised you to do any of the following? Start or do more physical activity; maintain a healthy body weight or lose weight; quit using tobacco or don’t start |

^*^ No survey instrument was available for Burkina Faso. However, from the survey report as well as the question labeling of the data, it appears that these questions were taken directly from the generic WHO STEPS questionnaire (unlike others relating to food hygiene and fruit and vegetable intake that were adapted to the local context).

As can be seen above, there are no substantive differences in the ever measured questions across countries, except for small deviations in the case of Costa Rica – which refers to past 12 months only – and Chile. This has to be taken into account when interpreting the cascades results of Costa Rica as we might be underestimating the met need for care at the measurement stage, if many diagnoses occurred earlier than one year prior to the survey. The diagnosis questions are also virtually the same throughout. In case of the medication phrasing slight differences in again the time frame of the question occurs. While most countries specify a time period of two weeks, others ask for current treatment. We assumed this did not affect the cascades analysis. The survey questions on lifestyle advice are again almost the same throughout, except for Chile and Marshall Islands, in which case we do not have data on this variable, and Tonga, which did not ask about diet advice received.

# References

1. pts Diagnostics. PTS Panels Test Strips and Controls. In: PTS Diagnostics [Internet]. [cited 29 Jul 2019]. Available: https://ptsdiagnostics.com/pts-panels-test-strips-and-controls/

2. Panz VR, Raal FJ, Paiker J, Immelman R, Miles H. Performance of the CardioChek PA and Cholestech LDX point-of-care analysers compared to clinical diagnostic laboratory methods for the measurement of lipids. 2005; 5.

3. Friedewald WT, Levy RI, Fredrickson DS. Estimation of the Concentration of Low-Density Lipoprotein Cholesterol in Plasma, Without Use of the Preparative Ultracentrifuge. Clinical Chemistry. 1972;18: 4.

4. National Cholesterol Education Program E. Detection, Evaluation, and Treatment of High Blood Cholesterol in Adults (Adult Treatment Panel III) - Final Report. National Institutes of Health; 2001. Report No.: No. 01-3305.
