# Supplementary material for: Unmet need for hypercholesterolemia care in 35 low- and middle-income countries: A cross-sectional study of nationally representative surveys
Source: PLoS Med. 2021 Oct 25;18(10):e1003841. doi: 10.1371/journal.pmed.1003841 (PMC8575312; doi:10.1371/journal.pmed.1003841)
Supplement: S5 Text — (DOCX) [file pmed.1003841.s005.docx]

# S5 Text: Mathematical Equation to Regression Specifications

We apply a modified Poisson regression specification to estimate the association between individual-level characteristics and cascade progression using the following framework:

${CascStage}_{ip}^{n}=\alpha+ \beta{IndCharac}_{i}+\delta_{c}+\varepsilon_{ip}$ if ${CascStage}_{is}^{n-1}=1$,

where

${CascStage}_{si}^{n}$is the binary outcome variable of individual $i$ in primary sampling unit $p$ describing whether the respective cascade stage $n$ was achieved conditional on completion of prior cascade stages. Specifically, the four cascade stages are “Lipids Measured”, “Aware of Diagnosis”, “Advice or Medication”, and “Controlled Disease”;

$\alpha$ is the intercept;

${IndCharac}_{i}$ is a vector with characteristics of individual *i*, i.e. age, sex, education, smoking, body mass index, diabetes status, and hypertension status;

$\delta_{c}$ is a fixed effect for the survey in country $c$; and
$\varepsilon_{ip}$ is an individual level error term with a robust structure, clustering at the primary sampling unit $p$.
