# Supplementary material for: Unmet need for hypercholesterolemia care in 35 low- and middle-income countries: A cross-sectional study of nationally representative surveys
Source: PLoS Med. 2021 Oct 25;18(10):e1003841. doi: 10.1371/journal.pmed.1003841 (PMC8575312; doi:10.1371/journal.pmed.1003841)
Supplement: S6 Text — (DOCX) [file pmed.1003841.s006.docx]

# S6 Text: STROBE Checklist

|  | **Item No** | **Recommendation** |
| --- | --- | --- |
| **Title and abstract** | 1 | (*a*) Indicate the study’s design with a commonly used term in the title or the abstract **The title includes this information.** |
|  |  | (*b*) Provide in the abstract an informative and balanced summary of what was done and what was found  **This information is included in the abstract.** |
| **Introduction** | | |
| Background/rationale | 2 | Explain the scientific background and rationale for the investigation being reported  **This information is contained throughout the introduction.** |
| Objectives | 3 | State specific objectives, including any prespecified hypotheses  **This information is provided in the final paragraph of the introduction.** |
| **Methods** | | |
| Study design | 4 | Present key elements of study design early in the paper  **This information is provided in the Methods section, in the subsection on Data Sources and Cascade Construction.** |
| Setting | 5 | Describe the setting, locations, and relevant dates, including periods of recruitment, exposure, follow-up, and data collection  **This information is provided in the Methods, in the subsection on Data Sources.** |
| Participants | 6 | (*a*) Give the eligibility criteria, and the sources and methods of selection of participants. Describe methods of follow-up  **This information is provided in the Methods section, in the subsection on Data Sources.** |
|  |  | (*b*) For matched studies, give matching criteria and number of exposed and unexposed  **We did not use a matched design.** |
| Variables | 7 | Clearly define all outcomes, exposures, predictors, potential confounders, and effect modifiers. Give diagnostic criteria, if applicable  **Please see Methods, subsections on Cascade Construction and Statistical Analysis.** |
| Data sources/ measurement | 8* | For each variable of interest, give sources of data and details of methods of assessment (measurement). Describe comparability of assessment methods if there is more than one group  **This information is contained in the Methods section, under the subheadings Data Sources and Cascade Construction.** |
| Bias | 9 | Describe any efforts to address potential sources of bias  **This information is provided in the Methods section, under the subheading Cascade Construction and Statistical Analyses.** |
| Study size | 10 | Explain how the study size was arrived at  **This information can be found in Methods, under the section on Data Sources.** |
| Quantitative variables | 11 | Explain how quantitative variables were handled in the analyses. If applicable, describe which groupings were chosen and why  **Please see the Methods section for this information, under the subheadings Data Sources, Cascade Construction, and Statistical Analysis.** |
| Statistical methods | 12 | (*a*) Describe all statistical methods, including those used to control for confounding **Please see Methods, in the subsection Statistical Analyses.** |
|  |  | (*b*) Describe any methods used to examine subgroups and interactions  **Please see Methods, in the subsection Statistical Analyses.** |
|  |  | (*c*) Explain how missing data were addressed  **Please see Methods, in the subsection Cascade Construction** |
|  |  | (*d*) If applicable, explain how loss to follow-up was addressed  **This is not applicable as this study did not have loss to follow-up.** |
|  |  | (*e*) Describe any sensitivity analyses  **This information is provided in the Methods section under the subheading Statistical Analysis.** |
| **Results** | | |
| Participants | 13* | (a) Report numbers of individuals at each stage of study—eg numbers potentially eligible, examined for eligibility, confirmed eligible, included in the study, completing follow-up, and analysed  **Please see S1 Text for details on the survey search process.** |
|  |  | (b) Give reasons for non-participation at each stage  **Please see S1 Text for details on the survey search process including reasons for exclusion.** |
|  |  | (c) Consider use of a flow diagram  **We have not used a flow diagram.** |
| Descriptive data | 14* | (a) Give characteristics of study participants (eg demographic, clinical, social) and information on exposures and potential confounders  **This information is provided in Table 1 of the main manuscript.** |
|  |  | (b) Indicate number of participants with missing data for each variable of interest **This information is provided in S3 Table.** |
|  |  | (c) Summarise follow-up time (eg, average and total amount)  **The survey years are provided in the Data Sources Section and Table A in S1 Table. There are no formal follow-up times in this study.** |
| Outcome data | 15* | Report numbers of outcome events or summary measures over time  **This information is provided in Results Section, subsection Sample Characteristics, and Table 1 of the main manuscript.** |
| Main results | 16 | (*a*) Give unadjusted estimates and, if applicable, confounder-adjusted estimates and their precision (eg, 95% confidence interval). Make clear which confounders were adjusted for and why they were included  **This information is provided in Table 2 and Figures 1-2 of the main manuscript.** |
|  |  | (*b*) Report category boundaries when continuous variables were categorized  **These are reported in Table 2 of the main manuscript.** |
|  |  | (*c*) If relevant, consider translating estimates of relative risk into absolute risk for a meaningful time period  **This is not applicable to this study.** |
| Other analyses | 17 | Report other analyses done—eg analyses of subgroups and interactions, and sensitivity analyses  **These results are reported in S2 Table and S1 Fig.** |
| **Discussion** | | |
| Key results | 18 | Summarise key results with reference to study objectives  **This information can be found in paragraphs 1-6 of the Discussion section.** |
| Limitations | 19 | Discuss limitations of the study, taking into account sources of potential bias or imprecision. Discuss both direction and magnitude of any potential bias  **This information can be found in paragraph 7 of the Discussion section.** |
| Interpretation | 20 | Give a cautious overall interpretation of results considering objectives, limitations, multiplicity of analyses, results from similar studies, and other relevant evidence  **This information can be found in the Discussion section, paragraphs 2-6.** |
| Generalisability | 21 | Discuss the generalisability (external validity) of the study results  **This information can be found in the Discussion, in paragraph 2-6 of the manuscript**. |
| **Other information** | | |
| Funding | 22 | Give the source of funding and the role of the funders for the present study and, if applicable, for the original study on which the present article is based  **We have provided this information in the section titled “Funding” following the manuscript.** |
