# Supplementary material for: Unmet need for hypercholesterolemia care in 35 low- and middle-income countries: A cross-sectional study of nationally representative surveys
Source: PLoS Med. 2021 Oct 25;18(10):e1003841. doi: 10.1371/journal.pmed.1003841 (PMC8575312; doi:10.1371/journal.pmed.1003841)
Supplement: S1 Table — (DOCX) [file pmed.1003841.s007.docx]

# S1 Table: Sample Characteristics

Table A: Summary of 35 population-based surveys conducted in low- and middle-income countries and country-level characteristics

Table B: Socio-demographic sample characteristics for respondents with TC and LDL-C in normal ranges and no self-reported medication use

|  | **Total Cholesterol Sample*** | | **LDL Cholesterol Sample**** | |  |
| --- | --- | --- | --- | --- | --- |
|  | Normal TC | | Normal LDL-C | |  |
|  | Number of Observations† | Percentage or Mean‡ | Number of Observations† | Percentage or Mean‡ |  |
| Female | 118264 | 50 | 52016 | 52 |  |
| Age(mean) | 118265 | 39 | 52017 | 40 |  |
| 15 - 24 y/o | 12096 | 16 | 12202 | 13 |  |
| 25 - 34 y/o | 28735 | 27 | 29043 | 27 |  |
| 35 - 44 y/o | 28732 | 23 | 29421 | 24 |  |
| 45 - 54 y/o | 23997 | 18 | 25275 | 18 |  |
| 55 - 64 y/o | 17429 | 12 | 18915 | 12 |  |
| 65+ y/o | 7314 | 5 | 7869 | 5 |  |
| Education |  |  |  |  |  |
| Less than primary school | 23660 | 21 | 24110 | 26 |  |
| Less than secondary school | 35936 | 34 | 37030 | 39 |  |
| Secondary completed or higher | 57022 | 45 | 59794 | 36 |  |
| BMI |  |  |  |  |  |
| Normal | 51219 | 53 | 52385 | 49 |  |
| Underweight | 8092 | 10 | 8210 | 9 |  |
| Overweight | 32648 | 25 | 34053 | 27 |  |
| Obese | 24309 | 13 | 25956 | 15 |  |
| Smoking# | 117630 | 20 | 51682 | 20 |  |
| Diabetic | 111825 | 7 | 51098 | 8 |  |
| Hypertensive | 117105 | 25 | 51501 | 25 |  |
| Screening recommended§ | 118265 | 66 | 52017 | 69 |  |
| ^*^ Includes respondents from all 32 countries with a valid total cholesterol measurement ^**^ Includes respondents from Algeria, Bangladesh, Burkina Faso, Chile, Costa Rica, Iran, Iraq,  Lebanon, Mongolia, Morocco, Myanmar, Seychelles, and St. Vincent & the Grenadines with a valid  LDL-C measurement ^†^ Unweighted ^‡^ Values account for sampling design with survey weights re-scaled by the survey’s sample size such  that all countries contribute to estimates according to their population size ^#^ Respondents that are currently smoking or were smoking within past 12 months are classified as   smoking (as per WHO PEN Protocol 1) ^§^ According to the PEN protocol, screening is recommended whenever the respondent exhibits at least   one of the following risk factors: age >= 40; smoking; diabetic; hypertensive; waist circumference  >= 90 in males; waist circumference >=100 in females | | | | |  |
|  |  |  |  |  |  |
|  |  |  |  |  |  |
|  |  |  |  |  |  |
|  |  |  |  |  |  |
|  |  |  |  |  |  |
|  |  |  |  |  |  |
|  |  |  |  |  |  |
|  |  |  |  |  |  |
|  |  |  |  |  |  |
|  |  |  |  |  |  |

Table C: High TC and High LDL-C Prevalences by Age Cohort

|  | Total Cholesterol Sample* | | LDL Cholesterol Sample** | |  |
| --- | --- | --- | --- | --- | --- |
|  | Prevalence | Confidence Interval | Prevalence | Confidence Interval |  |
| Overall | 7.1 | [6.8 , 7.4] | 7.5 | [7.1 , 7.9] |  |
| By Age |  |  |  |  |  |
| 15 - 24 y/o | 1.6 | [1.2 , 2.2] | 2.0 | [1.4 , 2.8] |  |
| 25 - 34 y/o | 3.4 | [2.9 , 4.1] | 3.8 | [3.1 , 4.5] |  |
| 35 - 44 y/o | 5.8 | [5.3 , 6.3] | 6.1 | [5.5 , 6.7] |  |
| 45 - 54 y/o | 10.8 | [10 , 11.6] | 10.6 | [9.6 , 11.7] |  |
| 55 - 64 y/o | 14.4 | [13.3 , 15.5] | 14.2 | [12.9 , 15.6] |  |
| 65+ y/o | 14.8 | [13.2 , 16.6] | 16.3 | [13.6 , 19.4] |  |
| Note: Prevalences account for sampling design with survey weights re-scaled by the survey’s sample size such that all countries contribute to estimates according to their population size. * Includes respondents from all 32 countries with a valid total cholesterol measurement;  ** Includes respondents from Algeria, Bangladesh, Burkina Faso, Chile, Costa Rica, Iran, Iraq, Lebanon, Mongolia, Morocco, Myanmar, Seychelles, and St. Vincent & the Grenadines with a valid LDL-C measurement | | | | |  |
|  |  |  |  |  |  |
|  |  |  |  |  |  |
|  |  |  |  |  |  |
|  |  |  |  |  |  |
|  |  |  |  |  |  |
|  |  |  |  |  |  |
|  |  |  |  |  |  |
|  |  |  |  |  |  |
