# Supplementary material for: Unmet need for hypercholesterolemia care in 35 low- and middle-income countries: A cross-sectional study of nationally representative surveys
Source: PLoS Med. 2021 Oct 25;18(10):e1003841. doi: 10.1371/journal.pmed.1003841 (PMC8575312; doi:10.1371/journal.pmed.1003841)
Supplement: S2 Table — (DOCX) [file pmed.1003841.s008.docx]

# S2 Table: Supplementary Analysis

Table A: Cascade of Care Disaggregated for Medication and Lifestyle Advice Stage

|  | High TC | | High LDL-C | |
| --- | --- | --- | --- | --- |
|  | Percent | 95% CI | Percent | 95% CI |
| Hypercholesterolemia | 100 |  | 100 |  |
| Lipids Measured | 43 | [40 , 45] | 47 | [44 , 50] |
| Aware of Diagnosis | 31 | [29 , 33] | 36 | [33 , 38] |
| Medication | 24 | [22 , 26] | 29 | [27 , 32] |
| Controlled Disease | 7 | [6 , 8] | 19 | [18 , 21] |
| Advice | 25 | [23 , 27] | 26 | [24 , 28] |
| Controlled Disease | 6 | [5 , 7] | 15 | [14 , 17] |
| Only Medication | 5 | [3 , 7] | 7 | [6 , 8] |
| Controlled Disease | 1 | [1 , 2] | 4 | [3 , 5] |
| Only Advice | 5 | [4 , 6] | 4 | [3 , 5] |
| Controlled Disease | 0 | [0 , 0] | 0 | [0 , 0] |

Note: Hypercholesterolemia refers to all respondents that are classified as having high TC (≥240 mg/dL) or a self-reported medication status in the first column and high LDL-C (≥160 mg/dL) or a self-reported medication status in the second column. Medication refers to those that have received medication for their high cholesterol (independent of whether lifestyle advice was also received) and advice refers to those that have received lifestyle advice (independent of whether medication was also received). Only Medication refers to having received medication, but not lifestyle advice. Only advice refers to having received lifestyle advice, but not medication. Controlled Disease considers those respondents that have TC and LDL-C values within the range considered normal by ATP III guidelines. All cascade stages are restricted on reaching the prior cascade stages. All calculations incorporate Primary Sampling Units and strata to account for the different survey designs of included countries, as well as use sampling weights rescaled such that all countries contribute equally.

Table B: Cascade of Care by Region

|  | Number of Observations With Biomarker^*^ | Prevalence of Hypercholesterolemia^†^ | | Number of Observations With Hyper-cholesterolemia^**^ | Lipids Measured^†‡^ | | Aware of Diagnosis^†‡^ | | Advice or Medication^†‡^ | | Controlled Disease^†‡^ | |  |
| --- | --- | --- | --- | --- | --- | --- | --- | --- | --- | --- | --- | --- | --- |
|  |  | Percent | 95% CI |  | Percent | 95% CI | Percent | 95% CI | Percent | 95% CI | Percent | 95% CI |  |
| Africa |  |  |  |  |  |  |  |  |  |  |  |  |  |
| High TC | 26413 | 4.8 | [2.3 , 9.6] | 1198 | 29 | [21 , 40] | 22 | [15 , 30] | 20 | [15 , 28] | 7 | [4 , 13] |  |
| High LDL-C | 8560 | 9.8 | [4.1 , 21.6] | 756 | 41 | [31 , 52] | 27 | [22 , 33] | 25 | [21 , 29] | 15 | [13 , 18] |  |
| The Americas |  |  |  |  |  |  |  |  |  |  |  |  |  |
| High TC | 11154 | 15.1 | [13.5 , 16.9] | 1983 | 66 | [61 , 71] | 50 | [45 , 55] | 44 | [39 , 48] | 9 | [7 , 11] |  |
| High LDL-C | 5862 | 16.8 | [14.3 , 19.8] | 1310 | 63 | [57 , 70] | 49 | [42 , 55] | 42 | [35 , 49] | 16 | [13 , 20] |  |
| S.E. Asia & Western Pacific |  |  |  |  |  |  |  |  |  |  |  |  |  |
| High TC | 39765 | 6.3 | [5.7 , 7] | 2966 | 34 | [30 , 38] | 25 | [21 , 29] | 23 | [20 , 27] | 2 | [1 , 2] |  |
| High LDL-C | 15582 | 7.1 | [6 , 8.2] | 1204 | 15 | [12 , 19] | 10 | [8 , 13] | 10 | [8 , 13] | 6 | [5 , 8] |  |
| Eastern Mediterranean & Europe |  |  |  |  |  |  |  |  |  |  |  |  |  |
| High TC | 51708 | 8.5 | [8 , 9] | 4590 | 52 | [49 , 55] | 37 | [34 , 39] | 36 | [33 , 38] | 14 | [13 , 15] |  |
| High LDL-C | 28328 | 13.8 | [12.7 , 14.9] | 3045 | 63 | [59 , 66] | 51 | [48 , 55] | 51 | [47 , 54] | 35 | [32 , 38] |  |
| Note:  * Number of observations, aged 15+ and nonpregnant, with a valid measurement of TC in the case of High TC and LDL-C in the case of High LDL-C ** Number of all respondents classified as having dyslipidemia based on TC / LDL-C measures or a self-reported medication status (defined by exceeding ATP III guideline   cutoffs, ie.TC≥240 mg/dL / LDL-C≥160 mg/dL or respondent taking lipid medication) † Adjusted for sampling design ‡ See note Table A | | | | | | | | | | | | |  |
|  |  |  |  |  |  |  |  |  |  |  |  |  |  |
|  |  |  |  |  |  |  |  |  |  |  |  |  |  |
|  |  |  |  |  |  |  |  |  |  |  |  |  |  |
|  |  |  |  |  |  |  |  |  |  |  |  |  |  |

Table Table C: Cascade of Care by World Income Classification

|  | Number of Observations With Biomarker^*^ | Prevalence of Hypercholesterolemia^†^ | | Number of Observations With Hyper-cholesterolemia^**^ | Lipids Measured^†‡^ | | Aware of Diagnosis^†‡^ | | Advice or Medication^†‡^ | | Controlled Disease^†‡^ | |  |
| --- | --- | --- | --- | --- | --- | --- | --- | --- | --- | --- | --- | --- | --- |
|  |  | Percent | 95% CI |  | Percent | 95% CI | Percent | 95% CI | Percent | 95% CI | Percent | 95% CI |  |
| Low Income |  |  |  |  |  |  |  |  |  |  |  |  |  |
| High TC | 9201 | 2.2 | [1.8 , 2.6] | 256 | 13 | [6 , 24] | 8 | [3 , 20] | 8 | [3 , 20] | 0 | [0 , 0] |  |
| High LDL-C | 1374 | 1.1 | [0.6 , 1.9] | 16 | 9 | [1 , 58] | 0 | [0 , 0] | 0 | [0 , 0] | 0 | [0 , 0] |  |
| Lower-middle Income |  |  |  |  |  |  |  |  |  |  |  |  |  |
| High TC | 58668 | 4.3 | [3.9 , 4.7] | 3343 | 29 | [27 , 32] | 22 | [19 , 25] | 21 | [19 , 24] | 4 | [4 , 5] |  |
| High LDL-C | 20171 | 5.9 | [5.1 , 6.7] | 1347 | 28 | [25 , 32] | 22 | [19 , 25] | 21 | [19 , 25] | 17 | [14 , 20] |  |
| Upper-middle Income |  |  |  |  |  |  |  |  |  |  |  |  |  |
| High TC | 61171 | 11.9 | [10.8 , 13.2] | 7138 | 59 | [56 , 61] | 43 | [40 , 45] | 39 | [37 , 42] | 11 | [9 , 13] |  |
| High LDL-C | 36787 | 16.4 | [14.7 , 18.4] | 4952 | 61 | [57 , 65] | 47 | [43 , 51] | 43 | [38 , 48] | 23 | [21 , 25] |  |
| Note:  * Number of observations, aged 15+ and nonpregnant, with a valid measurement of TC in the case of High TC and LDL-C in the case of High LDL-C ** Number of all respondents classified as having dyslipidemia based on TC / LDL-C measures or a self-reported medication status (defined by exceeding ATP III guideline   cutoffs, ie.TC≥240 mg/dL / LDL-C≥160 mg/dL or respondent taking lipid medication) † Adjusted for sampling design ‡ See note Table A | | | | | | | | | | | | |  |
|  |  |  |  |  |  |  |  |  |  |  |  |  |  |
|  |  |  |  |  |  |  |  |  |  |  |  |  |  |
|  |  |  |  |  |  |  |  |  |  |  |  |  |  |
|  |  |  |  |  |  |  |  |  |  |  |  |  |  |

Table D: Cascade of Care by Country

|  | Number of Observations With Biomarker^*^ | Prevalence of Hypercholesterolemia^†^ | | Number of Observations With Hypercholesterolemia^**^ | Lipids Measured^†‡^ | | Aware of Diagnosis^†‡^ | | Advice or Medication^†‡^ | | Controlled Disease^†‡^ | |  |
| --- | --- | --- | --- | --- | --- | --- | --- | --- | --- | --- | --- | --- | --- |
|  |  | Percent | 95% CI |  | Percent | 95% CI | Percent | 95% CI | Percent | 95% CI | Percent | 95% CI |  |
| Algeria |  |  |  |  |  |  |  |  |  |  |  |  |  |
| High TC | 6132 | 6.1 | [5.4 , 6.9] | 459 | 63 | [57 , 69] | 49 | [44 , 54] | 47 | [42 , 52] | 27 | [23 , 32] |  |
| High LDL-C | 6008 | 6.1 | [5.4 , 6.8] | 448 | 62 | [56 , 67] | 47 | [42 , 53] | 46 | [41 , 52] | 28 | [23 , 33] |  |
| Azerbaijan |  |  |  |  |  |  |  |  |  |  |  |  |  |
| High TC | 2621 | 5.4 | [4.5 , 6.6] | 193 | 28 | [20 , 37] | 18 | [12 , 26] | 16 | [10 , 24] | 0 | [0 , 0] |  |
| Bangladesh |  |  |  |  |  |  |  |  |  |  |  |  |  |
| High TC | 6929 | 4.9 | [4.2 , 5.8] | 414 | 20 | [16 , 26] | 18 | [14 , 23] | 18 | [14 , 22] | 12 | [9 , 17] |  |
| High LDL-C | 6762 | 5.0 | [4.4 , 5.8] | 405 | 19 | [15 , 24] | 16 | [12 , 21] | 15 | [12 , 20] | 12 | [9 , 16] |  |
| Belarus |  |  |  |  |  |  |  |  |  |  |  |  |  |
| High TC | 4744 | 9.9 | [8.8 , 11.1] | 583 | 88 | [83 , 91] | 47 | [42 , 52] | 45 | [40 , 50] | 0 | [0 , 0] |  |
| Benin |  |  |  |  |  |  |  |  |  |  |  |  |  |
| High TC | 4761 | 3.5 | [2.7 , 4.5] | 220 | 4 | [2 , 10] | 4 | [1 , 10] | 3 | [1 , 9] | 0 | [0 , 0] |  |
| Bhutan |  |  |  |  |  |  |  |  |  |  |  |  |  |
| High TC | 2683 | 1.5 | [1.1 , 2.2] | 46 | 14 | [5 , 35] | 9 | [4 , 23] | 9 | [4 , 23] | 0 | [0 , 0] |  |
| Botswana |  |  |  |  |  |  |  |  |  |  |  |  |  |
| High TC | 3367 | 2.7 | [1.8 , 3.8] | 104 | 36 | [19 , 59] | 28 | [12 , 54] | 28 | [12 , 54] | 0 | [0 , 0] |  |
| Burkina Faso |  |  |  |  |  |  |  |  |  |  |  |  |  |
| High TC | 4440 | 0.8 | [0.6 , 1.2] | 36 | 21 | [8 , 43] | 13 | [4 , 37] | 13 | [4 , 37] | 0 | [0 , 0] |  |
| High LDL-C | 1374 | 1.1 | [0.6 , 1.9] | 16 | 9 | [1 , 58] | 0 | [0 , 0] | 0 | [0 , 0] | 0 | [0 , 0] |  |
| Chile |  |  |  |  |  |  |  |  |  |  |  |  |  |
| High TC | 2704 | 14.8 | [12.4 , 17.6] | 465 | 68 | [58 , 77] | 51 | [41 , 61] | 30 | [22 , 39] | 16 | [11 , 23] |  |
| High LDL-C | 2628 | 13.3 | [11.2 , 15.7] | 408 | 65 | [54 , 75] | 51 | [41 , 62] | 34 | [24 , 45] | 19 | [12 , 27] |  |
| Costa Rica |  |  |  |  |  |  |  |  |  |  |  |  |  |
| High TC | 2606 | 26.2 | [21.3 , 31.8] | 774 | 82 | [73 , 89] | 74 | [66 , 80] | 71 | [64 , 77] | 20 | [16 , 25] |  |
| High LDL-C | 2395 | 26.9 | [21.1 , 33.6] | 795 | 82 | [74 , 88] | 72 | [66 , 78] | 69 | [63 , 76] | 22 | [18 , 26] |  |
| Ecuador |  |  |  |  |  |  |  |  |  |  |  |  |  |
| High TC | 3986 | 11.7 | [10.6 , 13] | 497 | 78 | [74 , 82] | 60 | [55 , 65] | 54 | [48 , 59] | 0 | [0 , 0] |  |
| Eswatini |  |  |  |  |  |  |  |  |  |  |  |  |  |
| High TC | 2889 | 2.0 | [1.5 , 2.7] | 76 | 17 | [8 , 34] | 13 | [5 , 31] | 13 | [5 , 31] | 0 | [0 , 0] |  |
| Guyana |  |  |  |  |  |  |  |  |  |  |  |  |  |
| High TC | 849 | 15.4 | [12.7 , 18.6] | 143 | 53 | [42 , 63] | 36 | [27 , 47] | 35 | [25 , 46] | 0 | [0 , 0] |  |
| Iran |  |  |  |  |  |  |  |  |  |  |  |  |  |
| High TC | 19349 | 9.7 | [9.2 , 10.3] | 1869 | 89 | [88 , 91] | 80 | [78 , 83] | 79 | [76 , 81] | 57 | [54 , 60] |  |
| High LDL-C | 19068 | 9.9 | [9.4 , 10.5] | 1894 | 89 | [87 , 90] | 78 | [76 , 81] | 76 | [74 , 79] | 57 | [54 , 60] |  |
| Iraq |  |  |  |  |  |  |  |  |  |  |  |  |  |
| High TC | 3629 | 14.1 | [12.6 , 15.8] | 623 | 55 | [49 , 60] | 47 | [41 , 52] | 45 | [40 , 51] | 22 | [18 , 28] |  |
| High LDL-C | 3538 | 14.7 | [13.2 , 16.4] | 634 | 51 | [46 , 57] | 43 | [38 , 49] | 42 | [37 , 48] | 22 | [18 , 27] |  |
| Kiribati |  |  |  |  |  |  |  |  |  |  |  |  |  |
| High TC | 1162 | 5.1 | [2.1 , 12.1] | 55 | 2 | [1 , 9] | 1 | [0 , 5] | 0 | [0 , 0] | 0 | [0 , 0] |  |
| Note:  * Number of observations, aged 15+ and nonpregnant, with a valid measurement of TC in the case of High TC and LDL-C in the case of High LDL-C ** Number of all respondents classified as having dyslipidemia based on TC / LDL-C measures or a self-reported medication status (defined by exceeding ATP III guideline   cutoffs, ie.TC≥240 mg/dL / LDL-C≥160 mg/dL or respondent taking lipid medication) † Adjusted for sampling design ‡ See note Table A | | | | | | | | | | | | |  |
|  |  |  |  |  |  |  |  |  |  |  |  |  |  |
|  |  |  |  |  |  |  |  |  |  |  |  |  |  |
|  |  |  |  |  |  |  |  |  |  |  |  |  |  |
|  |  |  |  |  |  |  |  |  |  |  |  |  |  |

Table D: Cascade of Care by Country ctd.

|  | Number of Observations With Biomarker^*^ | Prevalence of Hypercholesterolemia^†^ | | Number of Observations With Hypercholesterolemia^**^ | Lipids Measured^†‡^ | | Aware of Diagnosis^†‡^ | | Advice or Medication^†‡^ | | Controlled Disease^†‡^ | |  |
| --- | --- | --- | --- | --- | --- | --- | --- | --- | --- | --- | --- | --- | --- |
|  |  | Percent | 95% CI |  | Percent | 95% CI | Percent | 95% CI | Percent | 95% CI | Percent | 95% CI |  |
| Kyrgyzstan |  |  |  |  |  |  |  |  |  |  |  |  |  |
| High TC | 2495 | 3.8 | [3.1 , 4.6] | 116 | 37 | [26 , 48] | 24 | [15 , 35] | 23 | [14 , 34] | 0 | [0 , 0] |  |
| Lebanon |  |  |  |  |  |  |  |  |  |  |  |  |  |
| High TC | 1152 | 28.1 | [24.8 , 31.7] | 394 | 46 | [37 , 56] | 27 | [21 , 33] | 27 | [21 , 33] | 12 | [8 , 17] |  |
| High LDL-C | 1133 | 28.1 | [24.7 , 31.6] | 374 | 45 | [36 , 54] | 27 | [21 , 34] | 27 | [21 , 33] | 12 | [9 , 17] |  |
| Marshall Islands |  |  |  |  |  |  |  |  |  |  |  |  |  |
| High TC | 2716 | 5.4 | [4.6 , 6.3] | 147 | 49 | [41 , 57] | 36 | [28 , 44] | 32 | [24 , 40] | 0 | [0 , 0] |  |
| Moldova |  |  |  |  |  |  |  |  |  |  |  |  |  |
| High TC | 3695 | 6.7 | [5.7 , 7.9] | 321 | 55 | [47 , 62] | 30 | [23 , 37] | 28 | [22 , 36] | 0 | [0 , 0] |  |
| Mongolia |  |  |  |  |  |  |  |  |  |  |  |  |  |
| High TC | 1883 | 4.4 | [2.8 , 6.8] | 82 | 29 | [19 , 42] | 21 | [10 , 38] | 19 | [10 , 33] | 6 | [2 , 17] |  |
| High LDL-C | 1658 | 9.4 | [6.7 , 12.9] | 155 | 19 | [13 , 28] | 10 | [6 , 18] | 9 | [5 , 16] | 4 | [1 , 9] |  |
| Morocco |  |  |  |  |  |  |  |  |  |  |  |  |  |
| High TC | 4668 | 2.3 | [2 , 2.8] | 148 | 71 | [63 , 79] | 63 | [54 , 71] | 63 | [54 , 71] | 49 | [41 , 58] |  |
| High LDL-C | 4589 | 2.4 | [2 , 2.9] | 143 | 66 | [56 , 74] | 56 | [47 , 65] | 56 | [47 , 65] | 49 | [40 , 58] |  |
| Myanmar |  |  |  |  |  |  |  |  |  |  |  |  |  |
| High TC | 7736 | 6.3 | [5.2 , 7.6] | 678 | 8 | [5 , 13] | 6 | [4 , 9] | 6 | [4 , 9] | 3 | [2 , 4] |  |
| High LDL-C | 7162 | 6.8 | [5.6 , 8.2] | 644 | 8 | [5 , 12] | 5 | [3 , 8] | 5 | [3 , 8] | 3 | [2 , 4] |  |
| Seychelles |  |  |  |  |  |  |  |  |  |  |  |  |  |
| High TC | 1189 | 16.8 | [14.7 , 19.1] | 225 | 59 | [52 , 66] | 43 | [36 , 50] | 36 | [30 , 43] | 23 | [17 , 29] |  |
| High LDL-C | 1178 | 22.3 | [19.9 , 24.9] | 292 | 52 | [46 , 58] | 33 | [28 , 39] | 28 | [23 , 33] | 17 | [13 , 22] |  |
| Solomon Islands |  |  |  |  |  |  |  |  |  |  |  |  |  |
| High TC | 1666 | 6.5 | [5.3 , 7.9] | 115 | 1 | [0 , 6] | 0 | [0 , 2] | 0 | [0 , 2] | 0 | [0 , 0] |  |
| Sri Lanka |  |  |  |  |  |  |  |  |  |  |  |  |  |
| High TC | 4460 | 9.8 | [8.8 , 10.9] | 558 | 73 | [67 , 77] | 60 | [55 , 65] | 59 | [54 , 64] | 0 | [0 , 0] |  |
| St. Vincent & The Grenadines |  |  |  |  |  |  |  |  |  |  |  |  |  |
| High TC | 1009 | 7.6 | [5.6 , 10.2] | 104 | 51 | [31 , 70] | 30 | [20 , 42] | 29 | [20 , 41] | 9 | [3 , 19] |  |
| High LDL-C | 839 | 10.3 | [7.6 , 13.9] | 107 | 43 | [30 , 57] | 23 | [15 , 33] | 22 | [15 , 32] | 8 | [3 , 16] |  |
| Sudan |  |  |  |  |  |  |  |  |  |  |  |  |  |
| High TC | 6760 | 3.1 | [2.6 , 3.7] | 273 | 27 | [20 , 34] | 20 | [15 , 27] | 20 | [15 , 27] | 0 | [0 , 0] |  |
| Tajikistan |  |  |  |  |  |  |  |  |  |  |  |  |  |
| High TC | 2595 | 1.5 | [1 , 2.2] | 70 | 22 | [12 , 37] | 14 | [7 , 26] | 14 | [7 , 26] | 0 | [0 , 0] |  |
| Timor-Leste |  |  |  |  |  |  |  |  |  |  |  |  |  |
| High TC | 2431 | 0.8 | [0.5 , 1.3] | 19 | 42 | [21 , 66] | 42 | [21 , 66] | 42 | [21 , 66] | 0 | [0 , 0] |  |
| Tokelau |  |  |  |  |  |  |  |  |  |  |  |  |  |
| High TC | 511 | 14.2 | [6.6 , 27.7] | 87 | 57 | [29 , 82] | 31 | [14 , 55] | 29 | [16 , 46] | 0 | [0 , 0] |  |
| Tonga |  |  |  |  |  |  |  |  |  |  |  |  |  |
| High TC | 3595 | 11.2 | [9 , 13.9] | 436 | 60 | [52 , 67] | 40 | [34 , 45] | 38 | [32 , 44] | 0 | [0 , 0] |  |
| Tuvalu |  |  |  |  |  |  |  |  |  |  |  |  |  |
| High TC | 1012 | 3.4 | [2.2 , 5.1] | 35 | 36 | [24 , 49] | 29 | [16 , 48] | 29 | [16 , 48] | 0 | [0 , 0] |  |
| Vietnam |  |  |  |  |  |  |  |  |  |  |  |  |  |
| High TC | 2981 | 8.4 | [7.2 , 9.7] | 294 | 46 | [39 , 54] | 28 | [23 , 35] | 24 | [19 , 30] | 0 | [0 , 0] |  |
| Zambia |  |  |  |  |  |  |  |  |  |  |  |  |  |
| High TC | 3635 | 1.5 | [1.1 , 1.9] | 78 | 5 | [2 , 11] | 3 | [1 , 7] | 1 | [0 , 5] | 0 | [0 , 0] |  |
| Note:  * Number of observations, aged 15+ and nonpregnant, with a valid measurement of TC in the case of High TC and LDL-C in the case of High LDL-C ** Number of all respondents classified as having dyslipidemia based on TC / LDL-C measures or a self-reported medication status (defined by exceeding ATP III guideline   cutoffs, ie.TC≥240 mg/dL / LDL-C≥160 mg/dL or respondent taking lipid medication) † Adjusted for sampling design ‡ See note Table A | | | | | | | | | | | | |  |
|  |  |  |  |  |  |  |  |  |  |  |  |  |  |
|  |  |  |  |  |  |  |  |  |  |  |  |  |  |
|  |  |  |  |  |  |  |  |  |  |  |  |  |  |
|  |  |  |  |  |  |  |  |  |  |  |  |  |  |

Table E: Predictors of Cascade Progression – Univariable, Poisson

|  | Measured | | | Diagnoses | | | Treated | | | Controlled | | |
| --- | --- | --- | --- | --- | --- | --- | --- | --- | --- | --- | --- | --- |
|  | RR | | P | RR | | P | RR | | P | RR | | P |
| Age | N = 11767 | | | N = 6823 | | | N = 5176 | | | N = 4842 | | |
| 15-24 years | REF | | | REF | | | REF | | | REF | | |
| 25-34 years | 1.20 | [0.96,1.49] | 0.12 | 1.07 | [0.82,1.38] | 0.62 | 0.94 | [0.84,1.05] | 0.27 | 1.13 | [0.60,2.12] | 0.71 |
| 35-44 years | 1.77 | [1.43,2.18] | <0.001 | 1.30 | [1.02,1.64] | 0.03 | 0.99 | [0.91,1.09] | 0.91 | 1.30 | [0.72,2.36] | 0.38 |
| 45-54 years | 2.08 | [1.69,2.56] | <0.001 | 1.44 | [1.14,1.82] | 0.002 | 1.01 | [0.92,1.11] | 0.83 | 1.38 | [0.76,2.49] | 0.29 |
| 55-64 years | 2.33 | [1.90,2.87] | <0.001 | 1.52 | [1.21,1.92] | <0.001 | 1.03 | [0.94,1.13] | 0.55 | 1.45 | [0.80,2.61] | 0.22 |
| 65 or older | 2.33 | [1.90,2.87] | <0.001 | 1.49 | [1.18,1.88] | <0.001 | 1.05 | [0.96,1.15] | 0.32 | 1.64 | [0.91,2.96] | 0.10 |
| Sex | N = 11766 | | | N = 6822 | | | N = 5175 | | | N = 4841 | | |
| Male | REF | | | REF | | | REF | | | REF | | |
| Female | 1.06 | [1.03,1.10] | <0.001 | 1.01 | [0.99,1.04] | 0.31 | 0.99 | [0.98,1.01] | 0.30 | 0.92 | [0.87,0.97] | 0.002 |
| Education | N = 11479 | | | N = 6631 | | | N = 5026 | | | N = 4696 | | |
| Less than primary school | REF | | | REF | | | REF | | | REF | | |
| Less than secondary school | 0.96 | [0.93,1.00] | 0.04 | 0.99 | [0.96,1.02] | 0.58 | 1.00 | [0.98,1.01] | 0.60 | 0.98 | [0.91,1.05] | 0.53 |
| Secondary school completed or higher | 1.05 | [1.01,1.09] | 0.02 | 0.95 | [0.92,0.99] | 0.006 | 0.98 | [0.96,1.00] | 0.01 | 0.94 | [0.88,1.02] | 0.13 |
| Smoking Status | N = 11762 | | | N = 6819 | | | N = 5173 | | | N = 4839 | | |
| Past or Never | REF | | | REF | | | REF | | | REF | | |
| Current | 0.89 | [0.85,0.93] | <0.001 | 0.93 | [0.89,0.98] | 0.003 | 0.97 | [0.95,0.99] | 0.01 | 1.01 | [0.92,1.11] | 0.82 |
| BMI | N = 11520 | | | N = 6660 | | | N = 5043 | | | N = 4714 | | |
| Normal Weight | REF | | | REF | | | REF | | | REF | | |
| Underweight | 0.68 | [0.57,0.80] | <0.001 | 0.98 | [0.85,1.14] | 0.80 | 0.98 | [0.91,1.06] | 0.68 | 1.16 | [0.91,1.47] | 0.23 |
| Overweight | 1.12 | [1.08,1.16] | <0.001 | 1.09 | [1.05,1.13] | <0.001 | 0.99 | [0.97,1.01] | 0.38 | 1.02 | [0.95,1.10] | 0.59 |
| Obese | 1.24 | [1.20,1.29] | <0.001 | 1.11 | [1.07,1.15] | <0.001 | 1.00 | [0.98,1.02] | 0.90 | 0.99 | [0.92,1.07] | 0.82 |
| Diabetic | N = 11080 | | | N = 6401 | | | N = 4863 | | | N = 4537 | | |
|  | 1.30 | [1.27,1.34] | <0.001 | 1.14 | [1.11,1.16] | <0.001 | 1.04 | [1.02,1.05] | <0.001 | 1.24 | [1.17,1.31] | <0.001 |
| Hypertensive | N = 11700 | | | N = 6790 | | | N = 5153 | | | N = 4821 | | |
|  | 1.31 | [1.27,1.35] | <0.001 | 1.14 | [1.11,1.18] | <0.001 | 1.05 | [1.03,1.07] | <0.001 | 1.11 | [1.05,1.18] | <0.001 |
| Exponentiated coefficients; 95% confidence intervals in brackets | | | |  |  |  |  |  |  |  |  |  |

Table F: Predictors of Cascade Progression – Multivariable Linear Probability Specification

|  | Measured | | | Diagnosed | | | Treated | | | Controlled | | |  |
| --- | --- | --- | --- | --- | --- | --- | --- | --- | --- | --- | --- | --- | --- |
|  | LP | | P | LP | | P | LP | | P | LP | | P |  |
| Age |  | |  |  | |  |  | |  |  | |  |  |
| 15-24 years | REF | | | REF | | | REF | | | REF | | |  |
| 25-34 years | 0.05 | [-0.02,0.11] | 0.14 | 0.06 | [-0.09,0.20] | 0.44 | -0.08 | [-0.18,0.01] | 0.08 | 0.04 | [-0.13,0.21] | 0.66 |  |
| 35-44 years | 0.15 | [0.09,0.21] | <0.001 | 0.15 | [0.02,0.29] | 0.03 | -0.05 | [-0.13,0.03] | 0.24 | 0.07 | [-0.09,0.23] | 0.37 |  |
| 45-54 years | 0.21 | [0.15,0.27] | <0.001 | 0.2 | [0.07,0.34] | 0.003 | -0.04 | [-0.11,0.04] | 0.35 | 0.09 | [-0.07,0.24] | 0.28 |  |
| 55-64 years | 0.27 | [0.21,0.33] | <0.001 | 0.23 | [0.10,0.36] | 0.001 | -0.02 | [-0.10,0.05] | 0.55 | 0.1 | [-0.06,0.25] | 0.21 |  |
| 65 or older | 0.30 | [0.24,0.36] | <0.001 | 0.21 | [0.08,0.35] | 0.002 | -0.01 | [-0.09,0.06] | 0.73 | 0.16 | [-0.00,0.31] | 0.05 |  |
| Sex |  | |  |  | |  |  | |  |  | |  |  |
| Male | REF | | | REF | | | REF | | | REF | | |  |
| Female | 0.03 | [0.01,0.05] | 0.002 | 0.01 | [-0.02,0.03] | 0.52 | -0.01 | [-0.03,0.01] | 0.21 | -0.04 | [-0.07,-0.01] | 0.003 |  |
| Education |  | |  |  | |  |  | |  |  | |  |  |
| Less than primary school | REF | | | REF | | | REF | | | REF | | |  |
| Less than secondary school | 0.03 | [0.01,0.05] | 0.01 | 0.01 | [-0.02,0.04] | 0.51 | 0.01 | [-0.01,0.02] | 0.45 | 0.01 | [-0.03,0.05] | 0.61 |  |
| Secondary school completed or higher | 0.11 | [0.09,0.14] | <0.001 | 0.01 | [-0.03,0.04] | 0.67 | 0.00 | [-0.02,0.02] | 0.96 | 0.00 | [-0.04,0.04] | 0.90 |  |
| Smoking |  | |  |  | |  |  | |  |  | |  |  |
| Past or Never | REF | | | REF | | | REF | | | REF | | |  |
| Current | -0.02 | [-0.05,0.00] | 0.08 | -0.02 | [-0.06,0.01] | 0.16 | -0.03 | [-0.05,-0.00] | 0.03 | 0.01 | [-0.04,0.05] | 0.76 |  |
| BMI |  |  |  |  |  |  |  |  |  |  |  |  |  |
| Normal | REF | | | REF | | | REF | | | REF | | |  |
| Underweight | -0.08 | [-0.13,-0.03] | 0.001 | 0.01 | [-0.10,0.11] | 0.89 | -0.02 | [-0.09,0.06] | 0.64 | 0.04 | [-0.05,0.13] | 0.41 |  |
| Overweight | 0.03 | [0.01,0.05] | 0.001 | 0.06 | [0.03,0.08] | <0.001 | -0.01 | [-0.03,0.01] | 0.22 | 0.01 | [-0.02,0.05] | 0.43 |  |
| Obese | 0.08 | [0.06,0.10] | <0.001 | 0.05 | [0.03,0.08] | <0.001 | -0.01 | [-0.03,0.01] | 0.48 | 0.00 | [-0.04,0.04] | 0.98 |  |
| Diabetes | 0.11 | [0.10,0.13] | <0.001 | 0.07 | [0.05,0.10] | <0.001 | 0.02 | [0.01,0.04] | 0.001 | 0.09 | [0.06,0.11] | <0.001 |  |
| Hypertension | 0.08 | [0.06,0.09] | <0.001 | 0.06 | [0.04,0.09] | <0.001 | 0.03 | [0.02,0.05] | <0.001 | 0.02 | [-0.01,0.05] | 0.16 |  |
| *N* | 10575 | | | 6073 | | | 4601 | | | 4283 | | |  |
| 95% confidence intervals in brackets |  |  |  |  |  |  |  |  |  |  |  |  |  |

Table G: Predictors of Cascade Progression - Multivariable Basic Poisson Specification

|  | Measured | | | Diagnosed | | | Treated | | | Controlled | | |
| --- | --- | --- | --- | --- | --- | --- | --- | --- | --- | --- | --- | --- |
|  | RR | | P | RR | | P | RR | | P | RR | | P |
| Age |  | |  |  | |  |  | |  |  | |  |
| 15-24 years | REF | | | REF | | | REF | | | REF | | |
| 25-34 years | 1.16 | [0.91,1.48] | 0.24 | 1.11 | [0.83,1.47] | 0.49 | 0.90 | [0.82,0.99] | 0.02 | 1.24 | [0.64,2.41] | 0.53 |
| 35-44 years | 1.87 | [1.47,2.39] | <0.001 | 1.36 | [1.04,1.76] | 0.02 | 0.95 | [0.88,1.02] | 0.15 | 1.45 | [0.78,2.70] | 0.24 |
| 45-54 years | 2.36 | [1.85,3.00] | <0.001 | 1.46 | [1.12,1.90] | 0.005 | 0.96 | [0.90,1.04] | 0.33 | 1.53 | [0.83,2.82] | 0.17 |
| 55-64 years | 2.8 | [2.20,3.55] | <0.001 | 1.50 | [1.15,1.95] | 0.002 | 0.97 | [0.90,1.04] | 0.41 | 1.36 | [0.74,2.51] | 0.32 |
| 65 or older | 3.47 | [2.73,4.41] | <0.001 | 1.50 | [1.15,1.95] | 0.003 | 0.97 | [0.91,1.04] | 0.44 | 1.66 | [0.90,3.06] | 0.10 |
| Sex |  | |  |  | |  |  | |  |  | |  |
| Male | REF | | | REF | | | REF | | | REF | | |
| Female | 1.02 | [0.99,1.06] | 0.20 | 0.99 | [0.96,1.01] | 0.34 | 0.98 | [0.96,1.00] | 0.03 | 0.76 | [0.71,0.82] | <0.001 |
| Education |  | |  |  | |  |  | |  |  | |  |
| Less than primary school | REF | | | REF | | | REF | | | REF | | |
| Less than secondary school | 1.04 | [0.99,1.09] | 0.15 | 0.94 | [0.91,0.98] | 0.003 | 0.97 | [0.95,0.99] | 0.001 | 0.74 | [0.68,0.80] | <0.001 |
| Secondary school completed or higher | 1.23 | [1.16,1.30] | <0.001 | 0.86 | [0.82,0.89] | <0.001 | 0.94 | [0.92,0.96] | <0.001 | 0.46 | [0.40,0.52] | <0.001 |
| *N* | 11478 | | | 6630 | | | 5025 | | | 4695 | | |
| Exponentiated coefficients;  95% confidence intervals in brackets | | |  |  |  |  |  |  |  |  |  |  |

Table H: Analysis of Deviance for Main Modified Poisson Regression Specifications (Table 2 Main Manuscript)

|  | Measured | | | Diagnosed | | | Treated | | | Controlled | | |
| --- | --- | --- | --- | --- | --- | --- | --- | --- | --- | --- | --- | --- |
|  | Degrees of Freedom | X2 | p-value | Degrees of Freedom | X2 | p-value | Degrees of Freedom | X2 | p-value | Degrees of Freedom | X2 | p-value |
| Age | 5 | 837 | <0.001 | 5 | 64 | <0.001 | 5 | 18 | 0.002 | 5 | 78 | <0.001 |
| Sex | 1 | 0 | 0.88 | 1 | 0 | 0.52 | 1 | 2 | 0.16 | 1 | 22 | <0.001 |
| Education | 2 | 140 | <0.001 | 2 | 69 | <0.001 | 2 | 31 | <0.001 | 2 | 203 | <0.001 |
| Smoking | 1 | 17 | <0.001 | 1 | 16 | <0.001 | 1 | 20 | <0.001 | 1 | 1 | 0.29 |
| BMI | 3 | 292 | <0.001 | 3 | 15 | 0.002 | 3 | 3 | 0.37 | 3 | 11 | 0.008 |
| Diabetes | 1 | 135 | <0.001 | 1 | 93 | <0.001 | 1 | 37 | <0.001 | 1 | 50 | <0.001 |
| Hypertension | 1 | 8 | 0.003 | 1 | 9 | 0.003 | 1 | 23 | <0.001 | 1 | 10 | 0.001 |
| Note: Analysis of Deviance for the main modified Poisson regression specifications with outcomes and “Lipids Measured”, “Aware of Diagnosis”, “Advice or Medication”, and “Controlled Disease” as dependent variables; Wald statistic; terms added sequentially (first to last) | | | | | | | | | | | | |
