# Supplementary material for: Unmet need for hypercholesterolemia care in 35 low- and middle-income countries: A cross-sectional study of nationally representative surveys
Source: PLoS Med. 2021 Oct 25;18(10):e1003841. doi: 10.1371/journal.pmed.1003841 (PMC8575312; doi:10.1371/journal.pmed.1003841)
Supplement: S3 Table — (DOCX) [file pmed.1003841.s009.docx]

# S3 Table: Missing Predictor Variables by Country Amongst Participants with Hypercholesterolemia, by Country

|  | Sex | | Age | | Education | | BMI | | Smoking | | Diabetes | | Hypertension | |
| --- | --- | --- | --- | --- | --- | --- | --- | --- | --- | --- | --- | --- | --- | --- |
|  | N | % | N | % | N | % | N | % | N | % | N | % | N | % |
| Algeria |  | 0 |  | 0 | 3 | 0.6 | 22 | 4.1 | 2 | 0 | 39 | 7.2 |  | 0 |
| Azerbaijan |  | 0 |  | 0 | 1 | 0.5 | 7 | 3.6 |  | 0 | 2 | 1.0 | 1 | 0.5 |
| Bangladesh |  | 0 |  | 0 | 2 | 0.4 | 17 | 3.3 |  | 0 |  | 0 | 2 | 0.4 |
| Belarus |  | 0 |  | 0 |  | 0 | 3 | 0.5 |  | 0 | 25 | 4.3 |  | 0 |
| Benin |  | 0 |  | 0 |  | 0 | 17 | 7.2 |  | 0 |  | 0 |  | 0 |
| Bhutan |  | 0 |  | 0 |  | 0 | 5 | 9.8 |  | 0 |  | 0 |  | 0 |
| Botswana |  | 0 |  | 0 |  | 0 | 2 | 1.9 |  | 0 | 4 | 3.8 |  | 0 |
| Burkina Faso |  | 0 |  | 0 |  | 0 | 1 | 2.1 |  | 0 | 6 | 12.8 |  | 0 |
| Chile |  | 0 |  | 0 | 6 | 1.2 | 13 | 2.5 | 5 | 1.0 |  | 0 | 6 | 1.2 |
| Costa Rica | 1 | 0.1 | 15 | 1.6 | 57 | 6.3 | 75 | 8.2 |  | 0 | 36 | 4.0 |  | 0 |
| Ecuador |  | 0 |  | 0 | 2 | 0.4 | 17 | 3.3 |  | 0 | 7 | 1.4 | 3 | 0.6 |
| Eswatini |  | 0 |  | 0 | 5 | 6.3 | 10 | 12.5 | 5 | 6 | 2 | 2.5 | 6 | 7.5 |
| Guyana |  | 0 |  | 0 | 1 | 0.7 | 1 | 0.7 |  | 0 | 4 | 2.8 | 1 | 0.7 |
| Iran |  | 0 |  | 0 | 91 | 4 | 88 | 4.2 | 21 | 1 | 35 | 1.7 | 22 | 1.0 |
| Iraq |  | 0 | 3 | 0.4 |  | 0 | 25 | 3.3 |  | 0 | 3 | 0.4 | 4 | 0.5 |
| Kiribati |  | 0 |  | 0 | 2 | 3 | 5 | 8.6 | 2 | 3 | 3 | 5.2 | 1 | 1.7 |
| Kyrgyzstan |  | 0 |  | 0 |  | 0 | 15 | 11.6 |  | 0 | 1 | 0.8 | 1 | 0.8 |
| Lebanon |  | 0 |  | 0 | 58 | 14 | 30 | 7.0 |  | 0 | 11 | 2.6 | 28 | 6.5 |
| Marshall Islands |  | 0 |  | 0 | 1 | 1 | 14 | 8.5 | 2 | 1 | 1 | 0.6 | 1 | 0.6 |
| Moldova |  | 0 |  | 0 |  | 0 | 17 | 5.2 |  | 0 |  | 0 | 6 | 1.8 |
| Mongolia |  | 0 |  | 0 |  | 0 | 6 | 3.3 |  | 0 | 4 | 2.2 | 1 | 0.6 |
| Morocco |  | 0 |  | 0 |  | 0 | 11 | 6.3 |  | 0 | 7 | 4.0 |  | 0 |
| Myanmar |  | 0 |  | 0 | 5 | 0.6 | 25 | 2.9 |  | 0 | 11 | 1.3 | 2 | 0.2 |
| Seychelles |  | 0 |  | 0 |  | 0 | 1 | 0.3 |  | 0 |  | 0 |  | 0 |
| Solomon Islands |  | 0 |  | 0 | 1 | 0.8 | 5 | 4.2 | 1 | 1 |  | 0 |  | 0 |
| Sri Lanka |  | 0 |  | 0 | 1 | 0.2 | 13 | 2.3 |  | 0 | 18 | 3.2 | 2 | 0 |
| St. Vincent & the Grenadines |  | 0 |  | 0 |  | 0 | 2 | 1.5 |  | 0 | 7 | 5.3 |  | 0 |
| Sudan |  | 0 |  | 0 |  | 0 | 28 | 9.3 |  | 0 | 15 | 5.0 |  | 0 |
| Tajikistan |  | 0 |  | 0 |  | 0 | 2 | 2.8 |  | 0 | 4 | 5.6 | 1 | 1.4 |
| Timor-Leste |  | 0 |  | 0 |  | 0 | 4 | 17.4 |  | 0 | 3 | 13.0 |  | 0 |
| Tokelau |  | 0 |  | 0 | 88 | 100 | 3 | 3.4 |  | 0 | 1 | 1.1 |  | 0 |
| Tonga |  | 0 |  | 0 |  | 0 | 31 | 6.9 |  | 0 | 450 | 100 | 4 | 1 |
| Tuvalu |  | 0 |  | 0 |  | 0 | 2 | 5.7 |  | 0 | 1 | 2.9 |  | 0 |
| Vietnam |  | 0 |  | 0 |  | 0 | 14 | 4.6 |  | 0 |  | 0 |  | 0 |
| Zambia |  | 0 |  | 0 |  | 0 | 4 | 4.9 |  | 0 | 5 | 6.1 | 1 | 1.2 |
