# Supplementary material for: Unmet need for hypercholesterolemia care in 35 low- and middle-income countries: A cross-sectional study of nationally representative surveys
Source: PLoS Med. 2021 Oct 25;18(10):e1003841. doi: 10.1371/journal.pmed.1003841 (PMC8575312; doi:10.1371/journal.pmed.1003841)
Supplement: S1 Fig — (DOCX) [file pmed.1003841.s010.docx]

# S1 Fig: Supplementary Analysis

Figure A: Cascade of Care for High TC with Borderline High TC Values Classified as Hypercholesterolemia


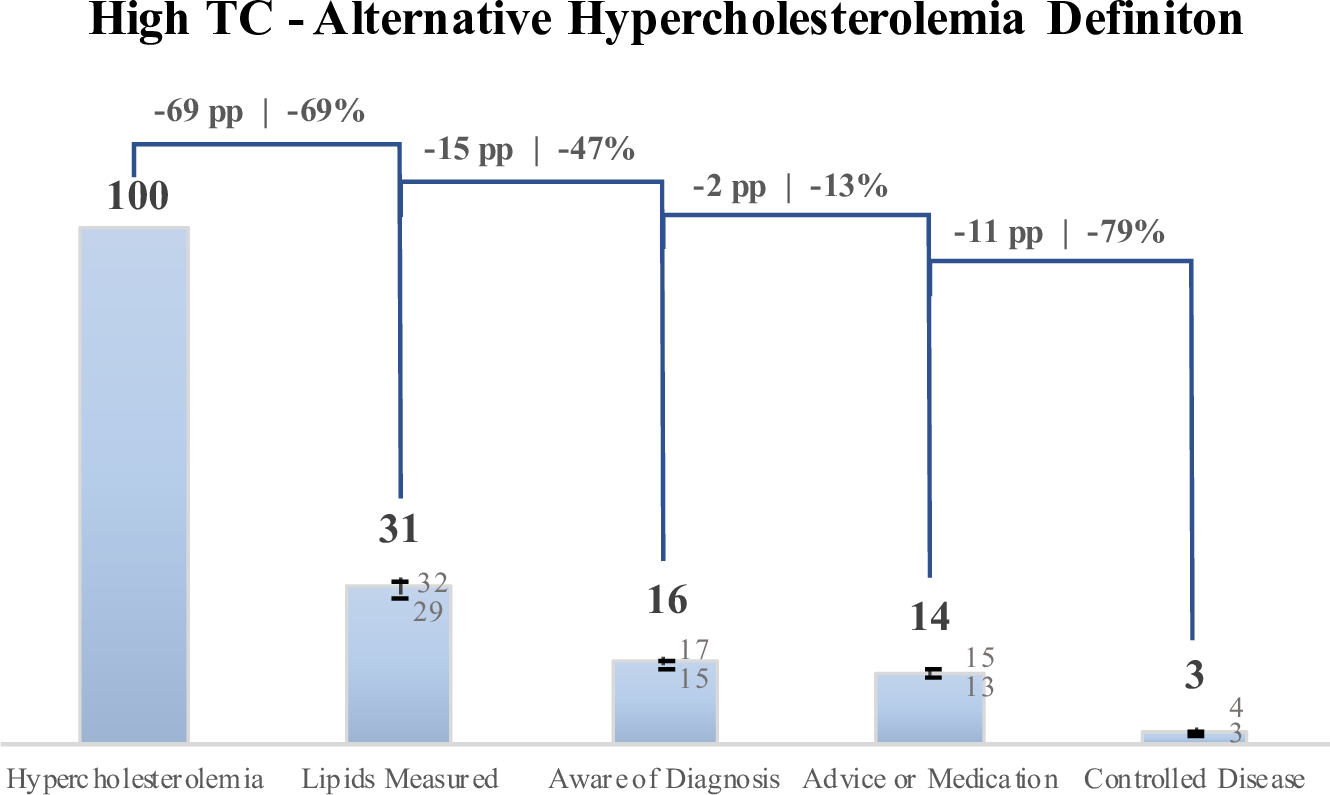


Note: Point estimates are represented by bars and shown in numeric form above bars, 95% confidence intervals are indicated by upper and lower bounds in numeric form and by whiskers. On top, the absolute percentage point drops of each cascade step are shown on the left-hand side and the relative percent drop on the right-hand side. All calculations incorporate Primary Sampling Units and strata to account for the different survey designs of included countries, as well as use sampling weights rescaled such that all countries contribute equally; Hypercholesterolemia refers to all respondents that are classified as having high TC (≥ 200 mg/dL) or a self-reported medication status. Lipids Measured refers to the percentage share of all respondents with high TC that have ever had their lipid status measured as per self-reported information. Accordingly, Aware of Diagnosis refers to the percentage share of all that have (self-reportedly) ever been diagnosed by a medical professional with hypercholesterolemia whereas Advice or Medication refers to those that have received medication or lifestyle advice for their disease. Controlled Disease considers those respondents that have TC and LDL-C values within the range considered normal by ATP III guidelines.

Figure B: Cascade of Care for High LDL-C with Borderline High LDL-C Values Classified as Hypercholesterolemia


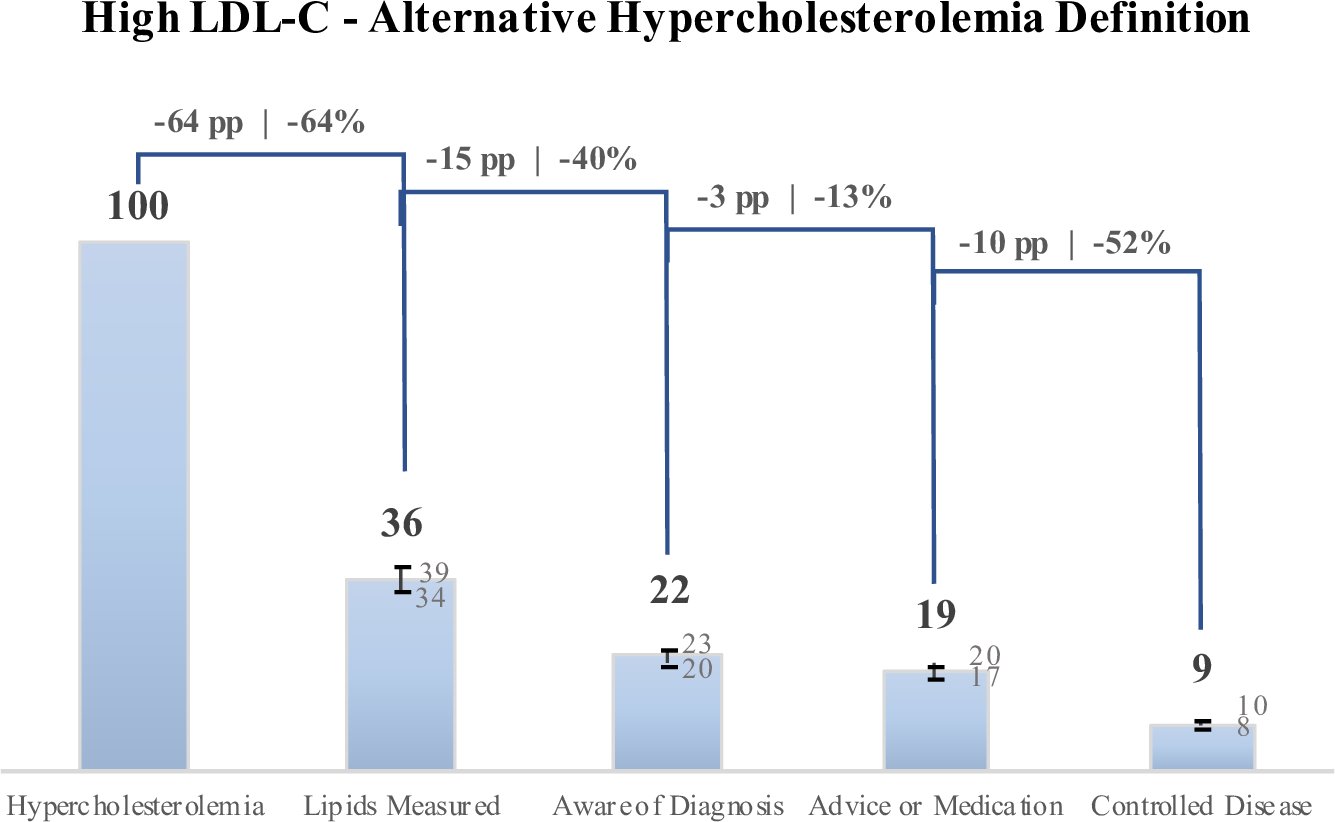


Note: See Note Figure A; Hypercholesterolemia refers to all respondents that are classified as having high LDL-C (≥ 130 mg/dL) or a self-reported medication status.

Figure C: Cascade of Care for High LDL-C based on AHA/ACC Guidelines


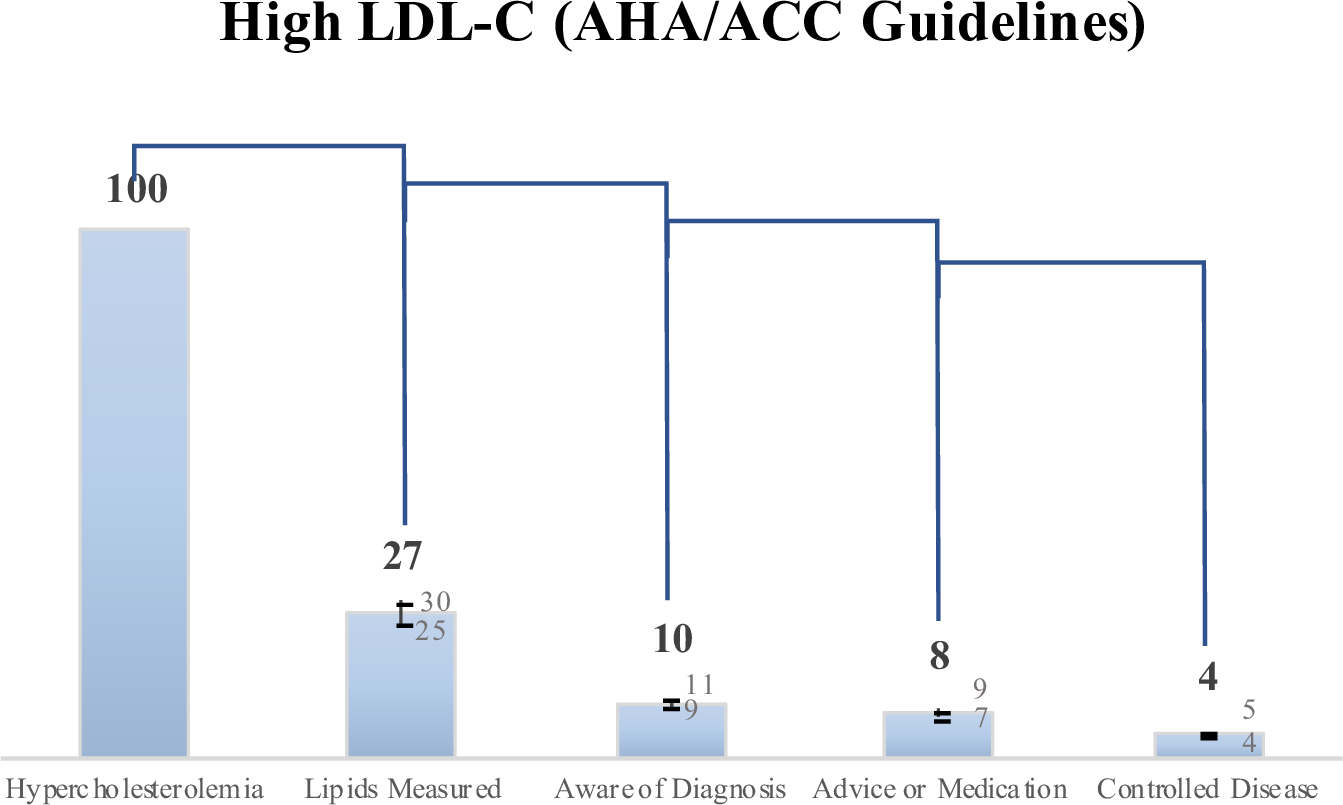


Note: See Note Figure A; Hypercholesterolemia refers to all respondents that are classified as having high LDL-C (≥ 70 mg/dL) or a self-reported medication status.

Figure D: Cascade of Care of high TC Including Respondents with Missing Biomarker


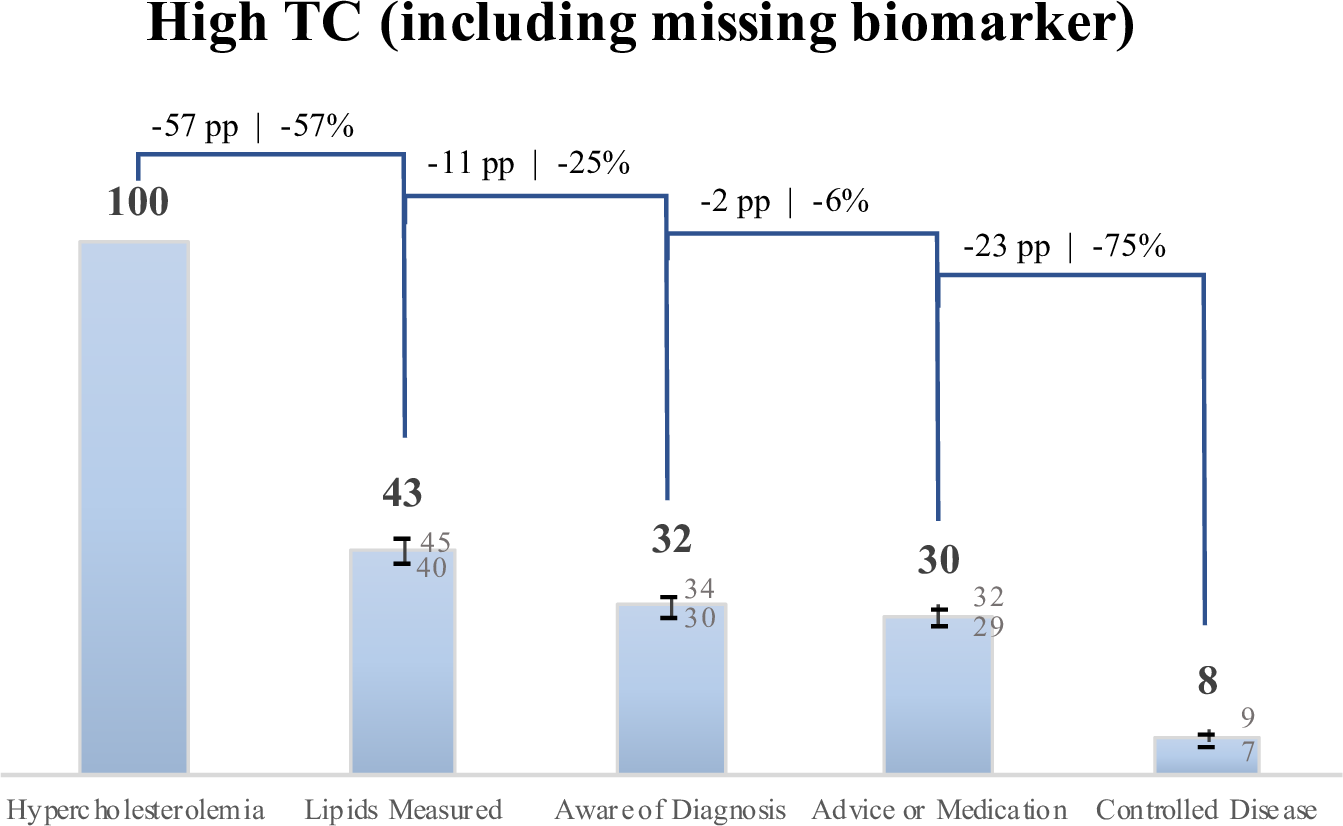


Note: See Note Figure A; Hypercholesterolemia refers to all respondents that are classified as having high TC (≥240 mg/dL) or a self-reported medication status. Also includes respondents for whom no TC measure was available. In those cases hypercholesterolemia is based on the self-reported medication status only.

Figure E: Cascade of Care for High TC, No Upper Bound for Plausible TC Values Imposed


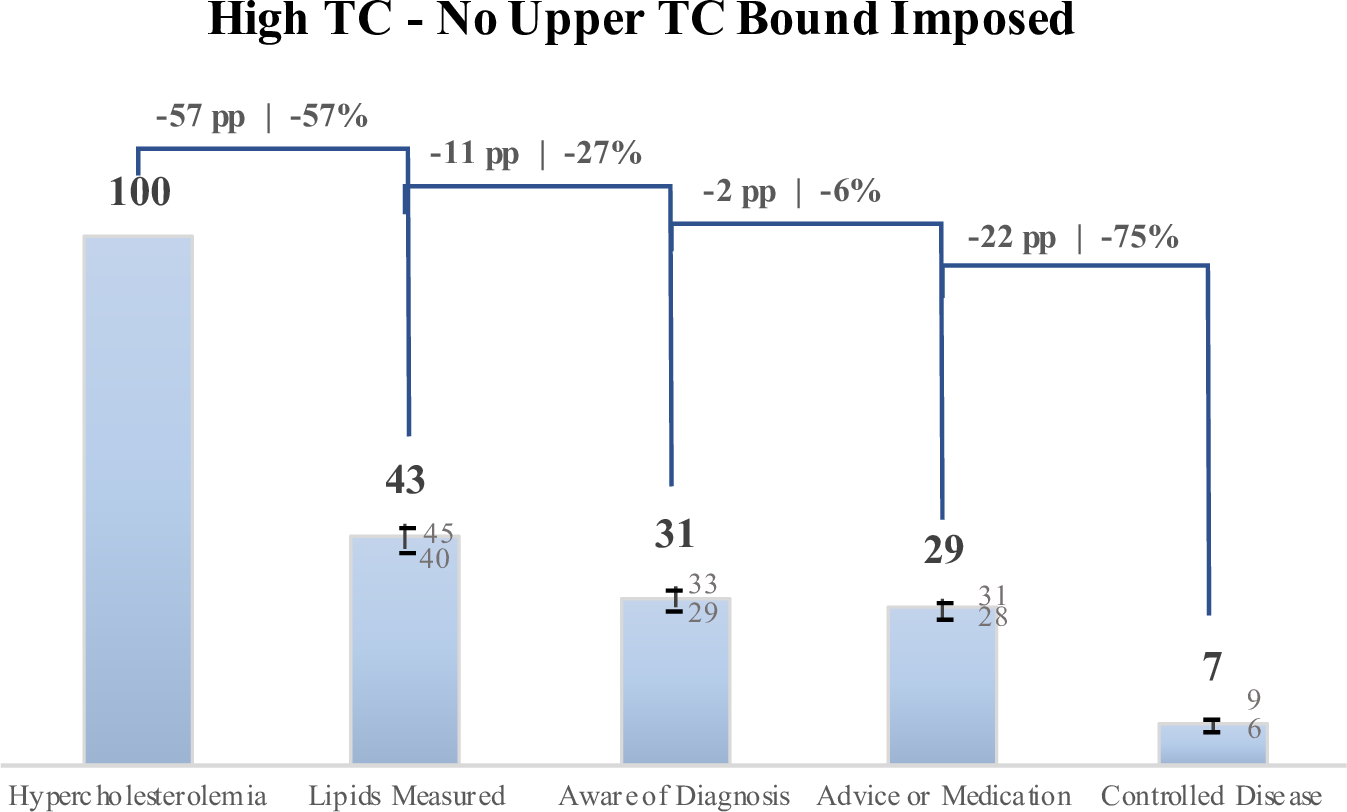


See note Figure A; Hypercholesterolemia refers to all respondents that are classified as having high TC (≥240 mg/dL – including observations with TC values above 300 mg/dl) or a self-reported medication status. Consecutive cascade stages are all based on the denominator of all respondents classified as having hypercholesterolemia.

Figure F: Cascade of Care for High TC, Alternative Controlled Disease Definition Applied


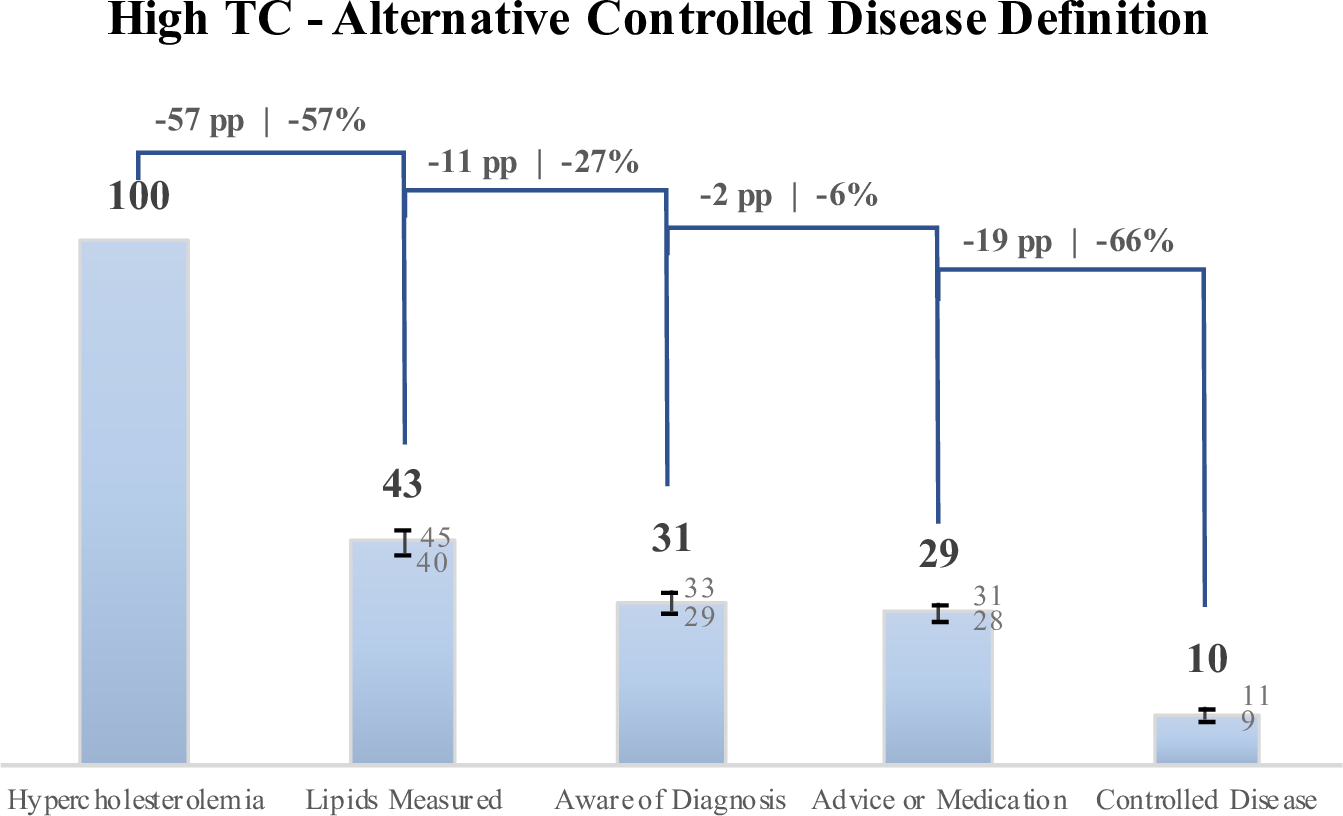


See note Figure A; Hypercholesterolemia refers to all respondents that are classified as having high TC (≥240 mg/dL) or a self-reported medication status. Controlled Disease considers those respondents that have TC and LDL-C values within the range considered normal or borderline high by ATP III guidelines.

Figure G: Cascade of Care for High TC in Countries with Non-missing LDL-C Records


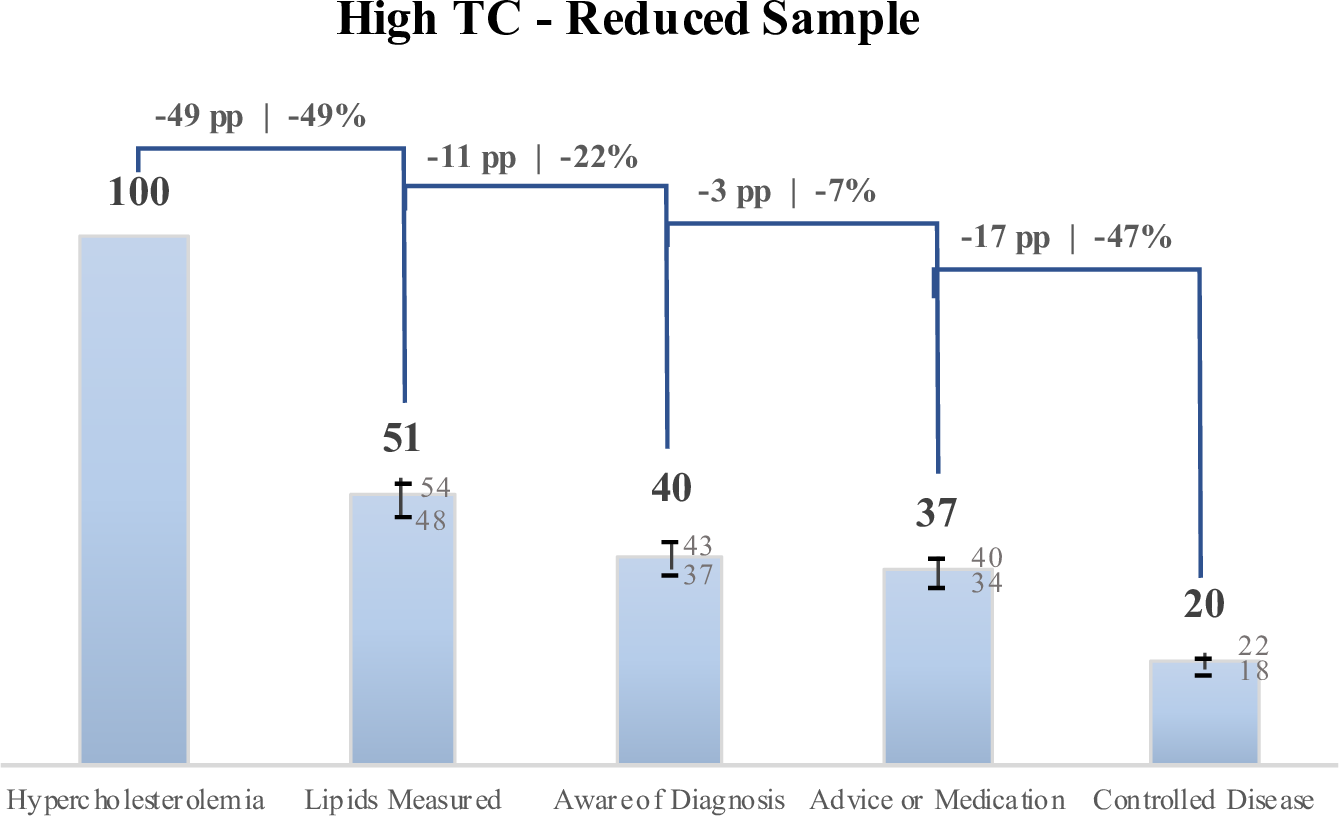


Note: See Note Figure A; Hypercholesterolemia refers to all respondents that are classified as having high TC (≥240 mg/dL) or a self-reported medication status. Included countries are Algeria, Bangladesh, Burkina Faso, Chile, Costa Rica, Iran, Iraq, Lebanon, Mongolia, Morocco, Myanmar, Seychelles, and St. Vincent & the Grenadines.

Figure H: Cascade of Care for High TC in Countries with Non-missing LDL-C Records, Restricted to Screening Recommended Sample


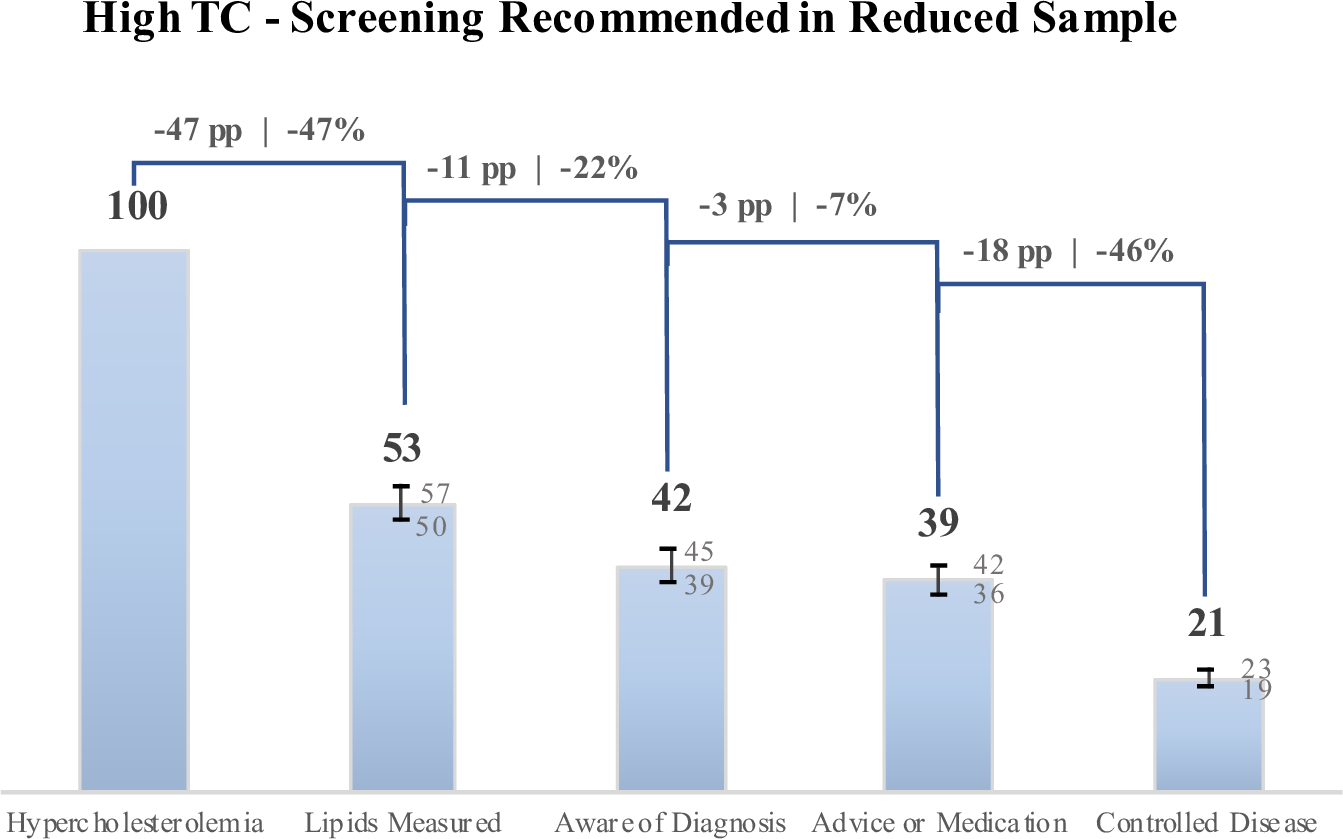


See note Figure A; Hypercholesterolemia refers to all respondents that are classified as having high TC (≥240 mg/dL) or a self-reported medication status and for whom screening is recommended based on the exhibition of at least one of the following risk factors: age>40; smoking; diabetic; hypertensive; waist circumference≥90 in males; waist circumference≥100 in females.

Consecutive cascade stages are all based on the denominator of all respondents classified as having hypercholesterolemia.

Figure I: Cascade of Care for High TC amongst Respondents Aged $\boldsymbol{\geq}$40


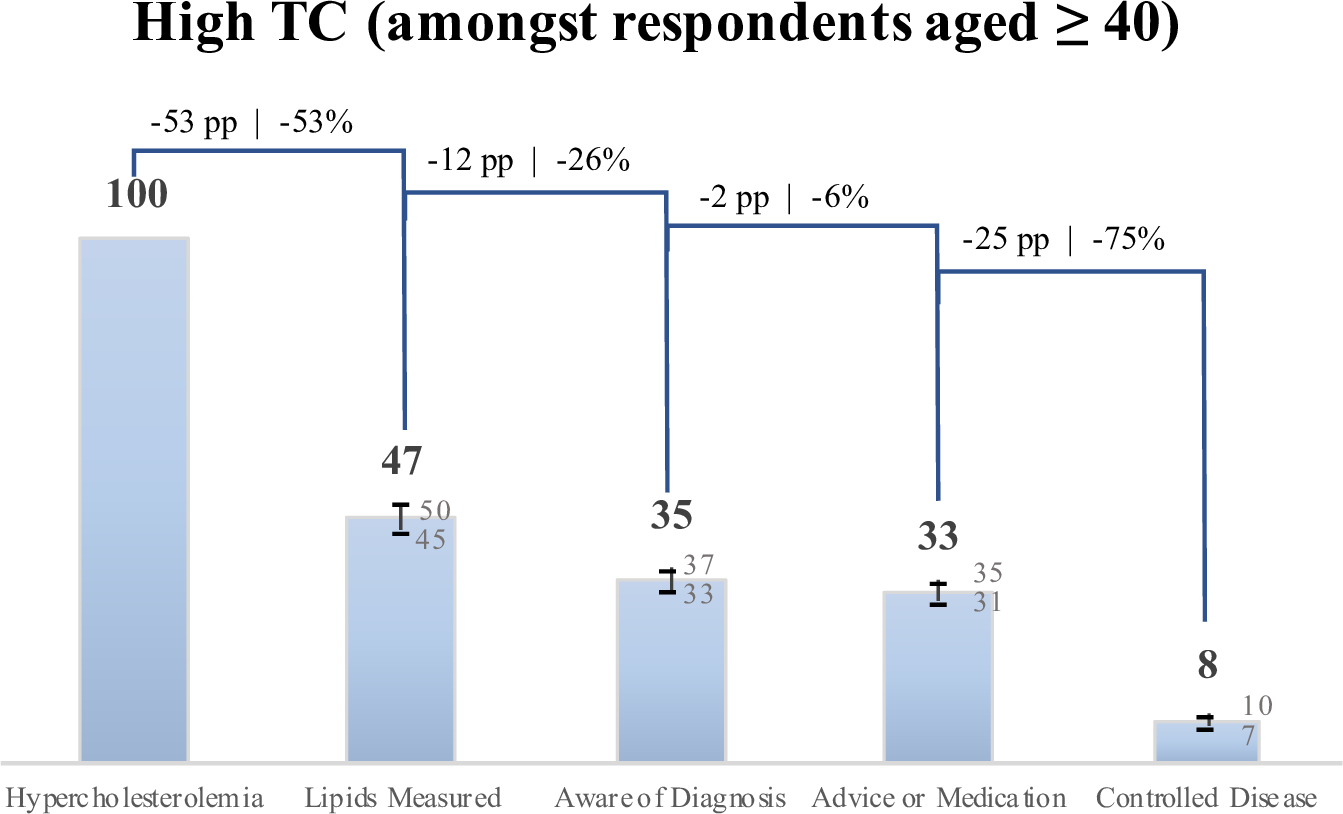


Note: See Note Figure A; Hypercholesterolemia refers to all respondents that are classified as having high TC (≥240 mg/dL) or a self-reported medication status. Cascade restricted to respondents aged 40 or older.

Figure J: Cascade of Care for High LDL-C amongst Respondents Aged ≥40


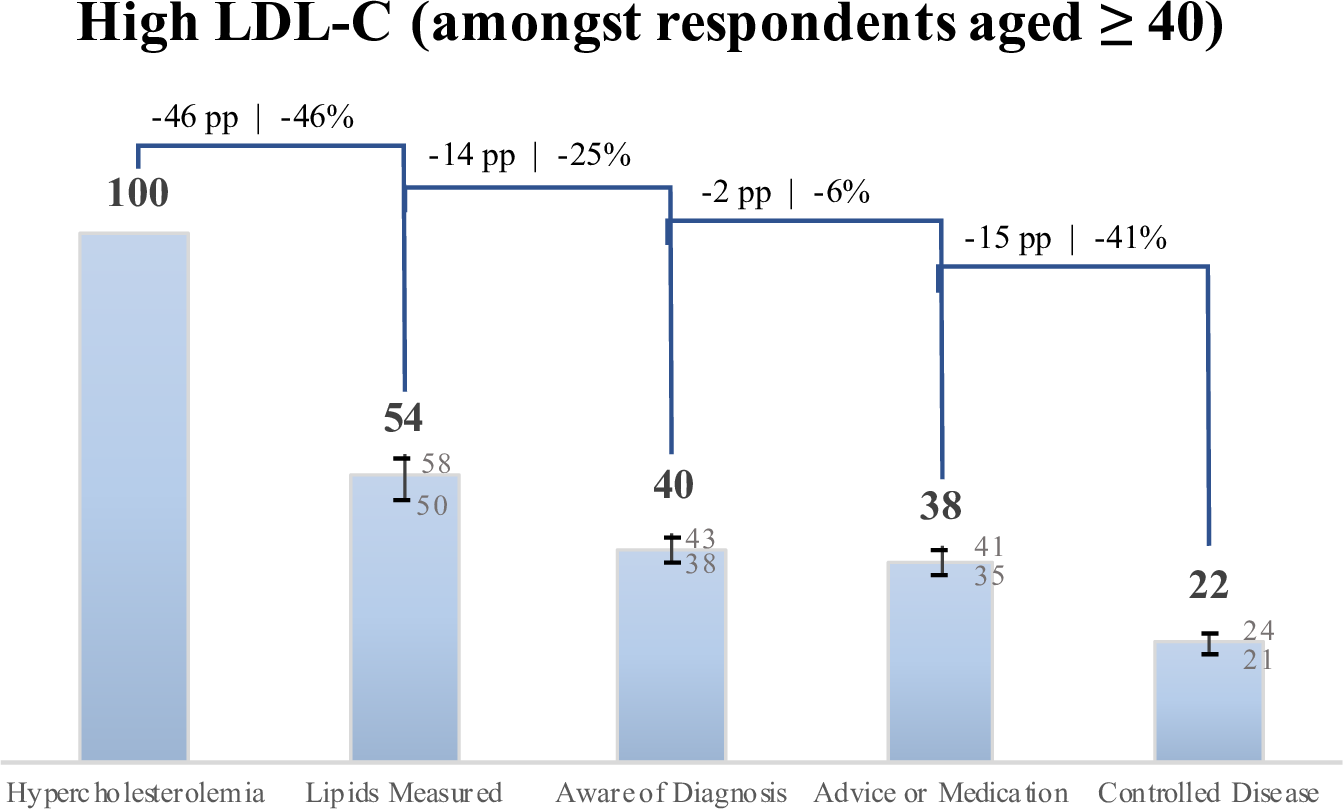


Note: See Note Figure A; Hypercholesterolemia refers to all respondents that are classified as having high LDL-C (≥160 mg/dL) or a self-reported medication status. Cascade restricted to respondents aged 40 or older.

Figure K: Country Fixed Effects in Main Multivariable Poisson Regression Specification


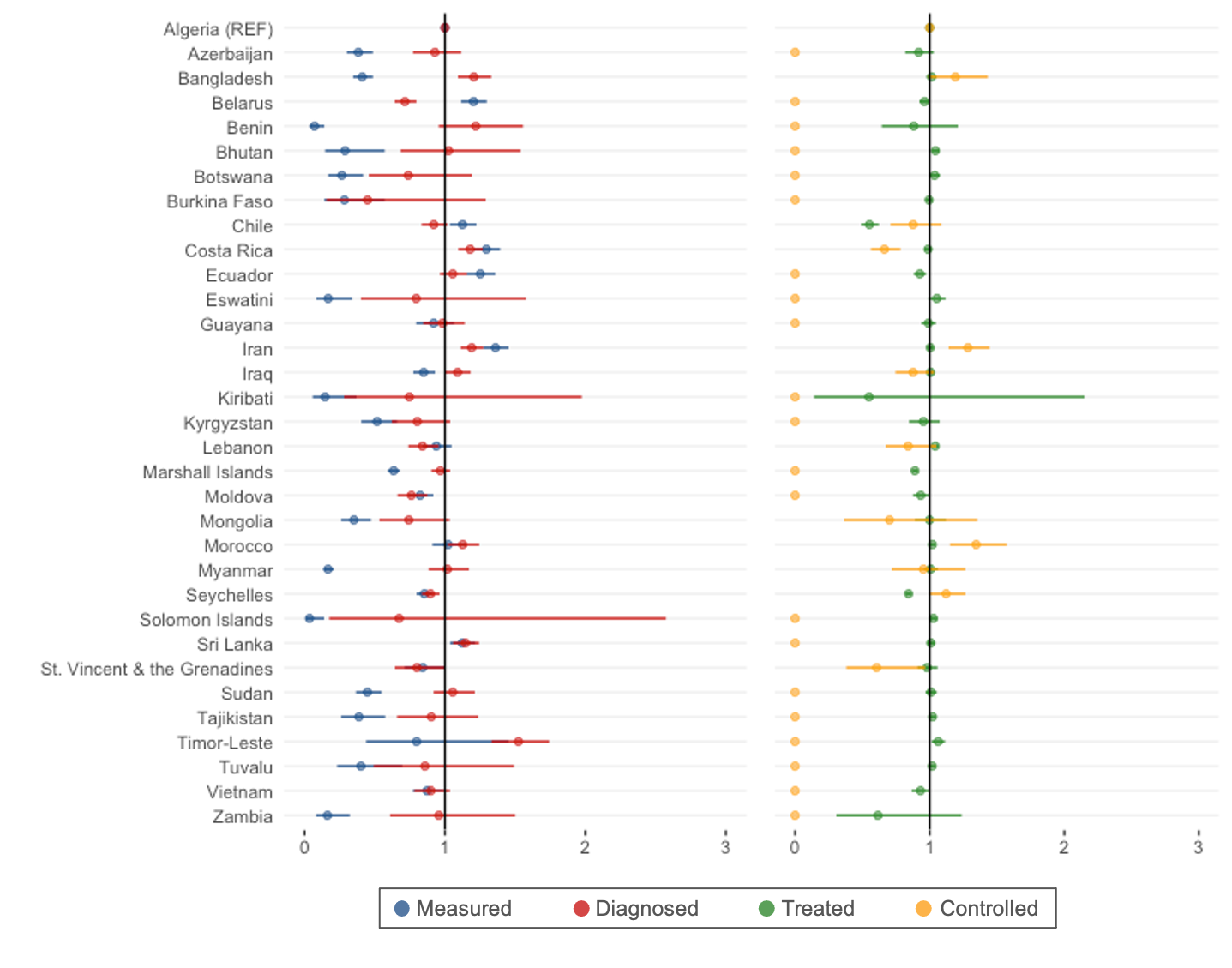


Note: Country fixed effects for multivariable Poisson regression models (Table 2 in main manuscript) with robust error structure, clustering at PSU level, using “Lipids Measured”, “Aware of Diagnosis”, “Advice or Medication”, and “Controlled Disease” as dependent variables, and age, sex, education, smoking, BMI, and comorbid diabetes and hypertension as independent variables. Each cascade stage estimation is conditioned on completion of prior cascade stages. The coefficients indicate risk ratios. The regression samples do not include Tokelau, due to its missing education variable, nor Tonga, due to its missing diabetes variable.

Figure L: Cascade of Care for High TC by Survey Year


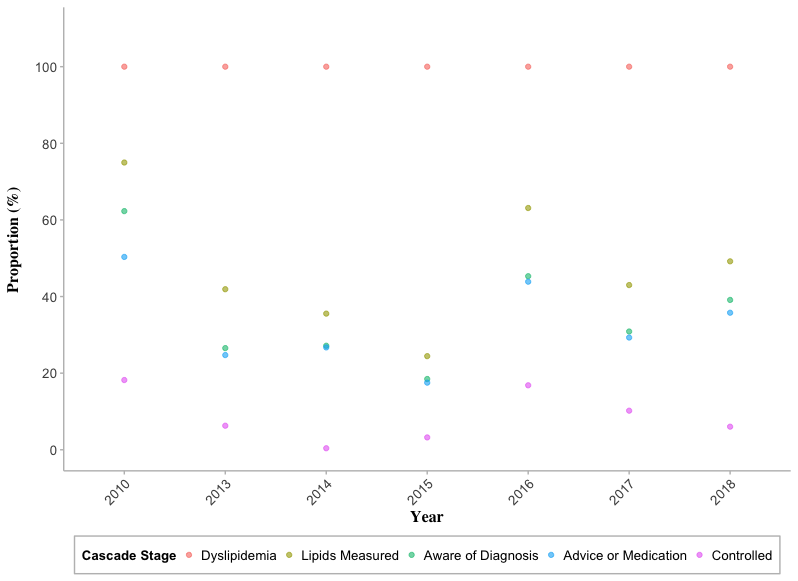


See Note Figure A; Hypercholesterolemia refers to all respondents that are classified as having high TC (≥240 mg/dL) or a self-
