# Supplementary material for: Unmet need for hypercholesterolemia care in 35 low- and middle-income countries: A cross-sectional study of nationally representative surveys
Source: PLoS Med. 2021 Oct 25;18(10):e1003841. doi: 10.1371/journal.pmed.1003841 (PMC8575312; doi:10.1371/journal.pmed.1003841)
Supplement: Attachment — Submitted filename: PLOS Med Dyslipidemia cascade comments.pdf [file pmed.1003841.s011.pdf]

Title: Unmet Need for Dyslipidemia Care in Low- and Middle-Income Countries: A Cross-sectional Study of Nationally-representative Individual-level Data from 35 Countries

This is a large analysis of a combined dataset of 35 STEPS surveys from LMIC. It takes a rather cookie cutter analytic approach to evaluate the proportion of adults with dyslipidemia who have received steps in care towards controlled disease. The strengths of the paper include: large dataset, multiple countries, and use of population-based cohorts (although unclear if all datasets population-based vs. population-representative).

Major limitations:

1. Major concern is this is supposed to reflect an international team reporting LMIC data and yet first, second, third, and last authors all not from the LMIC. Truly not equitable in terms of data sharing, capacity building, and authorship. More, the interpretation of the data seems to lack a perspective from the field—from the LMIC perspective. The data reflects health systems gaps and the primary drop off in the cascade is "diagnosed"—meaning > 50% of those with dyslipidemia did not know they had it b/c they had not had lipids checked. This is likely b/c lipids are not available in their settings for many reasons! Yet authors make the major conclusions in their first paragraph of discussion that the findings are: gaps in treatment and control.
2. Authors overstate the knowledge gap as give many references on dyslipidemia burden of disease including the GBD dataset. Appears principal innovation is applying the care cascade explicitly to data that is already available.
3. Very unclear why authors choose to use adults > 15 years (with 40% of their sample < 35 years), but these are people who would not be expected to have lipids checked or treated with statins?
4. Do not report how many ppts are excluded from the dataset who did not have lipids measured. Is Table 1 denominator truly 100% of each country's STEPS cohort?
5. Do not address how representative their pooled dataset is compared to all LMICs. While they have 35 countries, many countries are quite small population wise and may only make up a small proportion of global burden of dyslipidemia

By section

Introduction

- gap is overstated: authors make two seemingly contradictory points in the introduction: 1) dyslipidemia is widely prevalent in LMIC (cite literature including GBD reports), and 2) "literature has been unable to provide accurate country prevalence estimates due to dearth of nationally representative data"
    - GBD uses WHO Steps data which comprises part of the authors dataset
    - authors overstate their claim that "literature has been unable to provide accurate country prevalence estimates"
        - in fact, multiple GBD publications that the authors cite do exactly this, generate reliable population level estimates

- last sentence first paragraph—statement that high burden of dyslipidemia persist b/c of emphasis on communicable, maternal, and neonatal diseases…. That seems to be speculation. Could be due to lack of MOH funding for lab capacity that includes lipids (usually a send out lab in most LIC) for example. Sets up a narrative that may not have empirical evidence.

Methods

- age for analysis: starting at 15 too young
  - in most countries, unless you had a genetic condition like familial hyperlipidemia, people < 40 yrs with elevated LDLc would not qualify for a statin
  - restricting it to people aged 40 years seems to be more appropriate, especially if authors do not want to avoid evaluating health systems by protocols that are not standard of care
    - they've partly done this by reporting cascade for individuals meeting PEN criteria for lipid screening
- Need clearer definitions of each step—do they include a time period—for example, lipids measured within the last year or within the last 10 years?
- What is the definition of HTN—SBP 130 or 140 for all comers, for those with DM/CRF? Definition of DM also not provided.
- pooled dataset: how representative are these 35 countries of all LMICs (World Bank currently has ~ 79 countries listed as either low income or lower middle income)?
  - Can the authors add a statement about the overall populations of these countries, in relation to the population of all LMICs?
  - can the authors compare their pooled sample with summary statistics from all LMICs?
- page 8: how do point of care tests vs lab tests for lipids typically differ? is one systematically lower/higher than the other? Can this be stated as a short clause in the text, rather than in the appendix?
- Poisson regression: add time fixed effects. 2009-2018 is a decade, could have been secular changes during this time.
- How reliable is fasting documentation in the data?

Discussion

- The main points per the results appear to be overall prevalence of dyslipidemia was ~7% and the major drop using cumulative proportions is lipids measured (43-50%)….the other steps are actually much higher. Yet the discussions starts with the points of treatment and control which seems to be secondary points.
- Moreover, the gap in paragraph 2 seems to be ~ to high income countries which is not given enough discussion as to why.
- Overall the discussion is very long and very unfocused. For example, authors dedicate 1-2 paragraphs to each of screening, diagnosis, treatment, and control. These paragraphs have two components: repeating data from this study (and stating there is heterogeneity), and comparing this to data from other care cascade studies. Because of this structure, there is a lot of repetitiveness in 1) repeating data from study, and 2) repeated comparisons with other countries. Examples below:
      - repeating data from this study:

        - While there was substantial heterogeneity across countries – with screening rates ranging from under 10% in Benin, Kiribati, Myanmar, the Solomon Islands, and Zambia up to 89% in Iran – this stage was consistently found to be the largest or second largest stage of loss along the cascade of care.
        - Again, large heterogeneities across the sample became apparent at this stage, as less than 10% of individuals with dyslipidemia in six countries reached this stage, less than 50% in 23 additional countries, and only six countries were able to retain more than 50% of individuals with dyslipidemia at the diagnosis stage.
        - We found that 29% of those with high TC and 33% of those with high LDL-C had been given advice or medication for their high cholesterol. At the country level, this ranged from less than 5% up to 79%.
        - Both in the pooled analysis and at the disaggregated level, control rates were found to be low, ranging from virtually zero to 27% in all but two countries – Iran (57%) and Morocco (49%).
    - repeated comparisons with other cascade papers
        - According to the US Centers for Disease Control and Prevention, in 2015/16, about 70% of all adults in the United States had received a cholesterol screening test in the past five years, whereas a study on dyslipidemia screening in nine European countries reported much lower rates – with only five countries reaching rates of over 50%, while numerous others fell below 20%
        - Two earlier studies, one from China in 2001 and a second multi-country study that examined data on 40 to 79 year-olds from five high income countries and three middle-income countries over the period 1998-2007, found similarly low levels of diagnosis in Thailand and China.10,3
- Suggestions for stronger discussion:
    - remove repeated presentations of data from this paper.
    - Comparing cascade numbers between countries is not that interesting—what would be more interesting is an explanation of why these numbers differ.
- Strongest paragraph in Discussion is on page 18.

MINOR ISSUES

Introduction

- Ref 3 is from 2018, but text states data is from 2019
- Ref 4 is from 2001 and likely out of date. Can authors find an updated article that makes a similar point?

- Page 7, Cascade Construction: again, ATP III is not the standard of care anymore, and has been superseded in the US by multiple subsequent reports by ACC/AHA. European guidelines have also evolved from Framingham data. References cited as evidence of wide use of ATP guidelines range from analyses published 2013-2014.
        - if authors want to retain ATP III cut offs, should discuss how they compare to most up to date guidelines in Discussion, ie would more or less people meet criteria for dyslipidemia using up to date thresholds
- Ref 8 is from 2010. Can the authors find an updated article that makes a similar point?
- this sentence is confusing, pages 7-8: "Second, as treatment guidelines are usually based on CVD risk scores, rather than on lipid measures alone, and vary across countries, we chose to be conservative in our definition of who is considered to be in need of care as to not evaluate health systems by unapplied care standards"

Methods

- how was missing data handled? requirement for surveys to have at least 50% survey response to be included in pooled data sets, but that still could be a lot of people missing
    - can authors clarify which weights they used from each country's survey weights? Some have multiple depending on which data is examined (interview vs biochemical). This would help with transparency and reproducibility
    - how did authors adjust for missing data? did each country's survey weights adjust for missing data?
- page 7: can authors define cutoff values for the 3 classifications of total cholesterol and 5 classifications for LDLc
- page 11: last paragraph in Statistical Analysis section doesn't seem to belong there. Seems better suited to a Measurements section.

Results

- given 41% sample is < 35 years, should probably report age standardized dyslipidemia prevalence too
- Fig 1: font very small, hard to read
- page 12 type: Easter Mediterranean
- "multivariate Poisson regression" should be "multivariable Poisson regression". See https://www.ncbi.nlm.nih.gov/pmc/articles/PMC3518362/.
- page 14, "less than two out of every five respondents with dyslipidemia had been treated…" but in Fig 1 the treated is 29% for High RC and 33% for High LDLc??
-
