# Supplementary material for: Unmet need for hypercholesterolemia care in 35 low- and middle-income countries: A cross-sectional study of nationally representative surveys
Source: PLoS Med. 2021 Oct 25;18(10):e1003841. doi: 10.1371/journal.pmed.1003841 (PMC8575312; doi:10.1371/journal.pmed.1003841)
Supplement: Attachment — Submitted filename: Revisions_0719.docx [file pmed.1003841.s012.docx]

# Requests from the editors:

1) Please revise your title according to PLOS Medicine's style. Your title must be nondeclarative and not a question. It should begin with main concept if possible.  Please place the study design ("A cross-sectional study”) in the subtitle (ie, after a colon). For example, “Unmet Need for Dyslipidemia Care in 35 Low- and Middle-Income Countries: A Cross-sectional Study.“

Our title now reads: „Unmet Need for Dyslipidemia Care in 35 Low- and Middle-Income Countries: A Cross-sectional Study of Nationally-representative Survey.“

2)	In the abstract Methods and Findings:

a)	Please structure your abstract using the PLOS Medicine headings (Background, Methods and Findings, Conclusions).

b)	Please combine the Methods and Findings sections into one section, “Methods and findings”.

c)	Please ensure that all numbers presented in the abstract are present and identical to numbers presented in the main manuscript text.

d)	Please quantify the main results (with 95% CIs and p values).

e)	Please include the important dependent variables that are adjusted for in the analyses.

f)	In the last sentence of the Abstract Methods and Findings section, please describe the main limitation(s) of the study's methodology.

We have updated the abstract accordingly.

3) Abstract summary - At this stage, we ask that you include a short, non-technical Author Summary of your research to make findings accessible to a wide audience that includes both scientists and non-scientists. The Author Summary should immediately follow the Abstract in your revised manuscript. This text is subject to editorial change and should be distinct from the scientific abstract. Please see our author guidelines for more information: <https://journals.plos.org/plosmedicine/s/revising-your-manuscript#loc-author-summary>.

We have added the Abstract summary.

4)	Your study is observational and therefore causality cannot be inferred. Please remove language that implies causality, such as predictor/ predict. Refer to associations instead.

We have replaced the language surrounding predictors with “correlates” or “individual-level characteristics” throughout the article.

5)	Please add the following statement, or similar, to the Methods: "This study is reported as per the Strengthening the Reporting of Observational Studies in Epidemiology (STROBE) guideline (S1 Checklist)."

We have included the following statement to the methods section (page 12 lines 174-176 – please note that these always refer to the manuscript file without track changes):

“This study is reported as per the Strengthening the Reporting of Observational Studies in Epidemiology (STROBE) guideline (see appendix S1 Checklist).”

6)	Please temper claims of primacy (e.g., “we are the first to apply) by stating, "to our knowledge" or something similar.

We have tempered the respective sections.

7)	In the Methods and Results section:

a)	Please provide 95% CIs and p values for all estimates.

b)	When a p value is given, please specify the statistical test used to determine it.

c)	Please present numerators and denominators for percentages, at least in the Tables

We have implemented this accordingly.

8)	For your Tables and figures, please do the following:

a)	Please indicate in the figure caption the meaning of the bars and whiskers

b)	Please define the abbreviations such as BMI, TC, LDL-C, WHO PEN , PSU, ATP III

We have followed these instructions.

9)	The terms gender and sex are not interchangeable (as discussed in <http://www.who.int/gender/whatisgender/en/> ); please use the appropriate term.

We have replaced all mentions of “gender” with “sex”.

10)	Please replace "subject" with participant, patient, individual, or person.

We have replaced “subject” with “study participant”

11)	Please replace “diabetics” with “persons with diabetes.” This applies also to similar terms like hypertensives

We have replaced “diabetics” (and similar) with “having diabetes” (and similar).

12)	Please use the "Vancouver" style for reference formatting and see our website for other reference guidelines. For example, six names should appear before et al. <https://journals.plos.org/plosmedicine/s/submission-guidelines#loc-references>. Please ensure that weblinks are current and accessible to date.

We are now using the Vancouver style for reference formatting.

13)	Please include line numbers in the next revision

We are now including line numbers.

# Reviewer #1:

The study examines the cascade of dyslipidemia care for people in 35 LMICs. I think this is an important study in that it fills the evidence gap of the unmet need of dyslipidemia care in LIMICs.

Overall, this is a well-designed and carefully conducted study. However, I think there are still a few major issues that the authors need to address.

First, although you noted that there are some systematic differences between the surveys (e.g., the level of blood lipids is measured differently across surveys), you assumed the data across surveys are comparable and did not adjust for the potential bias due to different sources. There are in fact approaches you could use to adjust this bias. An example could be the "bias correction" in this appendix of a Lancet study.

(<https://www.thelancet.com/cms/10.1016/S0140-6736(17)31833-0/attachment/d006d5eb-f74d-4fe3-a25f-2035410cfab3/mmc1.pdf>)

We thank you for this important comment. Out of all 35 surveys included in our analysis, 32 are based on the WHO Stepwise Approach to Surveillance of Noncommunicable Disease (NCD) Risk Factors (STEPS). The STEPS datasets follow a standardized questionnaire and survey protocol. The three additional studies included are also very similar to the STEPS surveys in these dimensions. Because of this, we believe the bias correction as is applied in the Global Burden of Disease study cited above is generally not as meaningful in the context of our analysis. In order to address varying survey methodology, we account for survey design in our estimates as well as report all survey design reports for maximum transparency.

You have furthermore rightfully pointed out that the surveys include two different biomarker measurements, which may potentially increase the uncertainty in our estimates. Literature shows that point-of-care-machines compare well against laboratory testing and can be reliably used for lipid screening [1–3]. One study by Ferreira et al. (2015) specifically compares the clinical agreement of the CardioCheck PA – which is used by the majority of countries – to the laboratory in sorting lipid records into the ATP III lipid classifications used in our manuscript [1]. They showed 94.6%-97.7% agreement depending on the biomarker measured. Therefore, we do not suspect a systematic bias in our analysis based on the different lipid measuring methods. Nevertheless, a bias could potentially arise from the fact that point-of-care machines usually have an upper cut-off above which lipids cannot be reliably measured. Because of this we are restricting our analysis to the range that these devices are equipped to report on (see Appendix 4). We also provide a supplemental analysis, in which we do not restrict on this, which can be seen in Appendix 7. If needed, we are happy to also provide disaggregated cascade analyses based on lipid measurement. However, as laboratory measurements are more expensive, we would argue that a disaggregation would likely speak more to the selection of countries that can afford laboratory testing rather than a bias in the measures themselves.

To make this point clearer in our manuscript as well, we have amended the discussion as follows (page 26 lines 430-435):

“In addition, our definition of hypercholesterolemia was based on biomarkers that, in some countries, were measured with point-of-care devices. While these may be less accurate than lab-based testing, studies have shown that they can be reliably used for lipid screening [29,58–60]. A study by Ferreira et al. (2015) found a 94.6%-97.7% agreement between the CardioCheck PA – which is used by the majority of countries – and the laboratory when sorting lipid records into the ATP III lipid classifications used in our analysis [60].”

Second, I believe it should be "modified Poisson regression" instead of "Poisson regression" that you used to examine the predictors of cascade progression. Poisson regression is mostly used for count outcomes while modified Poisson regression can be used on binary outcomes (<https://academic.oup.com/aje/article/159/7/702/71883>). However, I think modified Poisson is usually used for rare events and I don't think your outcomes are "rare". You might want to check the validity and justify the use of this regression for your analyses. Also, it would be better if you could provide a mathematical equation for the regression(s) in the paper.

Lastly, if you chose modified Poisson because you wanted RR instead of OR, I personally think probability is a more intuitive measure for common readers. If you use logistic regression, which is more common than modified Poisson for binary outcomes, you can predict probability from the fitted logistic regressions and can compare the probability of cascade progression with different values of covariates.  Here is a package you could use to do that. (<https://faculty.washington.edu/cadolph/?page=60>)

Thank you for this valuable comment. You are correct that we are using modified Poisson regression as described in Zou (2004). We chose this model specification for ease of interpretation, such that we can display risk ratios rather than odds ratios as is frequently done in the literature in cases of non-rare, binary outcomes.[4–6] We also included a linear probability model in our appendix, which will cater to readers, such as yourself, who find probability the more intuitive measure. We opted against using logistic regression as our main specification, because this specification can only approximate risk ratios in analyses of rare outcomes, as you have rightly pointed out.

We have changed our terminology from “Poisson regression model [yielding risk ratios]” to “modified Poisson regression model” throughout the manuscript to further clarify our model specification. Furthermore, we have included the mathematical equations to our supplementary material.

Here are some specific comments about the manuscript.

1. Please provide line numbers for the manuscript in the future.

We are now including line numbers.

2. Page 6, Method/Data sources: The IHME's GHDx could be a great source for searching of survey data.

Thank you for this suggestion. IHME’s GHDx data resources were already included in our search. We have updated the appendix to provide more detail on our search strategy (see Appendix 1).

3. Page 8, Method/ Cascade Construction: your definition of dyslipidemia includes people who were taking lipid-lowering medication. However, why did you exclude all "individuals without a biomarker record form the analysis"? What if these individuals do have record of taking lipid-lowering medication? It's not very clear here.

Thank you for pointing out this lack of clarity. As you noted, we define dyslipidemia as either an abnormally high lipid biomarker and/or self-reported medication use. Our analysis excludes individuals that self-reportedly take medication, but for whom we do not however have a biomarker measurement – for instance in cases where the respondent refused the lipid measurement. In total, this pertains to 1209 respondents from 30 countries. We had originally decided to exclude these individuals, because we have no information on which biomarker (i.e. TC or LDL-C) falls within abnormal ranges and therefore cannot consistently sort these respondents into our analysis. Furthermore, given the paucity of hypercholesterolemia care data in the existing literature and therefore missing points of comparisons, we also chose not to impute any values, such that we do not introduce potentially flawed hypothesized relationships. However, we have now added a cascade analysis that includes these individuals based on their self-reported medication status only to our supplementary material (see graph below) and clarified this in our manuscript:

Main manuscript (p.10 lines 101-104)

“Individuals without a biomarker record were excluded from the analysis (Appendix 4). A sensitivity analysis that includes individuals with no biomarker measurement, in which hypercholesterolemia is defined purely based on the self-reported medication status, can be found in Appendix 7.”

Appendix 4 of supplementary material (p.29 lines 1552-1562)

“High total cholesterol

An individual was classified as having high TC if their level was above 6.21 mmol/L (according to ATP III guidelines) or if they reported taking medication for high cholesterol [4]. If individuals had only a self-reported medication status and no TC measurement, they were excluded from the analysis. A sensitivity analysis that includes 1209 additional individuals with no biomarker measurement and which defines high TC purely based on the respondent’s self-reported medication status can be found in Appendix 7.

High LDL cholesterol

An individual was classified as having high levels of LDL-C if their level was above 4.14 mmol/L (according to ATP III guidelines) or if they reported taking medication for high cholesterol. If individuals had only a self-reported medication status and no LDL-C measurement, they were excluded from the analysis.”

Appendix 7 of supplementary material:

Figure 10: Cascade of Care of high TC Including Respondents with Missing Biomarker

See note Figure 1; Hypercholesterolemia refers to all respondents that are classified as having high TC ($\geq$240 mg/dL) or a self-reported medication status. Also includes respondents for whom no TC measure was available. In those cases hypercholesterolemia is based on the self-reported medication status only.

4. Page 11, Results/ Sample Characteristics: "…Secondary schooling or higher education was completed by 41% of those with high TC and 31% of those with high LDL-C. Around 59-61% of those with dyslipidemia were overweight or obese and approximately 16-17% were current smokers…" Comparing the summaries with those among people without dyslipidemia could be more helpful.

We thank you for this suggestion. We have added the sample characteristics for respondents without dyslipidemia, i.e. those with normal TC and LDL-C ranges and no self-reported lipid-lowering medication use, to the appendix and added the following sentence to our main manuscript (p.12 lines 192-194):

“Sample characteristics of respondents, who had TC and LDL-C measures in normal ranges and who reported not taking lipid-lowering medication can be found in Appendix 5.”

5.	Page 28, Table 2: There should be one overall p-value for the categorical variables.

Thank you for this suggestion. We have added an analysis of deviance for all our main regression specifications to the appendix detailing the reduction in residuals of all explanatory variables included.

Figure 12: Analysis of Deviance for Main Modified Poisson Regression Specifications (Table 2 Main Manuscript)

**Reviewer #2:**

Major Comments

I greatly appreciate the authors' work to put together this information for furthering global dyslipidemia care. This data was strikingly lacking and the current work contributes a step forward. I have few suggestions below and I hope the authors find this helpful.

1. This sentence from the abstract is not clear. "The cascade analysis showed that 43% (95% CI: 40 to 45%) of subjects with high TC and 47% (95% CI: 44 to 50%) with high LDL-C had ever had their cholesterol measured." A corollary of this is - 57% of subjects with high TC and 53% with high LDL-C had not ever had their cholesterol measured. How is it possible for someone to have high TC or LDL-C, but not ever have cholesterol measured?

Thank you for this important comment. The categorization into high TC and high LDL is based on lipid measures that were taken as part of the respective survey. The “lipids measured” cascade stage refers to lipid measurements that had taken place *prior to the survey, based on the self-report of the participant regarding medical care they had received*. While we describe this in the methods section of our manuscript, we appreciate your comment and have further specified this throughout our manuscript to prevent further misunderstandings.

2. Related to comment 1 is Figure 1 (and other similar figures): How is it possible to have dyslipidemia but lipids not measured? Is the difference solely from self-reported medication use? Even so, I can't imagine someone prescribing medication without checking lipids at some point. This actually makes many parts of the manuscript difficult to follow. Most likely, the respondents were not aware of lipids being measured previously. Please clarify this. Perhaps best approach would be remove the part "lipids measured" altogether from the cascade and change accordingly throughout the manuscript.

Please see response to comment 1

3. Has the cascade care methodology and performance been previously used in hypercholesterolemia or other chronic diseases? Can you please discuss something about its overall validity (at least, face validity) in the methods section?

As detailed previously in the literature, “The cascade of care approach has been widely used to monitor performance and to examine response to the HIV epidemic and progress toward coverage goals for populations affected by HIV [9,10]. This method uses a quantitative depiction of the stepwise care system for the population affected by a disease of interest, typically involving testing, diagnosis, treatment initiation, adherence to treatment, and effective control. The primary strength of cascade of care analysis is the ability to identify where in the continuum of care the greatest losses to care occur [9]. This in turn can help facilitate effective, evidence-based targeting of programs and policies to address these gaps. This analytic approach is being increasingly applied at the national level to examine management of chronic diseases such as hypertension and diabetes [11–15].” [7]

To this point, we have added the following to our manuscript (pages 6-7 lines 20-27):

“Our analysis aims to address this dearth of evidence by identifying the unmet need for hypercholesterolemia care using a pooled dataset of nationally representative, population-based surveys that includes 129,040 individuals from 35 LMICs. We assess the unmet need for care by applying the cascade of care approach, a quantitative depiction of the screening, diagnosis, treatment, and control stages within the care system of the affected population groups. This methodology has been widely used to monitor care responses to the HIV epidemic and is increasingly applied to examine the management of chronic diseases, such as diabetes or hypertension [14–17].”

Minor Comments

1. "Normal" cholesterol level an evolving concept and is complex. For example, LDL-C of 130 as controlled dyslipidemia is fairly elevated from the current US, European and Canadian perspectives. Nevertheless, the cutoffs used in the current analysis is a conservative estimate, and should be acknowledged in the limitation section.

Thank you for pointing this out. We agree that a better discussion of the various guidelines adds important context to our manuscript. Our supplementary appendix now includes a consideration of alternative cutoffs for defining hypercholesterolemia, such as those proposed by the AHA/ACC guidelines. We have also amended our manuscript to further acknowledge this.

Main Manuscript:

Methods (p.9 lines 83-86):

“ However, in supplemental analyses, we redefine hypercholesterolemia to further include borderline high values as well as apply a definition based on the American College of Cardiology / American Heart Association Task Force on Clinical Practice Guidelines (AHA/ACC) […]

^2^ AHA/ACC uses an LDL-C cutoff of 70 mg/dL as a threshold for statin therapy in adults 40 to 75 years of age with diabetes or with a 10-year atheroslerotic cardiovascular disease risk of over 7.5%. In our definition, we classify everyone with an LDL-C measurement of 70 mg/dL or higher as having hypercholesterolemia.”

Main Manuscript: Discussion (page 27 lines 445-456):

“Finally, we chose to define hypercholesterolemia and achieving control based on the ATP III guidelines due to their frequent use in the literature. However, these are relatively conservative in comparison to some national guidelines, as is apparent when examining the markedly lower cascade performance when applying AHA/ACC guidelines (see Appendix 7). The comparability of countries is generally limited by a lack of universally used guidelines, as different guidelines are applied across countries and clinical settings and may even include geographical parameters, as is the case in the European Society of Cardiology (ESC) and European Atherosclerosis Society (EAS) guidelines [61,62]. Despite this, such a comparison still offers important insights into national and global care gaps and can be used for identifying effective policy as well as serve as markers of progress in tackling the burden of hypercholesterolemia.”

2. I would replace "dyslipidemia" on the title and throughout the manuscript by "hypercholesterolemia" since the latter is more specific to cholesterol than the former (dyslipidemia also implies markers beyond cholesterol, such as triglycerides).

We replaced “dyslipidemia” with “hypercholesterolemia” throughout the manuscript.

3. Cascade and its performance is a jargon and should be described at its first appearance in the abstract and manuscript. The terminology first appears in the results section of the abstract and the introduction section of the manuscript, but the description is seen only in the methods section of the manuscript.

We thank you for pointing this out and have now included a brief description of the cascade of care approach in the introduction (also see response to major comment 3, pages 6-7 lines 20-27):

“Our analysis aims to address this dearth of evidence by identifying the unmet need for hypercholesterolemia care using a pooled dataset of nationally representative, population-based surveys that includes 129,040 individuals from 35 LMICs. We assess the unmet need for care by applying the cascade of care approach, a quantitative depiction of the screening, diagnosis, treatment, and control stages within the care system of the affected population groups. This methodology has been widely used to monitor care responses to the HIV epidemic and is increasingly applied to examine the management of chronic diseases, such as diabetes or hypertension [14–17].”

4. I understand the difficulty in simplifying the complex data, but in Table 1 for the total cholesterol sample, why 7% of 128998 (=9029) is not the number of observations on the sample with high TC (N=10737)? Same comment for Table 4 in the appendix. Is it because the latter also includes people taking lipid lowering medications? If so, adding a clarifying sentence at the footnote will help the readers. Ditto question for LDL-C sample.

In these tables, percentages are estimated taking into account sampling design and survey weights, whereas the number of observations is unweighted. This is marked as such in respective table and described in the tables’ footnotes. We chose this format in order to inform the reader on the number of observations on which we are basing our estimates and at the same time to appropriately reflect population prevalences.

5. Figure 1 footnote reads: "Lipids Measured refers to the percentage share of all sick respondents (classified based on relevant biomarker) that have ever had their lipid status measured as per self-reported information." Sick respondents seem like the participants were severely ill, which is not necessarily the case in people with dyslipidemia. Please correct throughout the manuscript.

We replaced “sick” with the respective, appropriate phrasing “having hypercholesterolemia” or “respondents with hypercholesterolemia”.

6. Not all advice given are remembered by patients, particularly lifestyle modification related advice. Thus, even though lifestyle changes were advised, participants may not remember them later. Please acknowledge.

We have now added a note on this in the discussion of our limitations (page 26 lines 424-430):

“This study had several limitations. First, several measures may be subject to measurement biases. For one, our data on health services received was self-reported and thus may be subjected to a recall bias. For instance, individuals that were taking medication could have been more likely to remember ever being screened for hypercholesterolemia, affecting the absolute probability of reaching each cascade stage. Similarly, recalling the provision of low-touch treatment interventions, such as having received lifestyle advice, may be difficult for respondents.”

7. Based on Table 3 similar proportion of people received medication and advice (Table 3, Appendix). However, it's unclear what proportion of people with high cholesterol only received advice without medications. This can be helpful information as unfortunately despite being generic, these medications may not be within reach of a large number of population.

We thank you for suggesting this additional analysis. We have added the proportion of people that received only lifestyle advice or only medication to the existing table in the Appendix. As can be seen there, a majority of respondents receive both lifestyle advice and medication.

Table 5: Cascade of Care Disaggregated for Medication and Lifestyle Advice Stage

|  | High TC | | High LDL-C | |
| --- | --- | --- | --- | --- |
|  | Percent | 95% CI | Percent | 95% CI |
| Hypercholesterolemia | 100 |  | 100 |  |
| Lipids Measured | 43 | [40 , 45] | 47 | [44 , 50] |
| Aware of Diagnosis | 31 | [29 , 33] | 36 | [33 , 38] |
| Medication | 24 | [22 , 26] | 29 | [27 , 32] |
| Controlled Disease | 7 | [6 , 8] | 19 | [18 , 21] |
| Advice | 25 | [23 , 27] | 26 | [24 , 28] |
| Controlled Disease | 6 | [5 , 7] | 15 | [14 , 17] |
| Only Medication | 5 | [3 , 7] | 7 | [6 , 8] |
| Controlled Disease | 1 | [1 , 2] | 4 | [3 , 5] |
| Only Advice | 5 | [4 , 6] | 4 | [3 , 5] |
| Controlled Disease | 0 | [0 , 0] | 0 | [0 , 0] |

Note: See Figure 2; Hypercholesterolemia refers to all respondents that are classified as having high TC (≥240 mg/dL) / high LDL-C (≥160 mg/dL) or a self-reported medication status. Medication refers to those that have received medication for their high cholesterol (independent of whether lifestyle advice was also received) and advice refers to those that have received lifestyle advice (independent of whether medication was also received). Only Medication refers to having received medication, but not lifestyle advice. Only advice refers to having received lifestyle advice, but not medication. Controlled Disease considers those respondents that have TC and LDL-C values within the range considered normal by ATP III guidelines. All cascade stages are restricted on reaching the prior cascade stages.

8. First the number of people with lipids measured in low income countries is small, and not surprisingly the cascade performance is poorest for low income countries (Table 5, appendix). Can the authors comment something about this in the discussion? The method of lipids measurement for 19 countries was based on point of care devices. One of the important barriers for dyslipidemia care worldwide is lack of access to lipid testing, which is needed more than once in many people. Perhaps using the POC device can fill this gap (within the constraints of its limitation). Making all statin lipid lowering therapy as an essential medication (of WHO) is a step in correct direction (currently only simvastatin is listed, which is one of the medication that we least likely to prescribe) and making them available in remote areas of all countries, including low income countries can partly address this gap. These elements could be discussed within the constructs of high performing countries. See last minor comment.

We thank you for your suggestions. We have reworked the discussion and included points on the low care performance in low income countries, the potential of point of care devices in increasing screening rates, as well as the role of statins lipid lowering therapy as an essential medication.

9. I am not following what the authors are saying in the text in the discussion section (underlined part). Please clarify or if of minor importance, remove. "This suggest that the targeting of care to people with high CVD risk, as described above for the awareness stage, also was preserved in later cascade stages - in the case of diabetes this included even the controlled stage. While recognizing that access to care remains low at the awareness stage, this is nevertheless an encouraging finding given the markedly worse CVD outcomes of patients with diabetes in comparison to those without."

We have rephrased it to the following (p.23 lines 350-357):

“Our results further showed that age, high BMI, having diabetes, and having hypertension were significantly associated with being aware of one’s high cholesterol level. This suggests that health care workers may appropriately prioritize those at greatest risk of CVD across the cascade, not only at the screening stage as described above. In the case of diabetes, this significant effect persisted through the final “control” stage of the cascade of care. This is an encouraging finding given the markedly worse CVD outcomes of patients with diabetes in comparison to those without [41].”

10. An average reader would have difficulty understanding this conclusion sentence. "We found poor cascade performance for dyslipidemia care in this group of LMICs, with large losses to care occurring at each stage of the cascade." I recommend rather describe what actually was seen - e.g., only a smaller percentage of those aware of diagnosis had cholesterol level controlled - something similar.

We have rephrased it to the following (p.27 lines 459-460):

“We found low levels of access to hypercholesterolemia care in this group of LMICs, with especially large levels of unmet screening and control needs across all countries.”

11.	I agree about highlighting the constructs of better performing countries, such as Sri Lanka for better cholesterol control. In addition to the conclusion, perhaps the authors can highlight this in their discussion section.

Our manuscript includes a discussion of the better performing countries (p.27 lines 461-464) and we have amended the conclusion to read the following:

“A closer examination of the better performing countries in our study – such as Sri Lanka, Costa Rica, Iran, and Morocco – could yield important policy lessons, especially as the lipid cascade offers a potentially important tracer of unmet need for chronic disease care.”

# Reviewer #3:

The present work is interesting and timely, since it affords the growth of dyslipidemias in Low and Middle Income Countries and the current unmet needs.

The study analyzes information from 35 Countries and is based on a sufficient sample size to draw conclusions (almost 130,000 cases). I have a few points to raise. The authors are invited to address them in revising the manuscript.

Most of my concerns, indeed, were already identified by the authors and listed in the "limitations" section.

However, I believe they deserve a more thorough discussion, mostly in view of the validity of the experimental approach and the interpretation of the results.

1. Dyslipidemia was assessed as a TC level exceeding 240 mg/dl and an LDL-C exceeding 160 mg/dl using ATP III. This represents today quite an obsolete way to define hypercholesterolemia and it should be replaced by a more contemporary, guideline-based definition, to avoid or minimize the risk of losing many subject which are indeed hypercholesterolemic and may deserve life style or pharmacologic interventions. (by the way, some of  the Refs quoted in this regard 23-27 are not totally appropriate). Keeping in mind the latest widely accepted relationship between cholesterol and cv events (which should be mentioned), the thresholds selected in this analysis are well beyond levels considered unharmful

We thank you for this important suggestion. We agree that a better discussion of the various guidelines adds important context to our manuscript. Our supplementary appendix now includes a consideration of alternative cutoffs for defining hypercholesterolemia, such as those proposed by the AHA/ACC guidelines. We have also amended our manuscript to further acknowledge this.

Main Manuscript:

Methods (p.9 lines 83-86):

“ However, in supplemental analyses, we redefine hypercholesterolemia to further include borderline high values as well as apply a definition based on the American College of Cardiology / American Heart Association Task Force on Clinical Practice Guidelines (AHA/ACC) […]

^2^ AHA/ACC uses an LDL-C cutoff of 70 mg/dL as a threshold for statin therapy in adults 40 to 75 years of age with diabetes or with a 10-year atheroslerotic cardiovascular disease risk of over 7.5%. In our definition, we classify everyone with an LDL-C measurement of 70 mg/dL or higher as having hypercholesterolemia.”

Main Manuscript: Discussion (page 27 lines 445-456):

“Finally, we chose to define hypercholesterolemia and achieving control based on the ATP III guidelines due to their frequent use in the literature. However, these are relatively conservative in comparison to some national guidelines, as is apparent when examining the markedly lower cascade performance when applying AHA/ACC guidelines (see Appendix 7). The comparability of countries is generally limited by a lack of universally used guidelines, as different guidelines are applied across countries and clinical settings and may even include geographical parameters, as is the case in the European Society of Cardiology (ESC) and European Atherosclerosis Society (EAS) guidelines [61,62]. Despite this, such a comparison still offers important insights into national and global care gaps and can be used for identifying effective policy as well as serve as markers of progress in tackling the burden of hypercholesterolemia.”

2. The source of information for lipid levels is extremely heterogeneous (self-reported, point-of-care, lab-based testing). It should be made an additional effort to provide a sub-analysis based on the different sources. This process, if guided by the authors, would most likely put the reader in a better shape than admitting that "our disaggregated cascade analysis should be followed with caveats in mind" (page 19), including also the very heterogeneous sample size due to specific country-limitation.

Thank you for this valuable suggestion. Literature shows that point-of-care-machines compare well against laboratory testing and can be reliably used for lipid screening [1–3]. One study by Ferreira et al. (2015) specifically compares the clinical agreement of the CardioCheck PA – which is used by the majority of countries – to the laboratory in sorting lipid records into the ATP III lipid classifications used in our manuscript [1]. They showed 94.6%-97.7% agreement depending on the biomarker measured. Therefore, we do not suspect a systematic bias in our analysis based on the different lipid measuring methods. With this in mind, we would like to point out that a bias could potentially arise from the fact that point-of-care machines usually have an upper cut-off above which lipids cannot be reliably measured. Because of this we are restricting our analysis to the range that these devices are equipped to report on (see Appendix 4). We also provide a supplemental analysis, in which we do not restrict on this, which can be seen in Appendix 7. If needed, we are happy to also provide disaggregated cascade analyses based on lipid measurement. However, as laboratory measurements are more expensive, we would argue that a disaggregation would likely speak more to the selection of countries that can afford laboratory testing rather than a bias in the measures themselves.

To make this point clearer in our manuscript as well, we have amended the discussion as follows (page 26 lines 430-435):

“In addition, our definition of hypercholesterolemia was based on biomarkers that, in some countries, were measured with point-of-care devices. While these may be less accurate than lab-based testing, studies have shown that they can be reliably used for lipid screening [29,58–60]. A study by Ferreira et al. (2015) found a 94.6%-97.7% agreement between the CardioCheck PA – which is used by the majority of countries – and the laboratory when sorting lipid records into the ATP III lipid classifications used in our analysis [60].”

3. Discussion is very long and could be reduced in size. Despite that, it omits any comparison with high income countries, which in my view would be very informative  and may add value to the study

As per your suggestion, we have reworked the discussion to make it more concise.

4. Definition of Low-Middle income countries should be provided and supported by some informative data

We use the World Bank income classification of the respective survey year when categorizing countries into low income, lower-middle income, and upper-middle income. Table 2 of our supplementary appendix shows which country falls under each category.

5. Figures are nice. How about introducing one comparing with high-income countries? By the way data from the last Euro Aspire, for Instance, are not really satisfactory in terms of Cholesterol control. Achievements of satisfactory cholesterol control are still surprisingly low.

We thank you for your comment and agree that a comparison with high income countries would yield further interesting insights. While we do not have ready-to-use data available for such a task at this point, we are planning for such analyses in the future. In the meantime, we discuss how the met screening needs in LMICs compare to those in high-income countries based on existing evidence.

# Reviewer #4:

This is a large analysis of a combined dataset of 35 STEPS surveys from LMIC.  It takes a rather cascade analytic approach to evaluate the proportion of adults with dyslipidemia who have received steps in care towards controlled disease.  The strengths of the paper include: large dataset, multiple countries, and use of population-based cohorts (although unclear if all datasets population-based vs. population-representative).

Major limitations:

1. Major concern is this is supposed to reflect an international team reporting LMIC data and yet first, second, third, and last authors all not from the LMIC.  This is not equitable in terms of modern data sharing, capacity building, and authorship.  More, the interpretation of the data seems to lack a perspective from the field—from the LMIC perspective.  The data reflects health systems gaps and the primary drop off in the cascade is "lipids measured"—meaning > 50% of those with dyslipidemia did not know they had it b/c they had not had lipids checked.  This is likely b/c lipids are not available in their settings for many reasons!  Yet authors make the major conclusions in their first paragraph of discussion that the findings are: gaps in treatment and control.

We thank the reviewer for raising this very important point. We agree to the fullest extent that global health research needs to be more equitable in all research outputs. This is also reflected in our team’s portfolio of publications and we continuously strive to further improve on this. The order of authorship on this particular manuscript adequately reflects the contributions made by its coauthor team and includes perspectives shared by in-country coauthors. We further thank the reviewer for suggesting a refocused conclusion. We have reworked the discussion and included a stronger focus on the gaps in lipid testing.

2. Authors overstate the knowledge gap as give many references on dyslipidemia burden of disease including the GBD dataset.  Appears principal innovation is applying the care cascade explicitly to data that is already available.

We highly value the insights that major references, such as the Global Burden of Disease and PURE studies, are providing on the burden of hypercholesterolemia. We argue that our study complements the existing literature by providing access to care needs based on individual-level, nationally representative data in addition to hypercholesterolemia prevalences.

3. Very unclear why authors choose to use adults > 15 years (with 40% of their sample < 35 years), but these are people who would not be expected to have lipids checked or treated with statins?
   - in most countries, unless you had a genetic condition like familial hyperlipidemia, people < 40 yrs with elevated LDLc would not qualify for a statin
   - restricting it to people aged 40 years seems to be more appropriate, especially if authors do not want to avoid evaluating health systems by protocols that are not standard of care
   - they’ve partly done this by reporting cascade for individuals meeting PEN criteria for lipid screening

We thank you for raising these points. We believe there are two important dimensions to consider when assessing access to care. The first is whether the health system is able to appropriately target and treat those at greatest risk for hypercholesterolemia, as you have pointed out. In addition to that, it is also important to understand how many individuals with hypercholesterolemia are missed by the health system, independent of their other risk factors – especially considering that health services such as lifestyle advice should also be provided to younger cohorts. Because of that we believe in the value of also showing the cascade of care estimates based on the full age range available in the surveys. In order to further cater to your and potential other readers’ interest of access to care for high-risk groups, we have added a cascade of care analysis restricted to individuals aged 40 or higher (in addition to the cascade analysis of respondents meeting WHO PEN screening criteria, which also focuses on respondents over the age of 40 amongst other criteria and which was already part of the manuscript).

Please find the following addition in our manuscript and supplementary material:

Manuscript (p.17 lines 240-241):

“The cascade of care restricted to respondents aged 40 or older mirrors the cascade results for individuals meeting PEN criteria for lipid screening.”

Appendix 7:

4. Do not report how many ppts are excluded from the dataset who did not have lipids measured.  Is Table 1 denominator truly 100% of each country's STEPS cohort?

We assume that you are referring to the “Overall sample” columns of Table 1 in your comment. Here, the denominator is not 100% of all countries’ STEP cohorts, but rather the sample population with a valid TC or LDL-C biomarker measurement. As we noted in our response to Reviewer 1, our analysis excludes individuals that self-reportedly take medication, but for whom we do not however have a valid biomarker measurement – for instance in cases where the respondent refused the lipid measurement. In total, this pertains to 1209 respondents from 30 countries. We had originally decided to exclude these individuals, because we have no information on which biomarker (i.e. TC or LDL-C) falls within abnormal ranges and therefore cannot consistently sort these respondents into our analysis. Furthermore, given the paucity of hypercholesterolemia care data in the existing literature and therefore missing points of comparisons, we also chose not to impute any values, such that we do not introduce potentially flawed hypothesized relationships. However, we have now added a cascade analysis that includes these individuals based on their self-reported medication status only to our supplementary material (see graph below) and clarified this in our manuscript:

Main manuscript (p.10 lines 101-104)

“Individuals without a biomarker record were excluded from the analysis (Appendix 4). A sensitivity analysis that includes individuals with no biomarker measurement, in which hypercholesterolemia is defined purely based on the self-reported medication status, can be found in Appendix 7.”

Appendix 4 of supplementary material (p.29 lines 1552-1562)

“High total cholesterol

An individual was classified as having high TC if their level was above 6.21 mmol/L (according to ATP III guidelines) or if they reported taking medication for high cholesterol [4]. If individuals had only a self-reported medication status and no TC measurement, they were excluded from the analysis. A sensitivity analysis that includes 1209 additional individuals with no biomarker measurement and which defines high TC purely based on the respondent’s self-reported medication status can be found in Appendix 7.

High LDL cholesterol

An individual was classified as having high levels of LDL-C if their level was above 4.14 mmol/L (according to ATP III guidelines) or if they reported taking medication for high cholesterol. If individuals had only a self-reported medication status and no LDL-C measurement, they were excluded from the analysis.”

Appendix 7 of supplementary material:

Figure 10: Cascade of Care of high TC Including Respondents with Missing Biomarker

See note Figure 1; Hypercholesterolemia refers to all respondents that are classified as having high TC ($\geq$240 mg/dL) or a self-reported medication status. Also includes respondents for whom no TC measure was available. In those cases hypercholesterolemia is based on the self-reported medication status only.

5. Do not address how representative their pooled dataset is compared to all LMICs. While they have 35 countries, many countries are quite small population wise and may only make up a small proportion of global burden of dyslipidemia

Our sample comprises around 11.2% of the low- and middle-income population in 2015. Throughout our manuscript we pay great attention to state that our results only speak to this particular set of countries and not all LMICs by tempering results with the phrasing “in this set of LMICs” and the like. We do hope that the number of LMICs collecting data on access to hypercholesterolemia care increases in the future such that a more representative picture can be drawn.

6. Do not address missing data in general.

How was missing data handled? requirement for surveys to have at least 50% survey response to be included in pooled data sets, but that still could be a lot of people missing.

How did authors adjust for missing data? did each country’s survey weights adjust for missing data?

Thank you for raising the important point of missing data. In the supplementary material to our manuscript, we have included a table on the missing predictor variables by country amongst the participants with dyslipidemia. As can be seen there, there is no substantial missingness in any of the variables – apart from education in Tokelau and diabetes status in Tonga. Because of this, these two countries had to be excluded from our predictor analysis of cascade completion. Regarding missing lipid biomarkers, please see response above. Furthermore, the country’s survey weights to adjust for nonresponse at the sampling stage. We have now added a section to our appendix that better explains the survey weights used in our analyses (see response on survey weights below and Appendix 3 in the supplementary material).

7.    Discussion is unfocused --see detailed comments in the attached full review .  The main points appear to be ~7% prevalence and largest drop off is lipid measurement--yet discussion organized as main points are treatmetn and control so seems mis-aligned.  Moreover, the discussion rambles to report other papers cascade % without any nuance of explaining or hypothesizing why.

We thank you for this assessment. We have reworked the discussion to better align it with our results and to make it more concise.

By section

Introduction

• gap is overstated: authors make two seemingly contradictory points in the introduction: 1) dyslipidemia is widely prevalent in LMIC (cite literature including GBD reports), and 2) “literature has been unable to provide accurate country prevalence estimates due to dearth of nationally representative data”

- GBD uses WHO Steps data which comprises part of the authors dataset
- authors overstate their claim that “literature has been unable to provide accurate country prevalence estimates“
- in fact, multiple GBD publications that the authors cite do exactly this, generate reliable population level estimates

We thank you for this important comment. We have removed claims about the availability of hypercholesterolemia prevalences and refocused the paragraph in question solely on the contribution of our paper, namely the access of hypercholesterolemia care. The paragraph now reads as follows (p. 6 lines 12-18):

“Despite the importance of rigorous evidence to guide health policy and improve healthcare delivery, the current empirical evidence remains weak and offers only a limited understanding of the state of care for hypercholesterolemia in LMICs.[8,9] Research is mainly confined to single countries, often based on a subnational level with a focus on specific subpopulations, or to single health care indicators, such as access to essential medicines.[8,10–13] To our knowledge, nationally representative studies analyzing broader health system performance at the individual-level across a larger number of LMICs have been altogether absent.”

• last sentence first paragraph—statement that high burden of dyslipidemia persist b/c of emphasis on communicable, maternal, and neonatal diseases.... That seems to be speculation. Could be due to lack of MOH funding for lab capacity that includes lipids (usually a send out lab in most LIC) for example. Sets up a narrative that may not have empirical evidence.

As suggested by the reviewer, we have now shortened the sentence (page 6 lines 9-10):

The fact that a high burden persists suggests that many health systems in LMICs are still ill-equipped to address this important condition~~, in part due to an emphasis on communicable, maternal, neonatal, and nutritional diseases.~~[8,9]

Methods

- Need clearer definitions of each step—do they include a time period—for example, lipids measured within the last year or within the last 10 years?

The definition of each step can be found in the methods section of our manuscript (page 9 lines 107-121) and the exact country-level question phrasing, upon which these definitions are based, can be found in our supplementary material. Whenever cascade stages refer to a specific time period, this is specified in both the method section and our supplementary material. In a small number of cases, the time period indicated differs in a country from the overall majority. This can be viewed and is discussed in our supplementary material.

- What is the definition of HTN—SBP 130 or 140 for all comers, for those with DM/CRF? Definition of DM also not provided.

Thank you for suggesting greater clarity here. An elaborate definition and measurement of the respondent’s diabetes and hypertension status are described elsewhere and the literature describing this is included in our manuscript. In order to more directly brief the reader on these definitions, we have also added the following sentence to our method (page 12 lines 166 - 173):

“The hypertension status was derived from blood pressure readings and the diabetes status from collected blood glucose measurements. Hypertension was defined as a systolic blood pressure of at least 140 mm Hg, diastolic blood pressure of at least 90 mm Hg, or reported use of medication for hypertension. Diabetes was defined as fasting plasma glucose of at least 7.0 mmol/l (126 mg/dl), random plasma glucose of at least 11.1 mmol/l (200 mg/dl), HbA1c of at least 6.5%, or reporting to be taking medication for diabetes. More details on the definition and measurement of these comorbidities are provided elsewhere [15,16]”

- page 8: how do point of care tests vs lab tests for lipids typically differ? is one systematically lower/higher than the other? Can this be stated as a short clause in the text, rather than in the appendix?

Thank you for raising this important point. As we have responded to Reviewer #3, literature shows that point-of-care-machines compare well against laboratory testing and can be reliably used for lipid screening [1–3]. One study by Ferreira et al. (2015) specifically compares the clinical agreement of the CardioCheck PA – which is used by the majority of countries – to the laboratory in sorting lipid records into the ATP III lipid classifications used in our manuscript [1]. They showed 94.6%-97.7% agreement depending on the biomarker measured. Therefore, we do not suspect a systematic bias in our analysis based on the different lipid measuring methods. With this in mind, we would like to point out that a bias could potentially arise from the fact that point-of-care machines usually have an upper cut-off above which lipids cannot be reliably measured. Because of this we are restricting our analysis to the range that these devices are equipped to report on (see Appendix 4). We also provide a supplemental analysis, in which we do not restrict on this, which can be seen in Appendix 7. If needed, we are happy to also provide disaggregated cascade analyses based on lipid measurement. However, as laboratory measurements are more expensive, we would argue that a disaggregation would likely speak more to the selection of countries that can afford laboratory testing rather than a bias in the measures themselves.

To make this point clearer in our manuscript as well, we have amended the discussion as follows (p.26 lines 430-435):

“In addition, our definition of hypercholesterolemia was based on biomarkers that, in some countries, were measured with point-of-care devices. While these may be less accurate than lab-based testing, studies have shown that they can be reliably used for lipid screening [29,58–60]. A study by Ferreira et al. (2015) found a 94.6%-97.7% agreement between the CardioCheck PA – which is used by the majority of countries – and the laboratory when sorting lipid records into the ATP III lipid classifications used in our analysis [60].”

- Poisson regression: add time fixed effects. 2009-2018 is a decade, could have been secular changes during this time.

We thank you for this suggestion. Our analysis includes survey fixed effects, which account for both time and country differences. It is unfortunately not possible to cleanly disentangle country and time effects with currently existing lipid data, as discussed in our limitation section. However, in an effort to provide readers with some intuition on this, we have now added an analysis that shows cascade estimates by year. While there is no clear time trend visible, this must be viewed with the caveat in mind that the estimates are based on a small number of surveys per year and that they are likely heavily disentangled with country effects. To make this point clearer, we have added the more precise phrasing of “survey fixed effects” rather than “country fixed effects” throughout our manuscript as well as added the following to our discussion and supplementary material:

Manuscript (p.26 lines 436-441):

“First, the comparability between countries is to some extent limited as the time span of 9 years across surveys potentially introduced period effects into our analysis. While a cascade analysis by year showed no observable time trend (see Appendix 7), this must be viewed in light of the fact that the estimates are based on a small number of surveys per year and that they are likely heavily enmeshed with country effects.”

Appendix:

Figure 12: Cascade of Care for High TC by Survey Year

See Note Figure 1; Hypercholesterolemia refers to all respondents that are classified as having high TC (≥240 mg/dL) or a self-reported medication status.

- How reliable is fasting documentation in the data?

We know the fasting status of 96.56% of our total sample. In the overall sample, 87.71% of respondents are fasting. However, we do not adjust lipid measures based on fasting status. A study by Nordestgaard (2017) in the Journal of the American College of Cardiology has shown that lipid profiles are largely unaffected by fasting status; random lipid profiles can be used for prognostic, diagnostic and therapeutic use without negative implications; and that the maximal mean change for non-fasting versus fasting TC and LDL-C is -8mg/dL[10].

Discussion

- Moreover, the gap in paragraph 2 seems to be ~ to high income countries which is not given enough discussion as to why.
- Overall the discussion is very long and very unfocused. For example, authors dedicate 1-2 paragraphs to each of screening, diagnosis, treatment, and control. These paragraphs have two components: repeating data from this study (and stating there is heterogeneity), and comparing this to data from other care cascade studies. Because of this structure, there is a lot of repetitiveness in 1) repeating data from study, and 2) repeated comparisons with other countries. Examples below:

• repeating data from this study:

o While there was substantial heterogeneity across countries – with screening rates ranging from under 10% in Benin, Kiribati, Myanmar, the Solomon Islands, and Zambia up to 89% in Iran – this stage was consistently found to be the largest or second largest stage of loss along the cascade of care.

o Again, large heterogeneities across the sample became apparent at this stage, as less than 10% of individuals with dyslipidemia in six countries reached this stage, less than 50% in 23 additional countries, and only six countries were able to retain more than 50% of individuals with dyslipidemia at the diagnosis stage.

o We found that 29% of those with high TC and 33% of those with high LDL-C had been given advice or medication for their high cholesterol. At the country level, this ranged from less than 5% up to 79%.

o Both in the pooled analysis and at the disaggregated level, control rates were found to be low, ranging from virtually zero to 27% in all but two countries – Iran (57%) and Morocco (49%).

• repeated comparisons with other cascade papers

o According to the US Centers for Disease Control and Prevention, in 2015/16, about 70% of all adults in the United States had received a cholesterol screening test in the past five years, whereas a study on dyslipidemia screening in nine European countries reported much lower rates – with only five countries reaching rates of over 50%, while numerous others fell below 20%

o Two earlier studies, one from China in 2001 and a second multi- country study that examined data on 40 to 79 year-olds from five high income countries and three middle-income countries over the period 1998-2007, found similarly low levels of diagnosis in Thailand and China.10,3

o Suggestions for stronger discussion:

▪ remove repeated presentations of data from this paper.
▪ Comparing cascade numbers between countries is not that interesting—what would be more interesting is an explanation of why these numbers differ

o Strongest paragraph in Discussion is on page 18.

As per your suggestion, we have reworked the discussion.

MINOR ISSUES

Introduction

- Ref 3 is from 2018, but text states data is from 2019

Thank you for pointing this out, we have updated the reference it accordingly.

- Ref 4 is from 2001 and likely out of date. Can authors find an updated article that makes a similar point?

We use reference 4 in defining hypercholesterolemia to be consistent in our definitions throughout our manuscript. However, as discussed in response to your other comments, we have added AHA/ACC guideline definitions to our manuscript as well (see response above).

- Page 7, Cascade Construction: again, ATP III is not the standard of care anymore, and has been superseded in the US by multiple subsequent reports by ACC/AHA. European guidelines have also evolved from Framingham data. References cited as evidence of wide use of ATP guidelines range from analyses published 2013-2014.

Thank you kindly for this very important point. Please see response above on adding ACC/AHA guidelines. The European Society of Cardiology and European Atherosclerosis Society (ESC/EAS) guidelines include a geographical parameter in defining hypercholesterolemia and therefore it was not feasible to apply these to our manuscript. But as noted in responses above, we have further added this to our discussion.

▪ if authors want to retain ATP III cut offs, should discuss how they compare to most up to date guidelines in Discussion, ie would more or less people meet criteria for dyslipidemia using up to date thresholds

Note response above.

- Ref 8 is from 2010. Can the authors find an updated article that makes a similar point?

As per your suggestion above, the text passage that this refers to has been dropped from them manuscript and therefore the reference is no longer relevant.

- this sentence is confusing, pages 7-8: “Second, as treatment guidelines are usually based on CVD risk scores, rather than on lipid measures alone, and vary across countries, we chose to be conservative in our definition of who is considered to be in need of care as to not evaluate health systems by unapplied care standards”

We have simplified the sentence to the following (p.9 lines 77-80):

Second, treatment guidelines are usually based on CVD risk scores rather than on lipid measures alone and often vary across countries. In order to not evaluate health systems by care standards that are in fact unapplied, we chose to be conservative in our definition of hypercholesterolemia.

Methods

- can authors clarify which weights they used from each country’s survey weights? Some have multiple depending on which data is examined (interview vs biochemical). This would help with transparency and reproducibility

We have added the following description to our supplementary material:

Appendix 2 (p.3-4 lines 72-101):

**“Country-specific Sampling Weights**

The STEPS datasets include three survey weights for each part of the survey instrument, including the interview (Step 1), physical measurements (Step 2), and biochemical measurements (Step 3), as different samples of participants are included in the three survey parts. STEPS weights are adjusted for the probability of selection, non-response, and differences between the sample population and the target population. Because the analyses in this study rely on lipid measurements as part of the STEPS instrument Step 3, Step 3 weights, referred to as WStep3, were applied in the analyses of STEPS data.

Although most of the datasets included in the study are STEPS surveys (32 STEPS surveys were included), for Chile the 2009/10 National Health Survey, for Seychelles the 2013 National Survey of Noncommunicable Diseases, and for the Marshall Islands the 2017 HYBRID Survey were used. In these surveys also specific weights for biochemical measurements were used where available.

The table below lists the weight variables used for the respective dataset:

| **STEPS Survey:** | **Weight Variable** |
| --- | --- |
| Algeria | wstep3 |
| Azerbaijan | wstep3 |
| Bangladesh | wstep3 |
| Belarus | wstep3 |
| Benin | wstep3 |
| Bhutan | wstep3 |
| Botswana | wstep3 |
| Burkina Faso | wstep3 |
| Costa Rica | wstep3 |
| Ecuador | wstep3 |
| Eswatini | wstep3 |
| Guyana | wstep3 |
| Iran | wstep3 |
| Iraq | wstep3 |
| Kiribati | wstep3 |
| Kyrgystan | wstep3 |
| Lebanon | wstep3 |
| Moldova | wstep3 |
| Mongolia | wstep3 |
| Morocco | wstep3 |
| Myanmar | wstep3 |
| Solomon Islands | wstep3 |
| Sri Lanka | wstep3 |
| St. Vincent and the Grenadines | wstep3 |
| Sudan | wstep3 |
| Tajikistan | wstep3 |
| Timor-Leste | 1* |
| Tokelau | wstep3 |
| Tonga | wstep3 |
| Tuvalu | wstep3 |
| Vietnam | wstep3 |
| Zambia | wstep3 |
|  |  |
| **Non-STEPS surveys** |  |
| Chile - 2009/10 National Health Survey | fexp_fac and fexp_ex |
| Seychelles - 2013 National Survey of Noncommunicable Diseases | wpop |
| Marshall Islands - 2017 HYBRID Survey | 1^+^ |

*Notes*: *As a STEPS survey country, the Timor-Leste data included wstep3. However, the weighted sample characteristics were not consistent with what would be expected from the population of Timor-Leste (or any population) and it was decided that unweighted data were used as the more conservative option.

^+^For Marshall Islands no sample weights were provided as the sampling was said to be representative of the population.

When weights were missing, the average weight was assigned to observations with missing weight values. Further, when observations had to be dropped from the sample because of missing values, for example, in covariates, the survey weights were adjusted proportionally.

In the main analysis of cascades, each country contributes equally to the estimations. To achieve this, the relevant weights of all datasets were rescaled so that the sum of weights in one dataset referring to one country equals 1. In analyses in which surveys should contribute to estimations by their respective country’s population size, weights were rescaled so that the sum of weights in one dataset equals the country’s population size.”

- page 7: can authors define cutoff values for the 3 classifications of total cholesterol and 5 classifications for LDLc

The cut-offs were added in a footnote.

- page 11: last paragraph in Statistical Analysis section doesn’t seem to belong there. Seems better suited to a Measurements section.

We have included a sub-heading for a better overview

Results

- given 41% sample is < 35 years, should probably report age standardized dyslipidemia prevalence too

Thank you for this suggestion. We have added a table to our appendix that provides an intuitive overview of hypercholesterolemia prevalences along the age distribution in this set of countries.

Appendix 5:

Table 5: High TC and High LDL-C Prevalences by Age Cohort

|  | Total Cholesterol Sample* | | LDL Cholesterol Sample** | |  |
| --- | --- | --- | --- | --- | --- |
|  | Prevalence | Confidence Interval | Prevalence | Confidence Interval |  |
| Overall | 7.1 | [6.8 , 7.4] | 7.5 | [7.1 , 7.9] |  |
| By Age |  |  |  |  |  |
| 15 - 24 y/o | 1.6 | [1.2 , 2.2] | 2.0 | [1.4 , 2.8] |  |
| 25 - 34 y/o | 3.4 | [2.9 , 4.1] | 3.8 | [3.1 , 4.5] |  |
| 35 - 44 y/o | 5.8 | [5.3 , 6.3] | 6.1 | [5.5 , 6.7] |  |
| 45 - 54 y/o | 10.8 | [10 , 11.6] | 10.6 | [9.6 , 11.7] |  |
| 55 - 64 y/o | 14.4 | [13.3 , 15.5] | 14.2 | [12.9 , 15.6] |  |
| 65+ y/o | 14.8 | [13.2 , 16.6] | 16.3 | [13.6 , 19.4] |  |
| Note: Prevalences account for sampling design with survey weights re-scaled by the survey’s sample size such that all countries contribute to estimates according to their population size. * Includes respondents from all 32 countries with a valid total cholesterol measurement;  ** Includes respondents from Algeria, Bangladesh, Burkina Faso, Chile, Costa Rica, Iran, Iraq, Lebanon, Mongolia, Morocco, Myanmar, Seychelles, and St. Vincent & the Grenadines with a valid LDL-C measurement | | | | |  |
|  |  |  |  |  |  |
|  |  |  |  |  |  |
|  |  |  |  |  |  |
|  |  |  |  |  |  |
|  |  |  |  |  |  |
|  |  |  |  |  |  |
|  |  |  |  |  |  |
|  |  |  |  |  |  |

- Fig 1: font very small, hard to read

We have increased the font size.

- page 12 type: Easter Mediterranean

Thank you for pointing out this spelling mistake, we have changed it accordingly.

- “multivariate Poisson regression” should be “multivariable Poisson regression”. See

https://www.ncbi.nlm.nih.gov/pmc/articles/PMC3518362/.

Thank you for pointing out this error. We have changed it accordingly.

- page 14, “less than two out of every five respondents with dyslipidemia had been treated...” but in Fig 1 the treated is 29% for High RC and 33% for High LDLc??

We have changed the sentences to the following (page 22 lines 316-318):

“In a pooled sample of 129,040 individuals from 35 LMICs, we found that less than one out of every three respondents with hypercholesterolemia had been treated and less than one in five had achieved control.”

# References

1.	Ferreira CE dos S, França CN, Correr CJ, Zucker ML, Andriolo A, Scartezini M. Clinical correlation between a point-of-care testing system and laboratory automation for lipid profile. Clinica Chimica Acta. 2015;446: 263–266. doi:10.1016/j.cca.2015.04.036

2.	Panz VR, Raal FJ, Paiker J, Immelman R, Miles H. Performance of the CardioChek PA and Cholestech LDX point-of-care analysers compared to clinical diagnostic laboratory methods for the measurement of lipids. 2005; 5.

3.	Plüddemann A, Thompson M, Price CP, Wolstenholme J, Heneghan C. Point-of-care testing for the analysis of lipid panels: primary care diagnostic technology update. Br J Gen Pract. 2012;62: e224–e226. doi:10.3399/bjgp12X630241

4.	Zou G. A Modified Poisson Regression Approach to Prospective Studies with Binary Data. American Journal of Epidemiology. 2004;159: 702–706. doi:10.1093/aje/kwh090

5.	Barros AJ, Hirakata VN. Alternatives for logistic regression in cross-sectional studies: an empirical comparison of models that directly estimate the prevalence ratio. BMC Med Res Methodol. 2003;3: 21. doi:10.1186/1471-2288-3-21

6.	Chen W, Qian L, Shi J, Franklin M. Comparing performance between log-binomial and robust Poisson regression models for estimating risk ratios under model misspecification. BMC Medical Research Methodology. 2018;18. doi:10.1186/s12874-018-0519-5

7.	Manne-Goehler J, Geldsetzer P, Agoudavi K, Andall-Brereton G, Aryal KK, Bicaba BW, et al. Health system performance for people with diabetes in 28 low- and middle-income countries: A cross-sectional study of nationally representative surveys. Wareham NJ, editor. PLOS Medicine. 2019;16: e1002751. doi:10.1371/journal.pmed.1002751

8.	World Health Organization. Global status report on noncommunicable diseases 2010. Alwan AAS, editor. Geneva: World Health Organization; 2011.

9.	Atun R, Davies JI, Gale EAM, Bärnighausen T, Beran D, Kengne AP, et al. Diabetes in sub-Saharan Africa: from clinical care to health policy. The Lancet Diabetes & Endocrinology. 2017;5: 622–667. doi:10.1016/S2213-8587(17)30181-X

10.	Nordestgaard BG. A Test in Context: Lipid Profile, Fasting Versus Nonfasting. Journal of the American College of Cardiology. 2017;70: 1637–1646. doi:10.1016/j.jacc.2017.08.006
