# Supplementary material for: Unmet need for hypercholesterolemia care in 35 low- and middle-income countries: A cross-sectional study of nationally representative surveys
Source: PLoS Med. 2021 Oct 25;18(10):e1003841. doi: 10.1371/journal.pmed.1003841 (PMC8575312; doi:10.1371/journal.pmed.1003841)
Supplement: Attachment — Submitted filename: Response to reviewers and editors.docx [file pmed.1003841.s013.docx]

Requests from Editors:

1)	In the abstract:

a)	Please remove the limitations from the Conclusion and include it as the last sentence of the abstract’s Methods and Findings section.

We have changed the abstract accordingly.

2)	Please title the summary you have provided after the abstract as "Author summary." Please see our author guidelines for more information: <https://journals.plos.org/plosmedicine/s/revising-your-manuscript#loc-authorsummary>.

We have titled it as such.

3)	In the main text, please provide p-values in addition to 95% CI where appropriate.

We have added p-values where appropriate.

4)	Please provide the total N in tables 1 and 2

We have included total N in Table 1. Table 2 already included total N.

5)	For your Tables and figures, please do the following:

a)	Please indicate in the figure caption the meaning of the bars and whiskers

We have taken the explanation of the bars and whiskers out of the figures note and now indicate it above the note within the figure caption.

b)	Please define the abbreviations such as BMI, TC, LDL-C, WHO PEN , PSU, ATP III

We have defined the abbreviations.

Comments from Reviewers:

Reviewer #1: The authors have made great efforts to address the reviewers' comments and I appreciate that. I think the authors have addressed my comments and other reviewers' comments very well. The responses are respectful, clear and in details.

I only have one follow-up comment on presenting probability rather than RR or OR for the regression model. I think you misunderstood my suggestion. I did not ask for a linear probability model. What I am proposing is that even if you fitted the data using modified poisson (or logistic) regression, you can always present the effect of an intervention in term of change in the probability of the outcome instead of RR or OR by using the coefficients from the fitted model and some simulation techniques. I think probability is much more intuitive to most non-academic people than RR or OR is.

That being said, I understand the reason why you used modified Poisson and I agree that presenting RR is common and is fine.

We thank you kindly for your comment and for further clarifying your thoughtful suggestion. Given that you find RR acceptable as well, we choose to continue to use only these, in order to keep the manuscript as concise as possible.

Thank you again for taking your time to review our work.

Reviewer #2: I appreciate the authors' work on the revision. I only have two minor comments. Defining "LDL-C measurement of 70 mg/dL or higher as having hypercholesterolemia" is very aggressive (as this is not unanimously accepted definition in all patients even with the current ACC/AHA guidelines), but I suggest phrasing this analysis as assessing how results vary across the spectrum.

We thank you for your comment. We agree that LDL-C measurements of 70mg/dl is a rather aggressive definition and therefore only use it in a supplementary definition. Our main analysis is based on ATP III guidelines, which are more conservative.

On page 10: "TC was smaller than 200 mg/dL and LDL-C was smaller than 122 130 mg/dL." I assume you meant lower than and not smaller than.

Thank you for pointing this out. We have changed it from “smaller” to “lower”.

Thank you again for taking your time to review our work.

Reviewer #3: The authors have addressed all comments of this Reviewer.

Thank you again for taking your time to review our work.

Reviewer #4: Authors respond the vast majority of suggested revisions adequately and paper is strengthened including additional analyses and clearer description of limitations and inference.

Thank you again for taking your time to review our work.
